# Evidence for Co-Evolution between Human MicroRNAs and Alu-Repeats

**DOI:** 10.1371/journal.pone.0004456

**Published:** 2009-02-11

**Authors:** Stefan Lehnert, Peter Van Loo, Pushpike J. Thilakarathne, Peter Marynen, Geert Verbeke, Frans C. Schuit

**Affiliations:** 1 Gene Expression Unit, Department of Molecular Cell Biology, Katholieke Universiteit Leuven, Leuven, Belgium; 2 Department of Molecular and Developmental Genetics, VIB, Leuven, Belgium; 3 Department of Human Genetics, Katholieke Universiteit Leuven, Leuven, Belgium; 4 Bioinformatics group, Department of Electrical Engineering, Katholieke Universiteit Leuven, Leuven, Belgium; 5 Biostatistical Centre, U. Z. Sint-Rafaël, Katholieke Universiteit Leuven, Leuven, Belgium; University of Western Cape, South Africa

## Abstract

This paper connects Alu repeats, the most abundant repetitive elements in the human genome and microRNAs, small RNAs that alter gene expression at the post-transcriptional level. Base-pair complementarity could be demonstrated between the seed sequence of a subset of human microRNAs and Alu repeats that are integrated parallel (sense) in mRNAs. The most common target site coincides with the evolutionary most conserved part of Alu. A primate-specific gene cluster on chromosome 19 encodes the majority of miRNAs that target the most conserved sense Alu site. The individual miRNA genes within this cluster are flanked by an Alu-LINE signature, which has been duplicated with the clustered miRNA genes. Gene duplication events in this locus are supported by comparing repeat length variations of the LINE elements within the cluster with those in the rest of the chromosome. Thus, a dual relationship exists between an evolutionary young miRNA cluster and their Alu targets that may have evolved in the same time window. One hypothesis for this dual relationship is that these miRNAs could protect against too high rates of duplicative transposition, which would destroy the genome.

## Introduction

Alu is the most abundant short interspersed nuclear element (SINE) of the human genome, occupying 10% of the genome content with a copy number estimated to be at least 1.3 million. It is anticipated that the Alu integration rate has been variable over time and is nowadays at less than 1% of the rates encountered 40 million years ago [1]. Extensive Alu transpositions were probably required to be fixed in a breeding population. It was determined that a minimum of 2n insertions allow one insertion to be fixed in a breeding population of n individuals [2]. A balance must exist, however, between selfish expansion of the repeat and catastrophic destruction of the host genome. Therefore only the repeat elements that evolved mechanisms to control their own amplification rate in order to minimize deleterious effects on the host, will be efficient in the long run to amplify [2]. Here we propose a model for such a self-controlling mechanism, involving Alu repeats and microRNAs. Alu multiplies when an active Alu element is transcribed and the encoded RNA is integrated at a new target site. Some of these integrations occur in exons of protein-encoding genes [3], both parallel and anti-parallel to the transcription unit (henceforth called sense and antisense, respectively). Sense and antisense Alu integration occurs also in the proximity of microRNAs (miRNAs), as miRNAs can be co-transcribed in long primary transcripts [4].

MicroRNAs are 19 to 22 nucleotide long non-coding RNAs that influence gene expression by repressing translation or by causing mRNA degradation [5], [6]. The fact that the miRNA and RNAi pathways share most proteins and mediate both endonucleolytic cleavage underlines their similarities [7]–[9]. Protection against viruses and transposons was suggested as a natural function of the RNAi pathway [10]. That miRNAs could interact with repetitive elements was proposed by showing that a number of miRNA-encoding genes contain at the 5′-end seed sequences that are complementary to Alu sequences [11], [12]. Less is known about the function of repeat elements in the human genome, besides that several studies indicate the involvement of these elements with genome structure and gene expression [13], [14], Alu elements proved to be a useful set of tools for phylogenetic analyses [15]. The relationship between Alu sequences and miRNAs was recently extended by work from Zhang and colleagues, who showed that 7 miRNA pairs of a miRNA cluster on Chr19 (C19MC) are linked to Alu repeats which facilitated the expansion of C19MC [16]. This model shows similarities with our research work.

Here, we propose that C19MC is the result of duplications of one repeat core cassette containing one miRNA and four repeats. Comparing variations of repeat characteristics of the C19MC region to the entire sequence of Chr19 supports this. On the basis of the sense Alu target selection of the miRNAs in C19MC and the role that Alu fulfilled in the growth of C19MC, a model of dual relationship between Alu elements and miRNAs can be proposed. At a time of high Alu transposition activity, Alu facilitated growth of the miRNA cluster, which by its targeting properties influenced the life cycle of Alu so that duplication rates declined.

## Results

### Sense but not antisense Alu sequences are enriched in miRNA target sites

As an initial exploration of the relationship between Alu and miRNA, we analyzed miRNA 5′-end seed complementary to Alu sequences. For this initial screen all human miRNAs listed in miRBase 10.0 were selected. We limited our search to perfect seed matches, for the following three reasons: First, previous studies indicated that the most essential binding nucleotides defining the targets of a miRNA (the so called “seed”) are located at bases 2–8 of the mature miRNA [17], [18], second most current target prediction algorithms rely heavily on this seed sequence [19]–[21], and last the conservative nature of this approach. We looked at Alu elements in sense and anti-sense orientation as depending on the strand of integration either one of the two possible orientations can manifest in a transcript.

**Figure 1:**
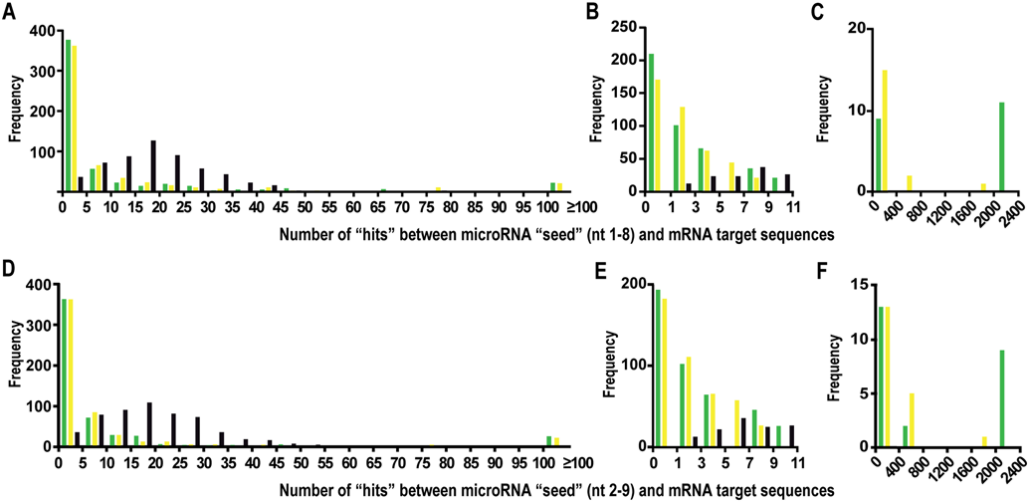
Frequency of target sites per miRNA (A–F) in Alu-containing human transcripts. The depicted frequency is normalized to 1 megabase of sequence. All RefSeq non-redundant human transcripts containing at least one Alu repeat (or a fragment of an Alu repeat) in the mature transcript were used here, separating Alu sequences in the sense orientation (green), Alu sequences in the antisense orientation (yellow) and mRNAs depleted of Alu sequences (black). Possible miRNA target sites are defined here as perfect miRNA “seed” matches of nucleotide 1–8 (panel A–C) or nucleotide 2–9 (panel D–F). (A, D): complete frequency distribution; (B, E) and (C, F): focused view on the region of the distribution with low and high number of predicted target sites, respectively. X aches indicate bin limits; each bin contains values including the smaller limit and excluding the higher limit (e.g. 0≤x<5); except the last bin of panel (A, D) where all values are grouped that are equal to or exceed 100 (x≥100).

We first looked for miRNAs having perfect seed complementary sites (nucleotides 1–8 and 2–9) to sequences within three sequence collections, extracted from the RefSeq collection of mature human mRNAs: i) sense Alu-elements, ii) antisense Alu-elements and iii) Alu-depleted mRNA sequence (Figure 1A–F; see also Materials and methods). This led to the observations that the majority of miRNAs did not show a single hit against the Alu sequences and that a limited number of miRNAs showed more than 1000 hits per megabase against sense Alu sequences. To test whether this enrichment might be a result of selective pressure, we performed several controls which indicated that miRNAs having a high hit rate in sense Alu sequences and having a low rate in antisense Alu sequences is neither explained by chance nor by sequence bias (Figure S1).

### Hotspots in the Alu sequence are targets for miRNA seeds

**Figure 2:**
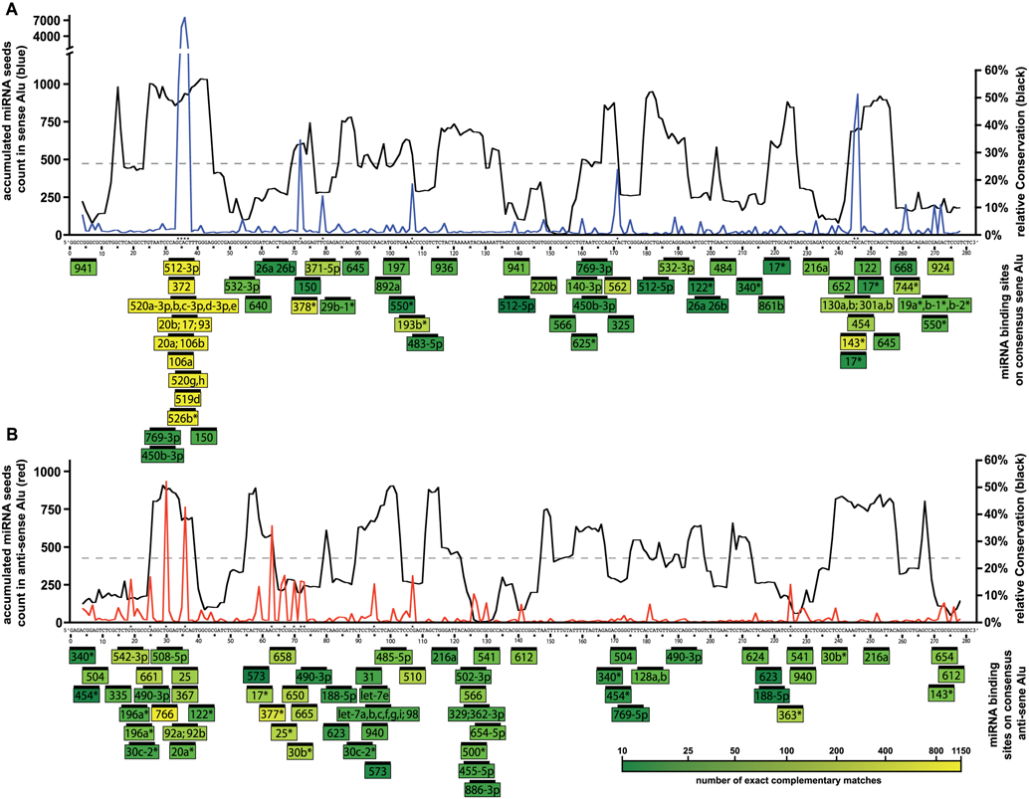
Sense Alu miRNA targets concentrate in areas with minimal sequence variation, antisense Alu miRNA target sites in areas with high sequence variation. A sequence window of 8 nucleotides was moved over the sense Alu consensus (panel A) in order to measure (i) the total number of target sites defined by all human miRNAs seeds (both nucleotides 1–8 and 2–9) (blue line), and (ii) the level of conservation of the consensus Alu sequence (black line), the average conservation as a gray dashed line. Asterisks above the consensus sequence indicates sense Alu target sites that have over 250 “hits”. Below the consensus sequence, the miRNAs targeting the Alu sequences are shown using a color code that reflects target frequency (Supplementary Methods online). The most strongly recognized target site coincides with the best-conserved area of human Alu (nucleotides 34–37). The miRNA target sites and sequence conservation are shown for antisense Alu sequences (panel B). The panel design is s similar to (A), total numbers of target sites are illustrated by a red line, corresponding to antisense orientation of the Alu elements.

In order to identify potential hotspots of miRNA recognition in the Alu sequence, we aligned all Alu repeats present in mRNAs to the Alu consensus sequence (again separating sense and antisense Alus), plotted the average conservation and overlaid this with the miRNA target sites detected (Figure 2). Most prominent in these graphs was the region of nucleotide 34–37 in the sense Alu sequence, as this is both the least variable area (identical in all Alu subfamilies), and a target hotspot for 17 miRNA seeds (each ∼900 “hits” per miRNA; Figure 2A). Other hotspots for miRNA seeds that had a high frequency (>250 “hits”; asterisks Figure 2A), were centered at nucleotide positions 72, 107, 171 and 245, also coinciding with conserved areas of sense Alu. Thus, 9 out of 10 sense Alu target sites that have over 250 “hits”, have a miRNA that is directed against evolutionary conserved Alu sequence. This is in sharp contrast to antisense Alu sequences, where out of 12 sites with a high total frequency (>250 “hits”), 7 correspond to areas that exhibit the highest variability among subfamilies (asterisks Figure 2B). Hence, unlike for the sense orientation, where the concurrence of high target numbers per miRNA in well-conserved regions suggests a general Alu targeting strategy, miRNAs targeting antisense Alu sequences avoid the most conserved regions. Generation of miRNAs targeting sense Alu sequences may thus have been a relevant early event in the expansion of the Alu family in primates.

Ten out of the 17 miRNAs having seed complementarity against the most prominent sense Alu target region are encoded in a miRNA cluster on Chr19 (C19MC). MiRNA 372 is targeting this conserved spot as well. This miRNA belongs to the miRNA family mir-371, 2, 3 which is conserved from humans to rodents and was recently proposed to be the origin of the miRNA Cluster on Chr19 [16]. Both Alu and the miRNA of C19MC are only found in primates. The next question was, therefore, if there was a non-random link between Alu and the individual miRNA genes in this cluster.

### Alu targeting 3p-miRNAs are dominating within the miRNA cluster on Chr19

**Figure 3:**
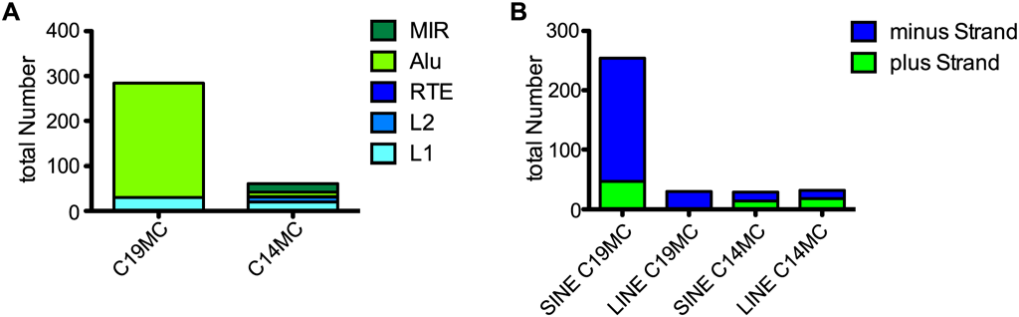
Comparison of repeat content between miRNA clusters on Chr14 and Chr19. Panel A) shows the absolute number of repeats found in each cluster divided for SINE and LINE elements, subdivided by repeat family. The absolute number of the strand of integration (plus and minus strand) from SINE and LINE elements is shown in Panel B).

MiRNAs are produced from a ∼85 nt precursor. The miRNA precursor (pre-miRNA) is folded to a hairpin like structure of which each side of the double stranded stem has the capability of forming a mature miRNA, called thereon 5p- and respectively 3p-miRNA depending on their relative position on the single stranded precursor transcript (see [5] for Review). Figure S2 shows alignment of pre-miRNAs of C19MC. Sequence distance between the 5p-miRNA region (distance 0.14) is lower as compared to the 3p-miRNA region (distance: 0.21). We then aligned all mature miRNAs of C19MC and found 31 3p-miNRAs vs. 23 5p-miRNAs. This is relevant, because all miRNAs that target the most conserved sense Alu region are 3p-miRNAs (purple boxes in Supplemenatry Figure 3). 9 of the 31 3p-miRNAs of C19MC target the cluster. When we expand our analysis to include imperfect seed matches by allowing one mismatch (which may be functional: [5]), this number was extended to 19.

A pre-miRNA does not always produce two mature miRNAs: one may be a minor product or not detectable at all [5]. Only two out of 31 3p miRNAs are classified as “minor product”, while 8 out of 21 for 5p miRNAs (asterisks in Figure S3) are minor products. In addition, we expected the source pre-miRNA to produce two mature miRNAs per pre-miRNA. We did so as most of the aligned pre-miRNAs (26 out of 38) and as the more distant related pre-miRNAs (miRNA 498, 512 and 515) to C19MC code for two miRNAs per precursor. Out of the 12 pre-miRNAs that give rise to only one mature miRNA however, 9 produce exclusively the 3p miRNA, while only 3 give rise to 5p miRNAs. This is interesting especially as the 3p region of the pre-miRNAs with higher sequence distance was more successful in maintaining miRNA production capability.

Together, these observations suggest an evolutional driving force for amplifying 3p miRNAs in the primate C19MC of which many members have 3p miRNA seeds against the most conserved sense Alu target sequence.

### C19MC is enriched in minus strand SINE repeat elements

We next analyzed the nature of the repeats in C19MC as these were recently proposed to be contributing to duplication events during evolution of the cluster [16]. As a control, we studied C14MC, the biggest known mammalian miRNA cluster which is on human chromosome 14 [22]. We found that these two clusters differ extensively in their repeat element composition. As shown in Figure 3, the repeat composition of C14MC approximated the expected situation of the whole genome, (equal amounts of LINE and SINE integrations on the plus and minus strand). The situation on C19MC was very different. For C19MC, a difference in repeat class content (∼90% SINE vs. ∼10% LINE) as well as in strand of integration was seen (∼80% SINE and 100% LINE minus Strand; Figure 3). Thus, a much more asymmetric SINE and LINE distribution was seen for C19MC than for C14MC. As is illustrated in Figure S4, (red arrow) this remarkable asymmetry of plus/minus strand distribution in the miRNA cluster also contrasts with another control, namely the rest of chromosome 19.

### Reduced repeat length variation and orientation preference within C19MC suggest that a gene duplication cassette is implicated in the growth of the miRNA Cluster

**Figure 4:**
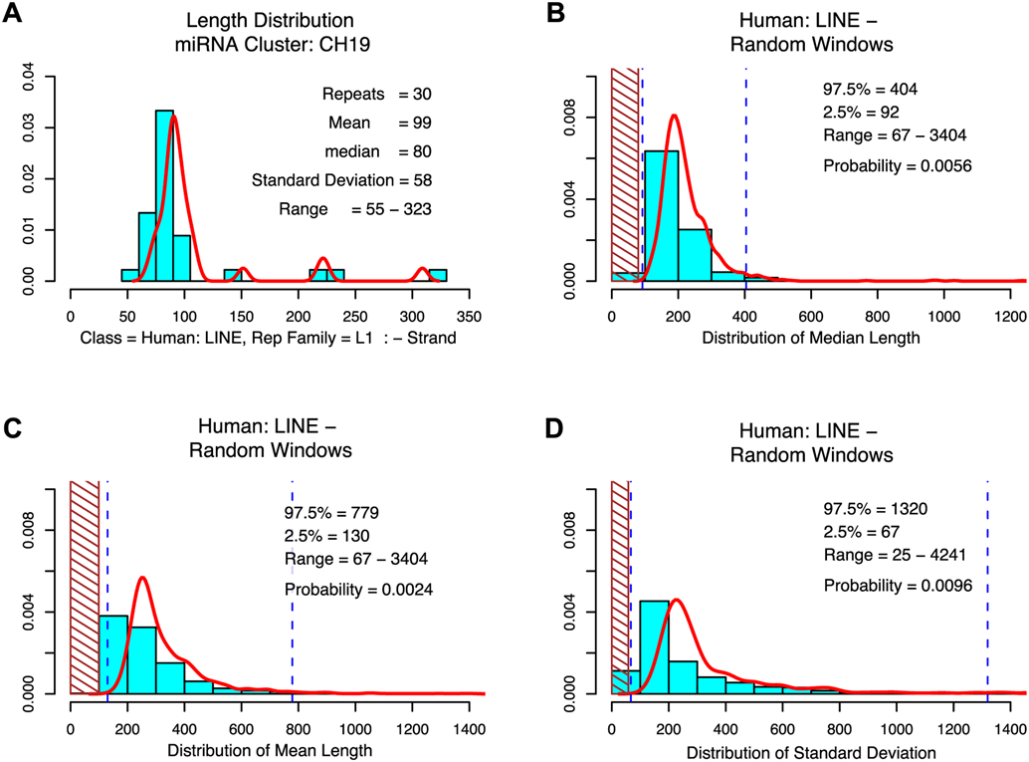
LINE minus strand repeat length variation is significantly smaller in C19MC compared to the rest of chromosome 19. Panel A) Length distribution of C19MC of human LINE minus strand integrations. The superimposed red curved line indicates the kernel density estimate for the distribution. Panel B) Shaded areas indicate the region where mean length of the random windows is smaller than the mean length of C19MC. The dashed lines indicate the 2.5% and 97.5% percentiles of the mean length distribution of random windows. P-value is the proportion of mean length numbers which are smaller than the mean length of C19MC. Panel C) and D) similar to B) but the distribution of the Mean (C) and respectively the standard deviation (D) were computed.

To further assess this asymmetry, we compared the 100 Kb C19MC sequence next to the repeat content and plus/minus strand distribution of 1,250 randomly selected 100 Kb fragments on Chromosome 19. If, for C19MC, repeat elements are duplicated in a cassette, we expected them to be uniform in length when compared to repeats that integrated randomly in the genome. In contrast, when the repeats would not be part of the duplicated cassette a size distribution would be expected. To quantify this, we compared the mean, median and standard deviation of the length of the repeat elements in C19MC to the distribution of the same characteristics in the randomly selected windows of 100 Kb. Table 1 shows the summary of probabilities (*p*) for SINE and LINE elements in three primate species. Refining extreme characteristics within the miRNA cluster in these primates would show that the repeats within the region of the miRNA cluster where present since formation of the cluster and not gained after diversion of the primate species. Distributions for the medians, and standard deviations in the random windows, as well as the distribution of the length of the human LINE minus strand elements are shown in Figure 4 (human SINE repeats: Figures S5 (minus strand) and S6 (plus strand)).

**Table 1:** Length distribution of LINE and SINE repeats in primate C19MC.

	LINE−		SINE−		SINE+	
***Homo sapiens***						
number	30	NS	208	***	49	NS
median length	80	**	167	*	287	NS
SD of length	58	**	93	NS	85	NS
***Pan troglodytes***						
number	29	NS	192	*	44	NS
median length	81	**	172	*	293	NS
SD of length	56	*	88	NS	83	NS
***Macaca mulatta***						
number	22	NS	133	**	260	NS
median length	78	*	165	*	275	NS
SD of length	28	*	86	NS	97	NS

Total number of repeats and size distribution of the 100 Kb chrosomose 19 miRNA cluster region compared to 1250 randomly selected 100 Kb fragments from the same chromosome within the indicated species. P values representing probabilities of the C19MC distribution being the same as in the random samples were calculated by the relative frequency of occurrence of extreme outcomes: NS: P>0,05; * P>0,05; ** P<0,01; *** P<0,001. Please note that the median length of the SINE minus strand elements is about half the size of the plus strand elements in all three primates. Results for LINE plus strands are not shown, as these were not found within C19MC.

For SINE plus strand elements, no difference was observed between C19MC and randomly selected fragments of Chromosome 19 with respect to length distribution (Table 1). The number of repeats (49) in the human C19MC for the SINE plus strand elements is also in line with what was observed for the randomly selected windows. In contrast, length distribution of the SINE minus strand as well as the LINE minus strand elements was shifted much more uniform in the cluster than in other regions of Chr19. Furthermore, the median length of LINE and SINE minus strand repeats was significantly shorter (Table 1). In fact, we noted that the median length of the SINE minus strand elements was about half the size of the plus strand elements in all three primate species.

**Figure 5:**
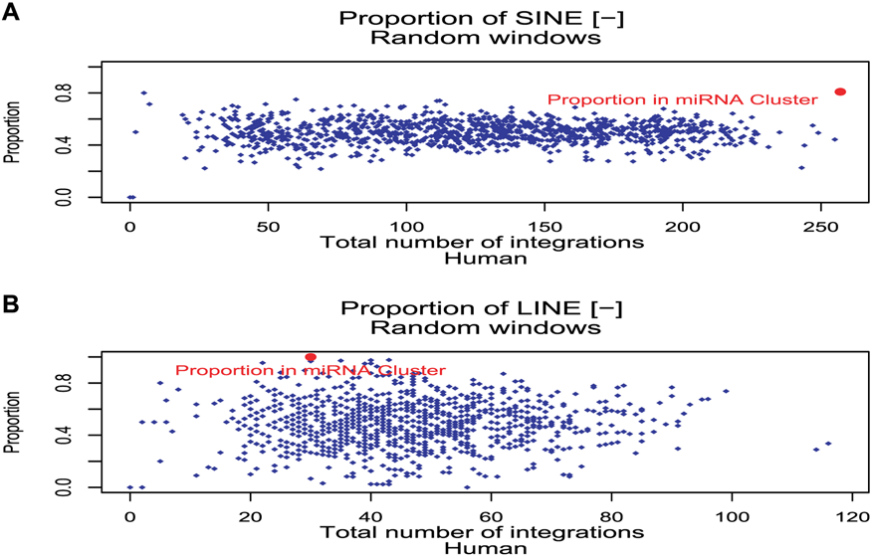
Orientation of SINE and LINE repeats in C19MC is not balanced over plus and minus strands. The analysis is based on the human genome, 1250 random windows of 100 k lengths are shown (total sequence length corresponds to ∼2× length of Chr19). For each window the proportion of SINE (A) and LINE (B) elements is computed with respect to the total integration for that window (blue rhombus; 0 equals 100% of integration on the plus Strand; 1 equals 100% of integration on the minus Strand). Proportions are plotted in regard to their number of total repeat integrations of the corresponding class per window in ascending order from left to right. Red dot: proportion of the miRNA cluster on Chr19 (C19MC), blue dots: random windows.

It is known that the integration rate of repeats within a genomic region can vary, depending on the gene density of that region [23]. However while the total integration rate per given region may vary, no preference for a strand of integration has been reported so far. Therefore we expected to find comparable amounts of repeats on the plus and minus strand, if their integration occurred on a random basis. We plotted the proportion of SINE and LINE elements per random selected 100 Kb window and sorted them for their total number of repeat integrations, as we expected to find equal proportions to be more likely to occur at higher repeat numbers. For SINE elements an equal strand of integration rate was found for most windows (proportion 0.5±0.15; Figure 5A). A more diverse strand of integration rate was seen for LINE elements (Figure 5B). However in both cases C19CM strand preference of integration (red dot) is an extreme outliner compared to the random samples of the same chromosome (blue rhombuses). A similar observation was made for data collected from the Chimpanzee and Rhesus Monkey Chr19 (see Figure S7 respectively).

Thus, both the outspoken strand preference of orientation and more uniform length of minus strand LINE and SINE elements make it unlikely that many of the repeats within C19MC integrated independently from each other.

### SINE and LINE minus strand repeats but not plus strand repeats belong to the common miRNA duplication cassette

Three years ago it was reported that many of the miRNAs of C19MC are embedded in long (400–700 nucleotide) cassettes that are repeated along the cluster [24]. A cassette of 400–700 nucleotides holds the miRNA precursor and a region that could resemble a miRNA promoter sequence. Seeing the unusual arrangement of repeats in the region of C19MC we propose that the flanking ALU and LINE repeats belong to that duplicated cassette. To support this claim, we computed a multiple alignment of 38 sequences to what we propose is the common core of the C19MC gene duplication cassette (Figure S8). This cassette consists of an Alu element (alignment position (ap) 1290) followed by a short (80 nt) L1M4 element, a miRNA promoter region, the pre-miRNA and two distal Alu fragments. Within the proximal Alu element two distinct subgroups are seen on basis of an alternative central 18 nt sequence (Figure S9). The central decrease in L1M4 sequence similarity is explained by the T rich sequence. A third Alu element is found downstream of the two distal Alu elements belonging to the cassette that shows a much higher sequence variation and distinct subgroups within the alignment. Higher sequence variation of the Alu elements was described previously as a marker for gene duplication events. In this model, gene duplication events are facilitated by Alu sequences, so called junction Alus, that duplicated adjacent sequences regardless of their relative orientation and position to that sequence [25]. Hence Alu elements in this 3p block of high similarity could have helped to facilitate growth of this miRNA Cluster. Zhang et al.'s work supports this theory describing the role of these Alu elements within the C19MC. Their computational analysis of the cassettes from miRNA 502 g and 520 h revealed a junction Alu at exactly these Alu elements [16].

Together, a combination of three Alu elements and one short LINE together with the miRNA genes seems to have shaped a gene cassette that successfully grew during primate evolution. The number of cassettes in the cluster varies between different primate species. Of interest is that these core duplication cassettes contain only minus strand Alu elements. In contrast, all miRNAs within C19MC are encoded in the plus strand.

## Discussion

In this study we have analyzed several characteristics of the miRNAs and repetitive elements within C19MC. The miRNAs of C19MC showed high seed complementarity to Alu sense sequences. A classical hypothesis for seed sequence complementarity is that the miRNAs of C19MC, like all other miRNAs target mRNAs. Targets for miRNAs encoded in C19MC are listed in different miRNA gene target databases. Many of these targets are involved in signal transduction and nucleic acid binding [16]. During our own analysis of miRNA target sites within mRNAs, we found that over 2000 mRNAs contain Alu element fragments with about the same number of sense and antisense integrations. Especially rich in Alu elements were for example Zinc finger proteins, a group of nucleic acid binding proteins. It was proposed a while ago that Alu elements embedded in mRNAs are probable miRNA targets [11], [12], but this awaits experimental validation [26]. It is possible that miRNA targets in mRNAs-embedded Alu elements have proven beneficial for primates in a second stage of development - the reason for acquiring these miRNAs in the first place could have been a completely different one: namely a defense mechanism against Alu transposition. This is in line with our observation that only sense but not antisense Alu sequences are targeted at high frequencies, but both Alu versions are kept with equal numbers in exons and are therefore encoded in mature mRNAs.

C19MC is one of the imprinted loci that contain small non-protein coding RNA genes, which are considered important for cell fate decisions in the early embryo [27]. In agreement with this idea, are the miRNA genes of C19MC highly and specifically expressed in placenta and some also in testis (Figure S10), suggesting their role in Alu surveillance. In contrast, other miRNA genes with seed sequences against sense Alu, but encoded outside C19MC, are more widely expressed. Thus, from these expression data it can be proposed that the evolutionary young miRNA gene cluster C19MC plays a role in guarding against deleterious effects of Alu on the genome. The fact that a subset of miRNAs of C19MC is also highly expressed in testis [28] is of particular interest, as at least one in every 50 sperm cells was estimated to be subject to a retrotransposition event [2], [29]. This expression pattern is compatible with a surveillance role of the miRNAs of C19MC to prevent catastrophic Alu duplications in germline cells. Specific interaction between two mammalian miRNA's and a retroposon-like non-coding RNA (RTL1) have already been demonstrated [30].

This work described a repeated gene duplication cassette containing a miRNA and four repeats that resemble a miRNA cluster on Chr19. Clustered miRNAs can be transcribed in polycistronic transcripts [31]. A polycistronic transcript of C19MC would hold several Alu elements, as three of four repeats per duplicated cassette are Alu elements. Only 20% of all Alu element integrations within the C19MC are plus strand integrations, the same strand as miRNAs within C19MC are integrated. Eighty percent however are minus strand Alu integrations that were mainly gained by the core miRNA gene duplication cassette. Therefore most Alu will be found in a primary miRNA transcript as an antisense Alu, a sequence that can be targeted by the miRNAs encoded within this cluster. This may be relevant, as a primary transcript encoding both miRNAs that target sense Alu and the sense target in tandem, would titrate its own miRNAs away, preventing the possibility to target the free Alu RNA in the cell.

However, although sense or plus strand Alu elements are not included in the core miRNA gene duplication cassette, they could still play a central role in sensing Alu activity. Borchert and colleagues showed that sense Alu transcription of C19MC can pass the Alu element and continue transcribing downstream miRNAs [4].

**Figure 6:**
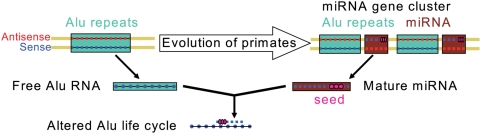
Model for dual relationship between Alu elements and miRNAs in the C19MC cluster. During the phase of rapid extension of Alu copy number, a miRNA containing cassette was duplicated from which the mature miRNA targets free (duplicating) Alu RNA. As the number of duplicated miRNA genes in the cluster grew, growth rates of Alu declined, preventing catastrophic destruction of germline genome information by Alu.

The unique design of the cluster's miRNA duplication cassette, as well as the cluster's sense Alu target capacity and the miRNA expression data were the basis to postulate a dual interaction model (Figure 6). In this model, homology sites of Alu sequences helped duplicating a gene cassette encoding miRNAs, which in turn can target sense Alu sequences and thus alter the fate of free Alu elements. This is of great interest as Alu is a retro-element that can transpose in a cycle containing a free Alu transcript, which is always in sense orientation. This model includes on one hand the fact that 3p-miRNAs of C19MC are enriched in number and production quantity and on the other hand that gene duplication events leading to growth of the cluster was facilitated by minus strand Alu repeats. Because a similar cluster is found in other primates [16], [24], which share Alu repeats with humans, it can be proposed that Alu expansion and growth of this cluster has occurred in parallel. Because many of the Alu elements within C19MC are evolutionary old (AluJ and AluS), expansion of the cluster may have occurred at an early wave of expansion of the Alu elements.

Based upon our bioinformatics analysis of miRNAs and repeat elements in the largest primate-specific miRNA cluster, we propose that a dual relationship exists between Alu elements and miRNAs. On the one hand, duplication events involving Alu sequences contributed to growth of the miRNA cluster and facilitated expression of these miRNAs. On the other hand the expressed miRNAs from the cluster are often predicted to target free Alu transcripts and it seems conceivable that this is beneficial for the host, by preventing catastrophic or self-destructive intensities of Alu retroposition.

Giving that much of this co-evolution between Alu repeats and the miRNAs of C19MC occurred about 40 million years ago, and that nearly all L1 elements lost transposition capability since then, experimental evidence for this co-evolutionary hypothesis will be hard to find today. Finding a second example of co-evolution between clustered miRNA genes and SINE repeats in another mammalian clade would add further support for this hypothesis.

## Materials and Methods

### Selection of Alu sequences

RefSeq mRNAs (NM_ number indexed) that contain Alu sequences were selected from assembly of June 2006 (http://www.ncbi.nlm.nih.gov/). Transcript variants were excluded while the mRNAs having highest Alu sequence content were kept. No difference was found in the Alu sequence composition between Alu elements integrated in exonic versus intronic sequence. The total mRNA sequence length in RefSeq was 64.2 megabases (without transcript variants); 14.4%, or 9.2 Mb was in Alu containing mRNAs. This selection of Alu containing mRNAs was based upon detection of Alu repeats by Repeatmasker (http://www.repeatmasker.org), using standard settings, and was checked using the UCSC genome browser (http://genome.ucsc.edu/). To confirm results, we additionally used two Alu repeat consensus sequences. First an Alu consensus sequence generated from Alu containing mRNAs and second an Alu consensus sequence obtained from RepBase (http://www.girinst.org/repbase/index.html, Release 11.09). From these 9.2 Mb of Alu containing human mRNA sequences, we generated three subsets of mRNA sequence for further analysis: (i) Alu(fragment)-derived mRNA sequence in the sense orientation (compared to the orientation of the mRNA) (0.43 Mb), (ii) Alu(fragment)-derived mRNA sequence in the antisense orientation (0.45 Mb) and (iii) the remaining mRNA sequence, depleted of Alu(fragments) (8.38 Mb).

### Prediction of miRNA target sites

Prediction of miRNA target sites was done by scanning for perfect “seed” complementarity sites. Seeds (nt 1–8 and 2–9) of all 470 human miRNAs listed in the Sanger miRNA Registry (http://microrna.sanger.ac.uk, version 10.0), were selected. Targets were defined on basis of perfect Watson and Crick base-pairing. In the analysis of the number of target sites per miRNA, multiple target site predictions in the same sequence were counted as separate hits, and the total number of hits for each miRNA seed was normalized per megabase of sequence. In the analysis of the number of miRNAs with perfect seed matches to Alu consensus and randomized Alu sequences, multiple target site predictions in the same sequence were counted as only one prediction.

### Generation of randomized Alu sequences

Third order Hidden Markov Models were used to generate “randomized” Alu sequences. These models were trained with 50 Alu sequences (or fragments) (totaling 10 kilobases in length), extracted from randomly selected mRNAs in sense and antisense orientation respectively. This approach ensures that single nucleotide frequencies as well as di-, tri- and tetranucleotide frequencies are approximately equal to the nucleotide composition of the Alu sequences used for training the model.

### Annotation of miRNA target sites in aligned Alu sequences

All Alu sequences that were present in non-redundant mature mRNAs of RefSeq (http://www.ncbi.nlm.nih.gov/; Assembly June 2006) were separated into the sense and anti-sense orientation and aligned separately to the Alu consensus sequence using LAGAN [32]. We visualized the level of conservation of these aligned Alu sequences by counting the number of aligned perfect matches to an 8 base pair moving window of the Alu consensus sequence [33]. These data were normalized to the total number of Alu sequences aligned (1782 sense Alu sequences and 1712 antisense Alu sequences respectively). Target sites for miRNAs in these aligned sequences were predicted as described above, using perfect seed matches to nucleotides 1–8 or 2–9. Because the position of these target sites was annotated, target sites detected using both octanucleotides (1–8 first, 2–9 second) but being shifted by one base pair could be combined to one predicted target site. Multiple target sites within the same sequences at different positions were allowed. MicroRNAs were visualized at their predicted target site on these alignments if they fulfilled the combination of three selection criteria: (i) the miRNAs were required to have 73 or more predicted target sites in the sense or antisense alignment set; this threshold was chosen because it approximately represents the mean plus 5 standard deviations of the number of predicted miRNA target sites in Alu-depleted mRNAs (combining data from figures 1 and 2, the mean was 19.5, and the standard deviation was 10.7); (ii) the number of predicted miRNA target sites on sense or antisense Alu sequences was at least two times the number of predicted target sites on Alu-depleted mRNAs for that miRNA; (iii) at least 20% of the total miRNA target sites were found at the depicted position.

### Generation of multiple Alignments

Multiple alignments were calculated with ClustalX version 1.83.1 (ftp.embl-heidelberg.de) using the standard settings. MiRNA precursor sequences and mature sequences where taken from the Sanger miRNA Registry (version 10.0). 1000 nt flanking sequences of each miRNA precursor were selected from UCSC human assembly hg18. The ∼2000 nt long sequences were sorted after repeat distribution patterns, for their visualization customized Genbank formatted feature files were generated and imported into Vector NTI (Invitrogen). Subgroups were stepwise aligned, manually optimized and thymine rich DNA patches removed. The generated profile was used to guide and build a final alignment of the miRNA duplication cassettes with optimized flanking sequences with a length of 1300 nt upstream and 700 nt downstream. The total similarity of the multiple alignment was calculated from the miRNA subset having similar 2000 nt sequences using the build in analysis “Similarity” of the Vector NTI v10 AlignX program (Invitrogen). Excluded from the subset were sequences of miRNAs 512, 515, 498 as no sequence similarity was found in the flanking sequence. MiRNA precursor sequences of the same subset were selected to compute the mean percentage divergence of the divergence of all possible pair-wise alignments, from (i) the complete miRNA precursor sequence, (ii) and (iii) the corresponding sequence region of mature 3p and respectively 5p miRNAs of all precursor sequences (ClustalX). This subset was further used to align the mature miRNA sequences. However, sequence divergence of the mature miRNAs of precursor 515-1 and 515-2 were small enough to be included in the alignment.

### Length distribution of LINE and Alu repeats

The chromosomal coordinate defining start of each miRNA precursor or Alu sequence (plus strand; end for minus strand) were selected, separated by the strand of integration and grouped into bins of 25000 nucleotides length (Sanger miRNA Registry V10.0; Human Genome Repeatmasker annotations hg18, http://genome.ucsc.edu/). The average frequency of every two bins is shown in figure S4.

### Repeat content of CHR19 in randomly selected windows of 100 kb

Random start points for a window of 100 Kb where selected from the total length of Chr19, with the exception of the 100 Kb flanking ends of the chromosome to exclude telomere sequence regions. Computation of position was based on the genome build hg18 (Human), pantro2 (Chimp) and rheMac2 (Rhesus monkey). We rebuild the human chr19 out of the whole chimp and respectively rhesus genome, as there is no one to one relation between the chromosomes of different species and pieces of altering size can be exchanged. Repeat information was selected from the monkey chromosomes corresponding to the regions defined by the human alignment file of chr19 and further analyzed. Start and endpoint of the aligned sequence region to the human chr19 were corrected not to excise the given window size.

Repeat elements of C19MC were analyzed by computing their median length and standard deviation. These were compared to the distribution of the same characteristics in the randomly selected windows. A probability *p* expressing how extreme each characteristic is with respect to that distribution is reported. *p<0.05* was considered extreme. For describing distributions, the median was preferred over the mean, since the distributions are extremely skewed, and the mean can be severely affected by extreme observations, while the median is more robust.

## Supporting Information

Figure S1 Frequency of target sites per octanucleotide (A–C) in Alu-containing human transcripts and frequency of miRNA complementary sites per Alu-consensus sequences (D, E). The depicted frequency is normalized to 1 megabase of sequence. All RefSeq non-redundant human transcripts containing at least one Alu repeat (or a fragment of an Alu repeat) in the mature transcript were used here, separating Alu sequences in the sense orientation (green), Alu sequences in the antisense orientation (yellow) and mRNAs depleted of Alu sequences (black). Possible target sites are defined as perfect matches between octanucleotides and Alu-containing human transcripts, the octanucleotide set consist out of all possible combinations of eight nucleotides. (A): complete frequency distribution; (B) and (C): focused view on the region of the distribution with low and high number of predicted target sites, respectively. Note that a large proportion of octanucleotides does not occur in Alu repeats. X aches indicate bin limits; each bin contains values including the smaller limit and excluding the higher limit (e.g. 0≤x<5); except the last bin of panel (A) where all values are grouped that are equal to or exceed 100 (x≥100). Panel D, E shows that a part of the human miRNA repertoire is complementary to the sense Alu consensus sequence. The abscissa shows the number of human miRNAs that target one particular sequence; the ordinate shows the frequency (% of all tested sequences) at which this number was observed. Perfect seed matches of nucleotides 1–8 (D) and 2–9 (E) are taken as predicted miRNA target sites. Red bars represent miRNAs targeting the 31 consensus sequences that cover the currently known major sense Alu subfamilies (http://www.girinst.org/repbase/index.html, Release 11.09); the blue bars represent miRNAs targeting the 31 reverse complements of these. As a control for this experiment, we performed perfect seed match analysis on 100.000 randomized sense (white bars) and antisense (black bars) Alu sequences with Alu like base composition.
(0.37 MB TIF)

Figure S2 Distances of aligned pre-miRNAs and the 3p and 5p regions. Pre-miRNAs of C19MC are aligned (miR-498, 512-1,-2 and 515-1,-2 excluded). Mean percentage distance of the whole pre-miRNAs (distance 0.16) and the 5p- (distance 0.14) and 3p mature miRNA regions (distance 0.21) is computed from this alignment. Pink boxes indicate the region of 5p and 3p mature miRNAs (from left to right).
(2.03 MB TIF)

Figure S3 19 out of 31 3p miRNAs have seed complementarity to Alu. A Multiple Alignment of mature miRNAs of the 3p and 5p arm of the primate specific cluster on Chr 19 was computed (panel (A), (B) respectively; miRNA 512 and 498 excluded). 3p region of 8 nt Seed matches to the major conserved spot of sense Alu sequence are highlighted (perfect seed match in purple, seed match with one mismatch pink).
(0.96 MB TIF)

Figure S4 Distribution of miRNAs and Alu repeats on Chr19. The average number of miRNAs panel A), B) and Alu repeats panel C), D) on Chr19 plus strand panel A), C) and minus strand panel B), D) is shown. Please note that the region of the primate specific miRNA cluster contains about 5-fold more anti-sense than sense Alu repeats (red arrow (panel A, D)).
(0.76 MB TIF)

Figure S5 SINE minus strand Repeat length variation is significantly smaller in C19MC compared to Chr19. Panel A) Length distribution of C19MC of human SINE minus strand integrations. The superimposed red curved line indicates the kernel density estimate for the distribution. Panel B) Shaded areas indicate the region where mean length of the random windows is smaller than the mean length of C19MC. The dashed lines indicate the 2.5% and 97.5% percentiles of the mean length distribution of random windows. P-value is the proportion of mean length numbers that are smaller than the mean length of C19MC. Panel C) and D) similar to B) but the distribution of the Mean (C) and respectively the standard deviation (D) were computed.
(0.60 MB TIF)

Figure S6 Human SINE plus strand Repeat length variation of C19MC compared to Chr19 is shown. Analysis similar to supplementary figure 5 but is based on human SINE plus stand integrations.
(0.54 MB TIF)

Figure S7 Repeats C19MC are unequally distributed over the two DNA strands. The analysis is based on the Chimpanzee genome (Panel A, B) and Rhesus Monkey (Panel C, D). 1250 random windows of 100 k lengths are shown (total sequence length corresponds to ∼2× length of Chr19). For each window the proportion of SINE (A, C) and LINE (B, D) elements is computed with respect to the total integration for that window (blue rhombus; 0 equals 100% of integration on the plus Strand; 1 equals 100% of integration on the minus Strand). Proportions are plotted in regard to their number of total repeat integrations of the corresponding class per window in ascending order from left to right. Red dot: proportion of the miRNA cluster on Chr19 (C19MC), blue rhombuses: random windows.
(0.71 MB TIF)

Figure S8 Alignment of C19MC miRNA duplication cassettes. Each sequence consists out of one miRNA precursor with additional sequence of 1300 nt upstream and 700 nt downstream. The common core duplicated cassette is starting from alignment position (ap) ∼1290 onwards where a Alu fragment of ∼250 nt length is found. The next block of high conservation contains ∼80 nt short remains of a L1 element (ap ∼1770). This block is followed by a block containing a ∼370 nt sequence which resembles a kind of miRNA promoter sequence (starting at ap ∼2520) and the ∼85 nt precursor miRNA (starting at ap ∼2895). The cassette ends with two Alu element fragments of ∼80 nt and ∼150 nt length starting at ap ∼3200 and ∼3370.
(1.51 MB PDF)

Figure S9 Similarity blot of the aligned miRNA duplication cassettes from Chr19. The similarity of the alignment is shown per window of 8 nt. For visualization annotated duplicated cassettes of selected miRNAs are shown.
(0.57 MB TIF)

Figure S10 Human expression profile of Alu-targeting miRNA genes. Figure modified from [1]. Depicted miRNAs correspond to the miRNAs targeting sense Alu most often recognized and evolutionary most conserved target site (nucleotide 34–37, Figure 2, yellow marked). In addition, all members of the primate specific miRNA cluster on Chr19 C19MC are listed, as we proposed common driving force for gaining these miRNAs. Further the miRNA family members of miRNA cluster 371 are shown for two reasons. First as this cluster is positioned downstream of C19MC showing that some miRNAs of Chr19 are not highly expressed in placenta; second it was proposed that C19MC originated from miRNAs of this cluster [2]. An almost exclusive expression of C19MC miRNAs is seen in the placenta followed by the expression of a minor number of C19MC miRNAs in testis. In contrast miRNAs of Cluster 371 are not detectable in these two tissues. Two of the more ubiquitously expressed Alu targeting miRNAs (miR-93 and miR-17 of “other miRNAs”) are also detectable in placenta. As these are highly expressed in the hematopoietic system it is plausible that the signals detected in placenta origin from blood cell contamination. MiRNAs having an 8 nt seed match to the major conserved spot on sense Alu sequence are underlined (perfect seed match in purple, seed match with one mismatch in pink). 1. Landgraf P, Rusu M, Sheridan R, Sewer A, Iovino N, et al. (2007) A mammalian microRNA expression atlas based on small RNA library sequencing. Cell 129: 1401–1414. 2. Zhang R, Wang YQ, Su B (2008) Molecular evolution of a primate-specific microRNA family. Mol Biol Evol.
(0.66 MB TIF)
