# Supplementary figures and images for: Evidence for Co-Evolution between Human MicroRNAs and Alu-Repeats
Source: PLoS One. 2009 Feb 11;4(2):e4456. doi: 10.1371/journal.pone.0004456 (PMC2637760; doi:10.1371/journal.pone.0004456)

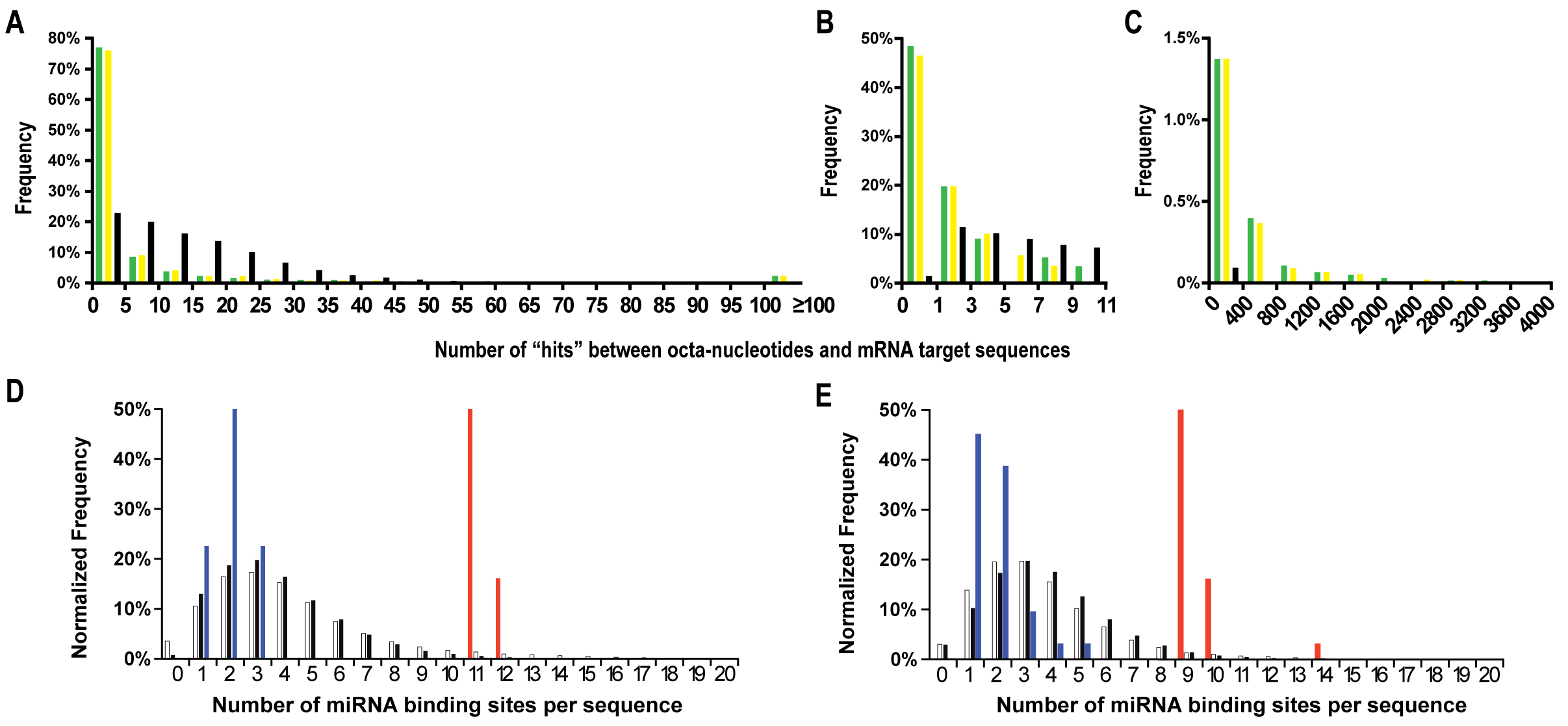

Supplement: Figure S1 — Frequency of target sites per octanucleotide (A–C) in Alu-containing human transcripts and frequency of miRNA complementary sites per Alu-consensus sequences (D, E). The depicted frequency is normalized to 1 megabase of sequence. All RefSeq non-redundant human transcripts containing at least one Alu repeat (or a fragment of an Alu repeat) in the mature transcript were used here, separating Alu sequences in the sense orientation (green), Alu sequences in the antisense orientation (yellow) and mRNAs depleted of Alu sequences (black). Possible target sites are defined as perfect matches between octanucleotides and Alu-containing human transcripts, the octanucleotide set consist out of all possible combinations of eight nucleotides. (A): complete frequency distribution; (B) and (C): focused view on the region of the distribution with low and high number of predicted target sites, respectively. Note that a large proportion of octanucleotides does not occur in Alu repeats. X aches indicate bin limits; each bin contains values including the smaller limit and excluding the higher limit (e.g. 0≤x<5); except the last bin of panel (A) where all values are grouped that are equal to or exceed 100 (x≥100). Panel D, E shows that a part of the human miRNA repertoire is complementary to the sense Alu consensus sequence. The abscissa shows the number of human miRNAs that target one particular sequence; the ordinate shows the frequency (% of all tested sequences) at which this number was observed. Perfect seed matches of nucleotides 1–8 (D) and 2–9 (E) are taken as predicted miRNA target sites. Red bars represent miRNAs targeting the 31 consensus sequences that cover the currently known major sense Alu subfamilies (http://www.girinst.org/repbase/index.html, Release 11.09); the blue bars represent miRNAs targeting the 31 reverse complements of these. As a control for this experiment, we performed perfect seed match analysis on 100.000 randomized sense (white bars) and antisense (black [file pone.0004456.s001.tif]

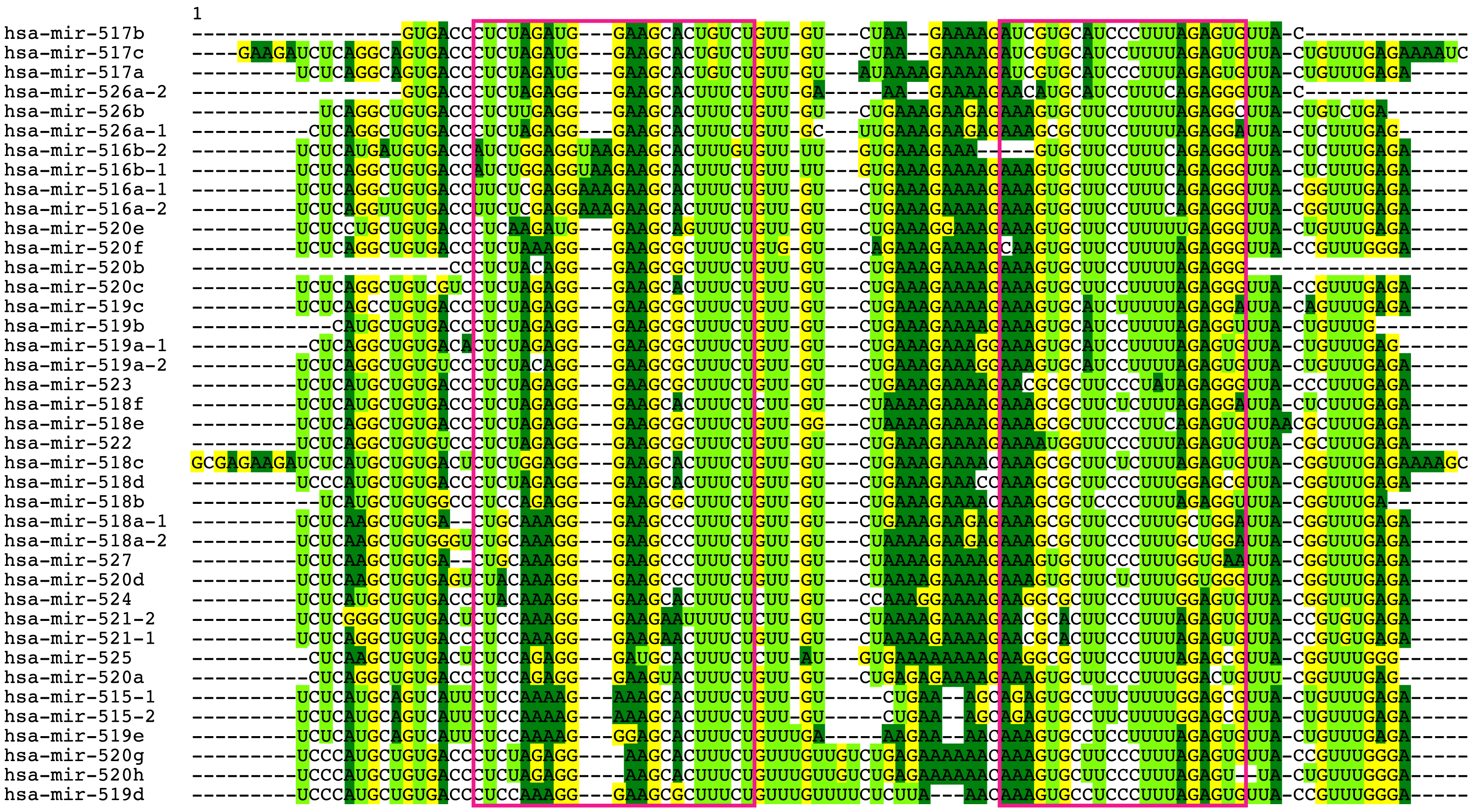

Supplement: Figure S2 — Distances of aligned pre-miRNAs and the 3p and 5p regions. Pre-miRNAs of C19MC are aligned (miR-498, 512-1,-2 and 515-1,-2 excluded). Mean percentage distance of the whole pre-miRNAs (distance 0.16) and the 5p- (distance 0.14) and 3p mature miRNA regions (distance 0.21) is computed from this alignment. Pink boxes indicate the region of 5p and 3p mature miRNAs (from left to right). (2.03 MB TIF) [file pone.0004456.s002.tif]

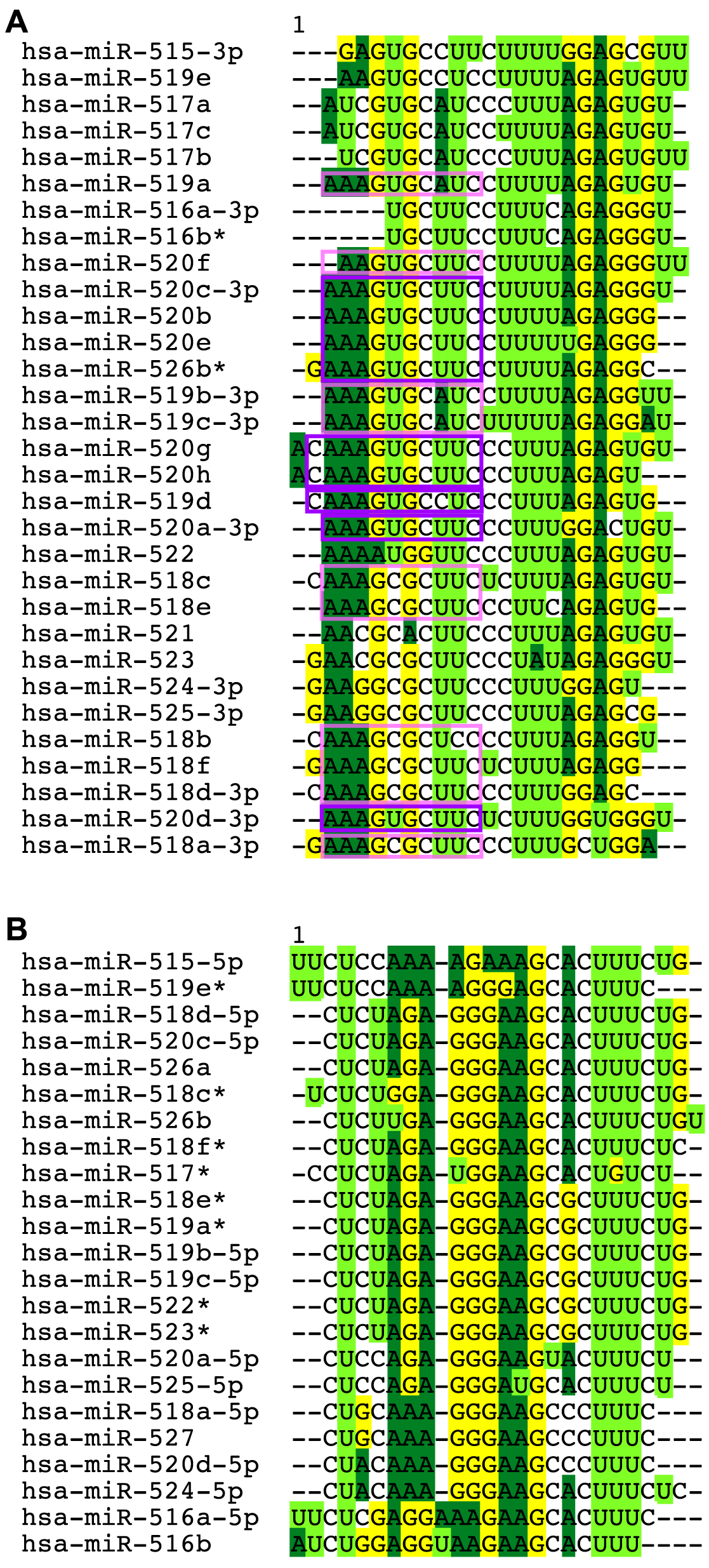

Supplement: Figure S3 — 19 out of 31 3p miRNAs have seed complementarity to Alu. A Multiple Alignment of mature miRNAs of the 3p and 5p arm of the primate specific cluster on Chr 19 was computed (panel (A), (B) respectively; miRNA 512 and 498 excluded). 3p region of 8 nt Seed matches to the major conserved spot of sense Alu sequence are highlighted (perfect seed match in purple, seed match with one mismatch pink). (0.96 MB TIF) [file pone.0004456.s003.tif]

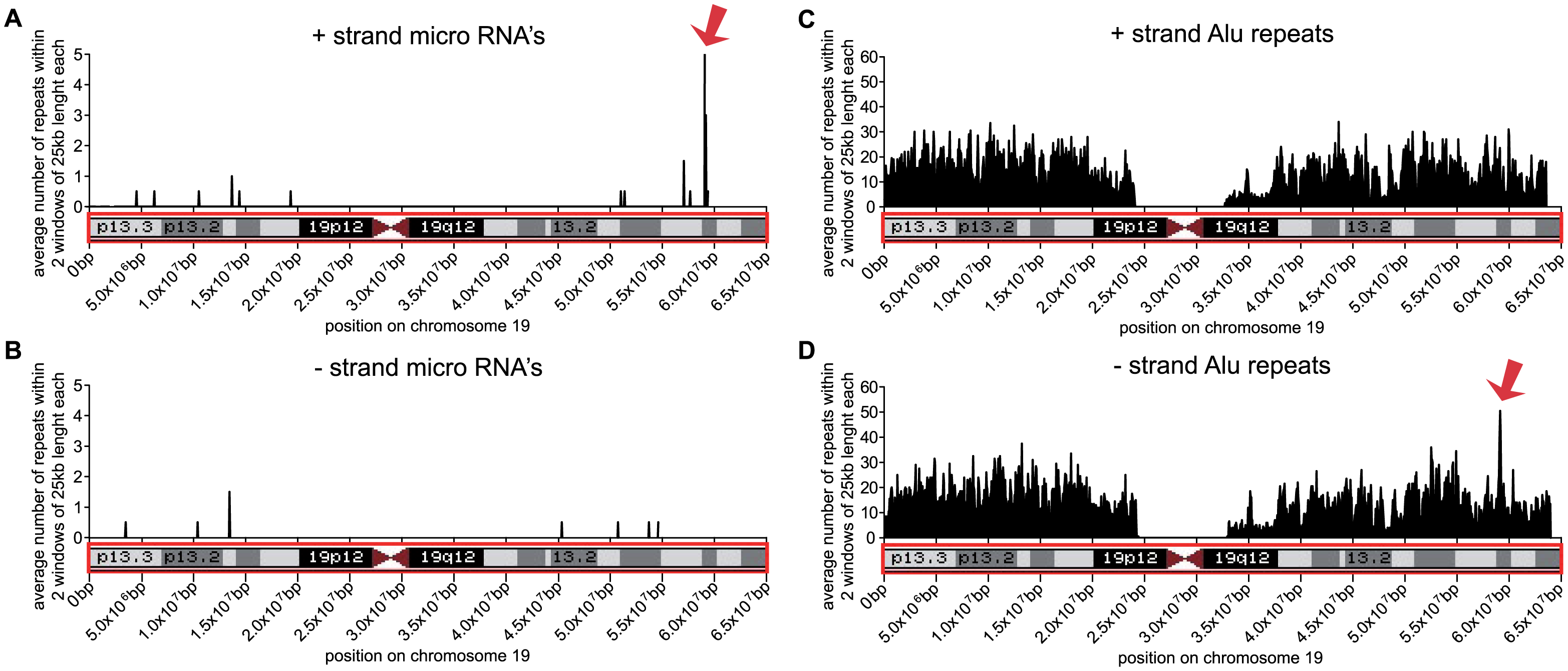

Supplement: Figure S4 — Distribution of miRNAs and Alu repeats on Chr19. The average number of miRNAs panel A), B) and Alu repeats panel C), D) on Chr19 plus strand panel A), C) and minus strand panel B), D) is shown. Please note that the region of the primate specific miRNA cluster contains about 5-fold more anti-sense than sense Alu repeats (red arrow (panel A, D)). (0.76 MB TIF) [file pone.0004456.s004.tif]

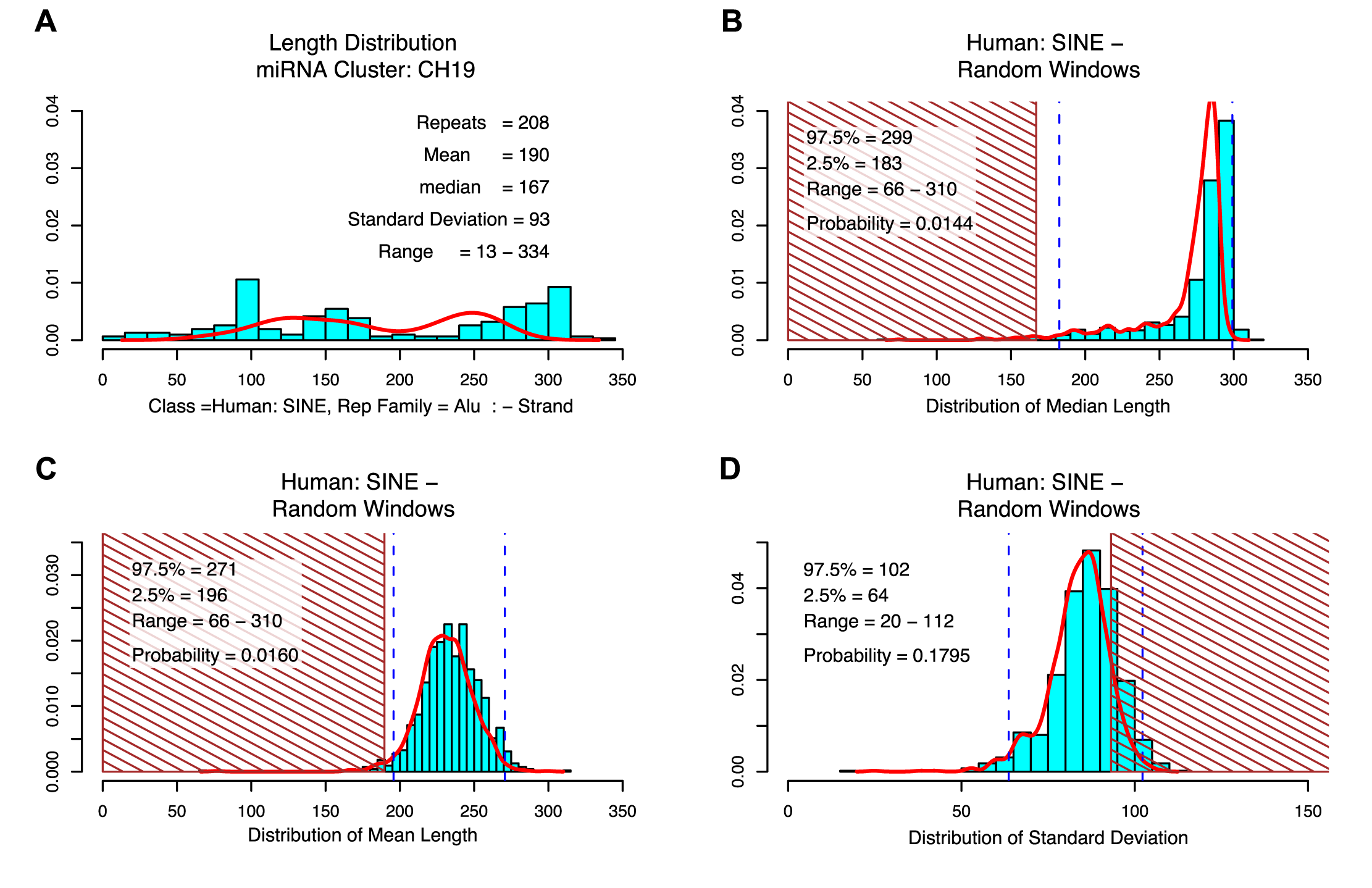

Supplement: Figure S5 — SINE minus strand Repeat length variation is significantly smaller in C19MC compared to Chr19. Panel A) Length distribution of C19MC of human SINE minus strand integrations. The superimposed red curved line indicates the kernel density estimate for the distribution. Panel B) Shaded areas indicate the region where mean length of the random windows is smaller than the mean length of C19MC. The dashed lines indicate the 2.5% and 97.5% percentiles of the mean length distribution of random windows. P-value is the proportion of mean length numbers that are smaller than the mean length of C19MC. Panel C) and D) similar to B) but the distribution of the Mean (C) and respectively the standard deviation (D) were computed. (0.60 MB TIF) [file pone.0004456.s005.tif]

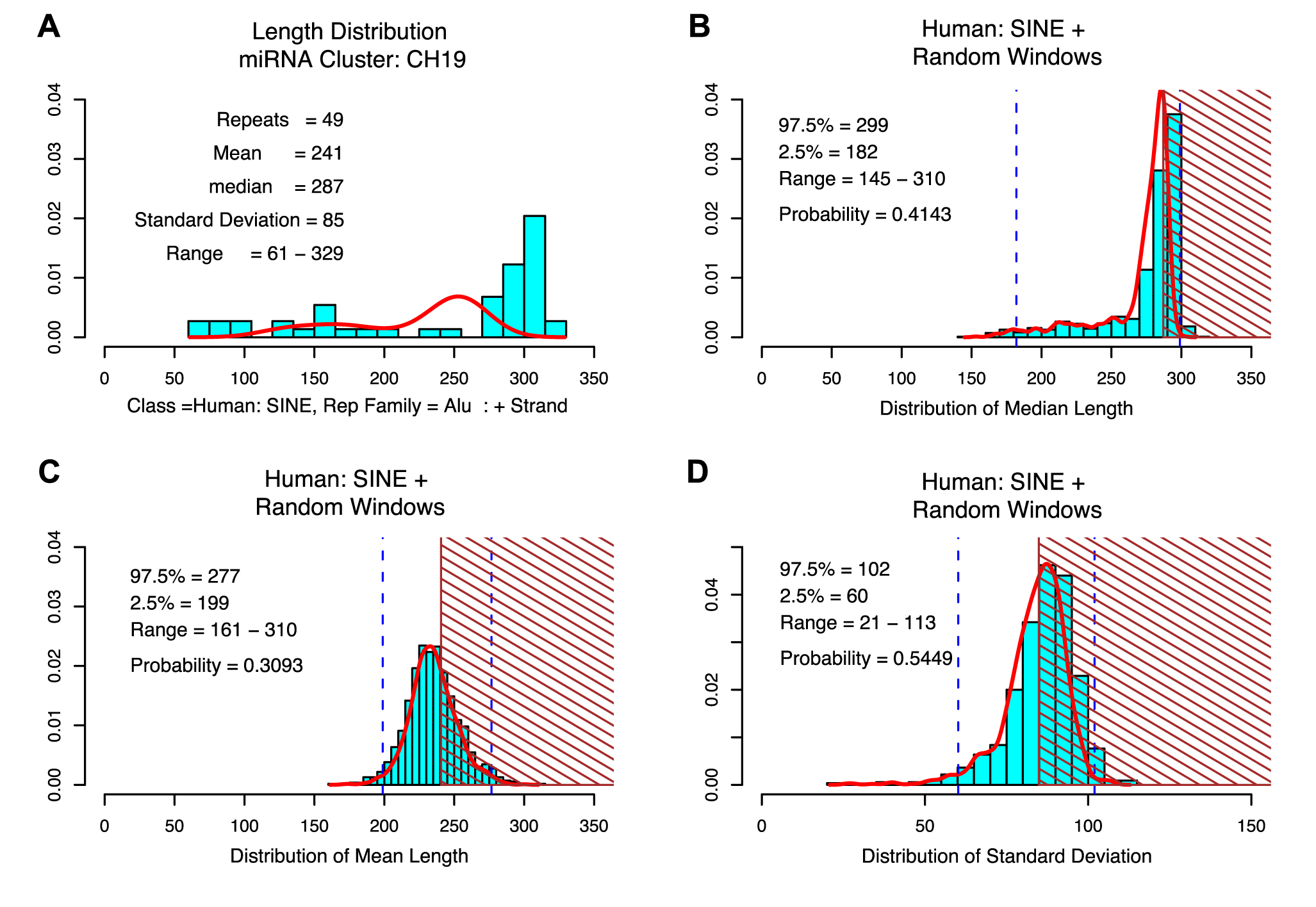

Supplement: Figure S6 — Human SINE plus strand Repeat length variation of C19MC compared to Chr19 is shown. Analysis similar to supplementary figure 5 but is based on human SINE plus stand integrations. (0.54 MB TIF) [file pone.0004456.s006.tif]

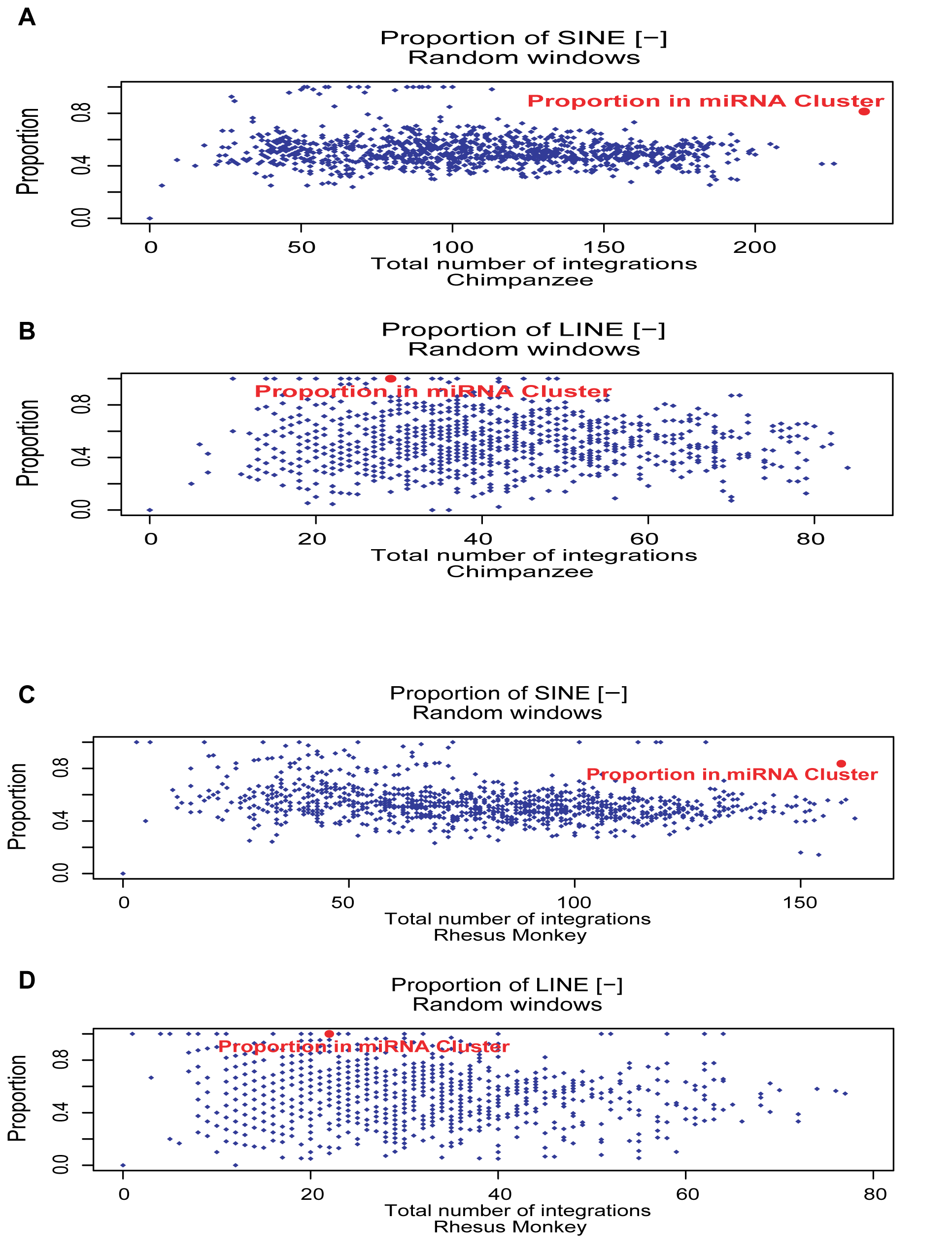

Supplement: Figure S7 — Repeats C19MC are unequally distributed over the two DNA strands. The analysis is based on the Chimpanzee genome (Panel A, B) and Rhesus Monkey (Panel C, D). 1250 random windows of 100 k lengths are shown (total sequence length corresponds to ∼2× length of Chr19). For each window the proportion of SINE (A, C) and LINE (B, D) elements is computed with respect to the total integration for that window (blue rhombus; 0 equals 100% of integration on the plus Strand; 1 equals 100% of integration on the minus Strand). Proportions are plotted in regard to their number of total repeat integrations of the corresponding class per window in ascending order from left to right. Red dot: proportion of the miRNA cluster on Chr19 (C19MC), blue rhombuses: random windows. (0.71 MB TIF) [file pone.0004456.s007.tif]

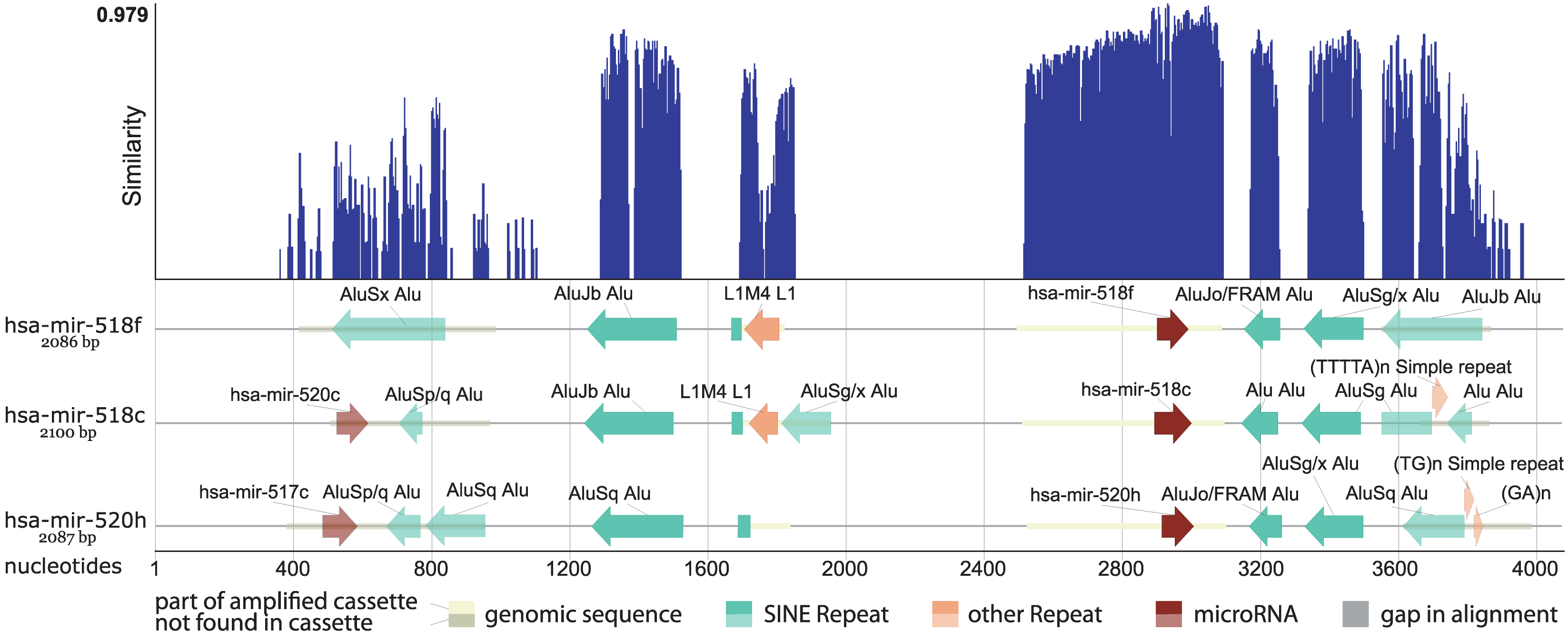

Supplement: Figure S9 — Similarity blot of the aligned miRNA duplication cassettes from Chr19. The similarity of the alignment is shown per window of 8 nt. For visualization annotated duplicated cassettes of selected miRNAs are shown. (0.57 MB TIF) [file pone.0004456.s009.tif]

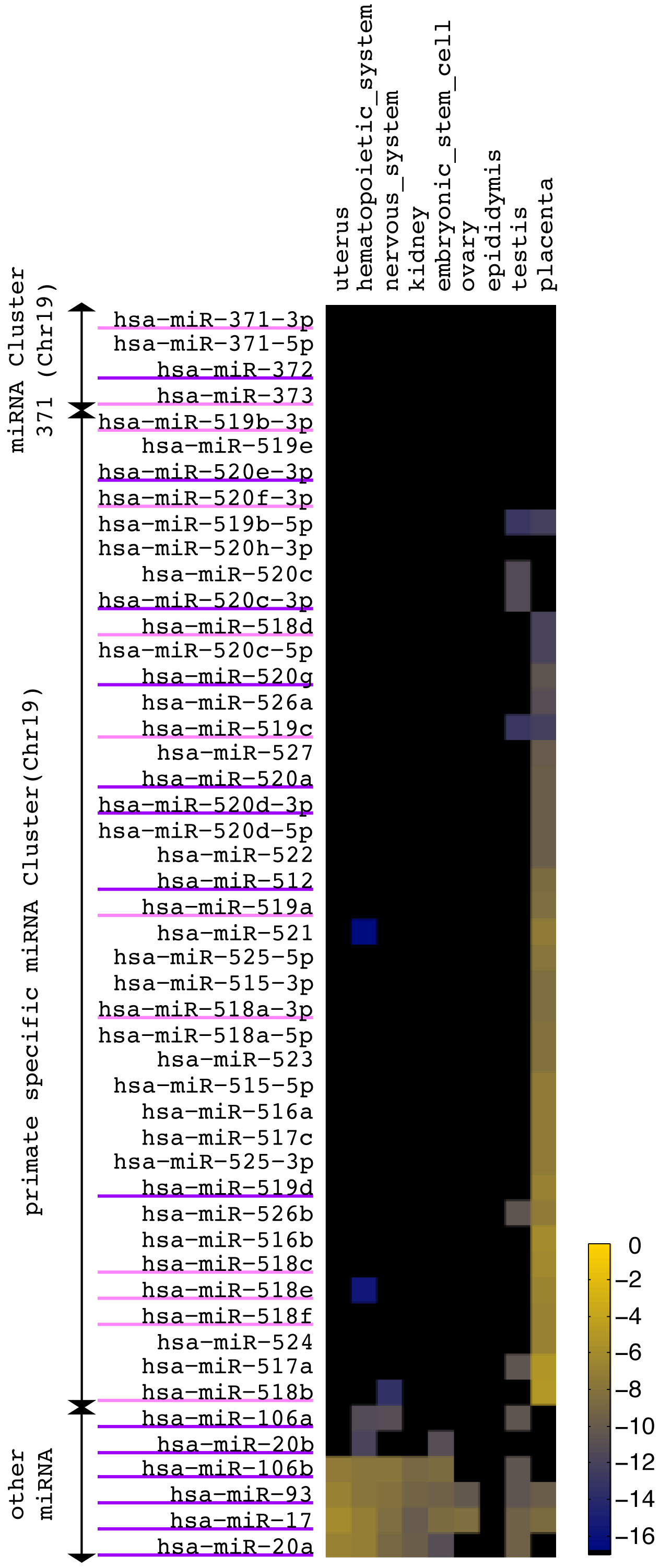

Supplement: Figure S10 — Human expression profile of Alu-targeting miRNA genes. Figure modified from [1]. Depicted miRNAs correspond to the miRNAs targeting sense Alu most often recognized and evolutionary most conserved target site (nucleotide 34–37, Figure 2, yellow marked). In addition, all members of the primate specific miRNA cluster on Chr19 C19MC are listed, as we proposed common driving force for gaining these miRNAs. Further the miRNA family members of miRNA cluster 371 are shown for two reasons. First as this cluster is positioned downstream of C19MC showing that some miRNAs of Chr19 are not highly expressed in placenta; second it was proposed that C19MC originated from miRNAs of this cluster [2]. An almost exclusive expression of C19MC miRNAs is seen in the placenta followed by the expression of a minor number of C19MC miRNAs in testis. In contrast miRNAs of Cluster 371 are not detectable in these two tissues. Two of the more ubiquitously expressed Alu targeting miRNAs (miR-93 and miR-17 of “other miRNAs”) are also detectable in placenta. As these are highly expressed in the hematopoietic system it is plausible that the signals detected in placenta origin from blood cell contamination. MiRNAs having an 8 nt seed match to the major conserved spot on sense Alu sequence are underlined (perfect seed match in purple, seed match with one mismatch in pink). 1. Landgraf P, Rusu M, Sheridan R, Sewer A, Iovino N, et al. (2007) A mammalian microRNA expression atlas based on small RNA library sequencing. Cell 129: 1401–1414. 2. Zhang R, Wang YQ, Su B (2008) Molecular evolution of a primate-specific microRNA family. Mol Biol Evol. (0.66 MB TIF) [file pone.0004456.s010.tif]
