# Supplementary material for: Evidence for Co-Evolution between Human MicroRNAs and Alu-Repeats
Source: PLoS One. 2009 Feb 11;4(2):e4456. doi: 10.1371/journal.pone.0004456 (PMC2637760; doi:10.1371/journal.pone.0004456)
Supplement: Figure S8 — Alignment of C19MC miRNA duplication cassettes. Each sequence consists out of one miRNA precursor with additional sequence of 1300 nt upstream and 700 nt downstream. The common core duplicated cassette is starting from alignment position (ap) ∼1290 onwards where a Alu fragment of ∼250 nt length is found. The next block of high conservation contains ∼80 nt short remains of a L1 element (ap ∼1770). This block is followed by a block containing a ∼370 nt sequence which resembles a kind of miRNA promoter sequence (starting at ap ∼2520) and the ∼85 nt precursor miRNA (starting at ap ∼2895). The cassette ends with two Alu element fragments of ∼80 nt and ∼150 nt length starting at ap ∼3200 and ∼3370. (1.51 MB PDF) [file pone.0004456.s008.pdf]

```
                        1
hsa-mir-519b_13/7       ----------------------------------------------------------------------------------------------------------------------------------
hsa-mir-523_13/7        ----------------------------------------------------------------------------------------------------------------------------------
hsa-mir-518a-2_13/7     ----------------------------------------------------------------------------------------------------------------------------------
hsa-mir-519c_13/7       ----------------------------------------------------------------------------------------------------------------------------------
hsa-mir-525_13/7        ----------------------------------------------------------------------------------------------------------------------------------
hsa-mir-518b_13/7       ----------------------------------------------------------------------------------------------------------------------------------
hsa-mir-520a_13/7       ----------------------------------------------------------------------------------------------------------------------------------
hsa-mir-518f_13/7       ----------------------------------------------------------------------------------------------------------------------------------
hsa-mir-522_13/7        ----------------------------------------------------------------------------------------------------------------------------------
hsa-mir-526a-1_13/7     ----------------------------------------------------------------------------------------------------------------------------------
hsa-mir-518e_13/7       ----------------------------------------------------------------------------------------------------------------------------------
hsa-mir-516a-1_13/7     ----------------------------------------------------------------------------------------------------------------------------------
hsa-mir-527_13/7        ----------------------------------------------------------------------------------------------------------------------------------
hsa-mir-526a-2_13/7     ----------------------------------------------------------------------------------------------------------------------------------
hsa-mir-518d_13/7       ----------------------------------------------------------------------------------------------------------------------------------
hsa-mir-521-2_13/7      ----------------------------------------------------------------------------------------------------------------------------------
hsa-mir-516b-2_13/7     ----------------------------------------------------------------------------------------------------------------------------------
hsa-mir-519a-1_13/7     ----------------------------------------------------------------------------------------------------------------------------------
hsa-mir-520c_58902519   ----------------------------------------------------------------------------------------------------------------------------------
hsa-mir-518a-1_13/7     ----------------------------------------------------------------------------------------------------------------------------------
hsa-mir-521-1_13/7      ----------------------------------------------------------------------------------------------------------------------------------
hsa-mir-519a-2_13/7     ----------------------------------------------------------------------------------------------------------------------------------
hsa-mir-520b_13/7       ----------------------------------------------------------------------------------------------------------------------------------
hsa-mir-519e_13/7       ------------------------------------------------------------------------------------------------TTTTTATTTTTATTTTTTTTTGAGATGGAGTCTT
hsa-mir-518c_13/7       ----------------------------------------------------------------------------------------------------------------------------------
hsa-mir-520g_13/7       ----------------------------------------------------------------------------------------------------------------------------------
hsa-mir-519d_13/7       ----------------------------------------------------------------------------------------------------------------------------------
hsa-mir-520h_13/7       ----------------------------------------------------------------------------------------------------------------------------------
hsa-mir-516b-1_13/7     ----------------------------------------------------------------------------------------------------------------------------------
hsa-mir-526b_13/7       ----------------------------------------------------------------------------------------------------------------------------------
hsa-mir-520d_13/7       ----------------------------------------------------------------------------------------------------------------------------------
hsa-mir-516a-2_13/7     ----------------------------------------------------------------------------------------------------------------------------------
hsa-mir-524_13/7        ------------------------------------------------------------------------------------------------TTCTTGTCAGCCTCGCAAGTAGTTGGGCTCAGGT
hsa-mir-520f_13/7       ----------------------------------------------------------------------------------------------------------------------------------
hsa-mir-520e_13/7       ----------------------------------------------------------------------------------------------------------------------------------
hsa-mir-517a_13/7       ----------------------------------------------------------------------------------------------------------------------------------
hsa-mir-517b_13/7       -----------GGCTAACTTTTGTATTTTTACAGATGAGGTTTTACCATGTTTCCCAGGCTGGTGTCTAACTCCTGAGAATATAAGGTATATGTAGTAGGCGTTGCTTTTTCTCTTTGAAGACAAAACCC
hsa-mir-517c_13/7       CCTCTGACTCTTTCTGGGATTGCAGGTATGCGCCCCCACGCCTGGCCTAATTTTTGAATGTTTAGCAGAGATGGGGTTTCACCATGTTGGCCAGGCTGAACTC-AGCTCCTGACCTTAGGTGATCTGCCT

                      131
hsa-mir-519b_13/7       ------------------------------------------------------------------CACTTGTACTTGTGTATTTCTGCTCAAATTTTATTGAGTAGTTTTTACATCCTAGCCTGTGATC
hsa-mir-523_13/7        --------------------------------------------------------------------------------------------------TTTCAGCACGTTCCACAGGCTGGACTCTTAAC
hsa-mir-518a-2_13/7     -------------------------------------------------------------------------------------------------------------------------CTCCTGGGG
hsa-mir-519c_13/7       ----------------------------------------------------------------------------------------------------------------------------------
hsa-mir-525_13/7        ----------------------------------------------------------------------------------------------------------------------------------
hsa-mir-518b_13/7       ----------------------------------------------------------------------------------------------------------------------------------
hsa-mir-520a_13/7       ----------------------------------------------------------------------------------------------------------------------------------
hsa-mir-518f_13/7       ----------------------------------------------------------------------------------------------------------------------------------
hsa-mir-522_13/7        ----------------------------------------------------------------------------------------------------------------------------------
hsa-mir-526a-1_13/7     ----------------------------------------------------------------------------------------------------------------------------------
hsa-mir-518e_13/7       ------------------------------------------------------------------------------------------------------------ACATAGAGAGCTTT---CTTTT
hsa-mir-516a-1_13/7     -----------------------------------------------------------------------GCTCCGCCTCCCCAGTTCATGATATTTTTCTGCCTCAGCCTCCCAAGTAGCTGGCACTA
hsa-mir-527_13/7        ---------------------------------------------------------------------------------------TCACGCCATTCTCCTGCCTCAGCCTCCTGTGTAGCTGGGACTA
hsa-mir-526a-2_13/7     ----------------------------------------------------------------AGACAGGGTGTCTGTAGCCCAGGCTCGAGTGCGGTGGCACTTCCAACCTAGATCTCTTGGGCTCAA
hsa-mir-518d_13/7       --------------------------------------------------------------------------------------------------------------------------CACCACAG
hsa-mir-521-2_13/7      ------------------------------------------------------------------------------------------GAATTTTTCTTCCTGTGAGCATACAATATATGGAACTTTC
hsa-mir-516b-2_13/7     -----------------------------------------------------------------------------------------------------------AGTATGCGATGTATGGAACTTTC
hsa-mir-519a-1_13/7     ----------------------------------------------------------------------------------------------------------------------------------
hsa-mir-520c_58902519   ----------------------------------------------------------------------------------------------------------------------------------
hsa-mir-518a-1_13/7     ----------------------------------------------------------------------------------------------------------------------------------
hsa-mir-521-1_13/7      ----------------------------------------------------------------------------------------------------------------------------------
hsa-mir-519a-2_13/7     ----------------------------------------------------------------------------------------------------------------------------------
hsa-mir-520b_13/7       ----------------------------------------------------------------------------------------------------------------------------------
hsa-mir-519e_13/7       ACTCTTGCCAGTCTGGAGTGCAGTGGCACGATCTCGGCTCACTGCAACTTCTGCCTCCTGGTTCAAGTAATTCCCCTGCCTCAGCCTCCCGAGTAGCTGGGACTACAGGTACATGCCACCACAGGCGGCA
hsa-mir-518c_13/7       ----------------------------------------------------------------------------------------------------------------------------------
hsa-mir-520g_13/7       ----------------------------------------------------------------------------------------------------------------------------------
hsa-mir-519d_13/7       --------------------------------------------------------------------------------CACTGGGGAAATGAAACCAACATCTTGGGTGCATGACCAGGTCATATATG
hsa-mir-520h_13/7       ----------------------------------------------------------------------------------------------------------------------------------
hsa-mir-516b-1_13/7     ----------------------------------------------------------------------------------------------------------------------------------
hsa-mir-526b_13/7       ----------------------------------------------------------------------------------------------------------------------------------
hsa-mir-520d_13/7       ----------------------------------------------------------------------------------------------------------------------------------
hsa-mir-516a-2_13/7     ----------------------------------------------------------------------------------------------------------------------------------
hsa-mir-524_13/7        TTTAATTTTTGGTAGAGATGGGGTCTCTTTTGCCTGGGATGGTCTCAGCTGAGCTTAAGTGATCCTAAATGTGTGAGCCACCTTGTCCCATTTCAAAGATAGGTGACACGGCCAGGCATCCTGGGACACA
hsa-mir-520f_13/7       ----------------------------------------------------------------------------------------------------------------------------------
hsa-mir-520e_13/7       ----------------------------------------------------------------------------------------------------------------------------------
hsa-mir-517a_13/7       ----------------------------------------------------------------------------------------------------------------------------------
hsa-mir-517b_13/7       AGGACAGTTGTCCCTCGATGAACAAGGCTAACCTGCTGAACGTTTGAAGCAAGGAACTGGAGATGGTCTTTGTATCGGTTTATGTTCTAGATTCCAGAAAATATGCAAACAGGACCAATAAATGCATCTT
hsa-mir-517c_13/7       GCCTCGGCCTCCCAAAGTGCTGAGAATACAGGCGTGAGCCACCGCGCCTGGCCATCAACACCTTTTACCCCACAGAAATTATTCTGGCACTGGTTTATGGAACTTCACTTGGGGTCAGGTAGAGGTGAAG

                      261
hsa-mir-519b_13/7       TACTCTGGA-GA---GTGTTTGTGCATATGA-CAGTGTATATTCTTCAACATCGTAGACTCCATTTTGGATGCTCCCTTCGTGACTGTGGGTCCTGTACTGGAACTCGAGTGAACACTTGG---CTTAAA
hsa-mir-523_13/7        TCCTGCGATCAA---GTGATCCTCCCGCTGTTCAAATTTTACTGAGGAGCTTTTAAGGCCTCGCCT---GTGGTCTGTCCTGTAGTGT---CCCTGTCCTGGAACTCAAGTGAATACTTGG---TTTAAA
hsa-mir-518a-2_13/7    TACTG-GAAGGA---GTGA--AGGCCTCTGGACTGGGAACACCAGGGCATTGCACCGGTGCAGGCA-GGATGAGCCAAGGGGAACGGAGAGCCAGGCATCGCTAA-CTGGCGACAATTTGG---------
hsa-mir-519c_13/7       -------------------------------------------------------------------GGGTTCTGTTTTTGTTTAGTTGT---TTTGCATCATGGGGTCTCTGTTGCCCAG---GCTGGA
hsa-mir-525_13/7        -------------------------------------------------------------------CTGTTCTTTTTTTCTGTCTTTGACTATTTGAGACAGTCTTGCTCTGTCACCCGA---GATGGA
hsa-mir-518b_13/7       --------------------------------------------------------------------------------------------------------------------------------GA
hsa-mir-520a_13/7       ----------------------------------------------------------------------------------------------------------TTGAGGTAGAGTTGA---AGGGAA
hsa-mir-518f_13/7       ---------------------------------------------------------------------------------CCACCTCCACCTCCCAGTTTCAAGCCGTT----CTCCTGC---CTCAGA
hsa-mir-522_13/7        -------------------------------------------------------------------------------------TGTTATTTCAATTTTTTTATGCGTA----TTGCCAA---AGCAAG
hsa-mir-526a-1_13/7     -------------------------------------------------------------------------------------------------------AATAAT-------GTGAT---CTCGGC
hsa-mir-518e_13/7       TTTGACACCGGGTCTCAAAAATATATCATATACATTTTATTATAATGTCTGATATTGA---TAATATAATCATACACACACAAAATACTCTTCTGTACACAGAGAATA----C---TGAT---ATCACT
hsa-mir-516a-1_13/7     CAGGC-ACCCGCCACCACGCCTGGCTAATTTT---TTGTATTTTTAGTGAAGACGGGGTTTCACTGGATTAG-CCAGGATGGTCTTGATCTCCTGAGCTC--GTGATC-----TGCCTGC---CTTGGC
hsa-mir-527_13/7       CAGGT-GCCCGCCAGCACACCCGGCTAATTCT----TTGTATTTTTAGTAGAGACGGGGTTTCACCGTGTTAG-CCAGGATGGTCTCGATCTCCTGACCTC--GTGATC-----CACCCGC---CTCAGC
hsa-mir-526a-2_13/7     CAAGTAGCCCTCTTTTTCGGACAGAG--TCTCGCGCT-GTGACCCTAGCTGGAGTGCAGTGGCAGGAACTTGGCTCACTGCCACCTCTGCCTCCTGGGTTCAAGCAATT-----CTCCTGC---CTCAGC
hsa-mir-518d_13/7       TGGGCTACCTTTTTTTTTTGAGAGGGAATTTGCTCTTGTTGCACAGGCTGGAGTACAATGGCAAGATTTCAGATCACTGCAACCTCCGCCTTCCGGGTTCATGTGATT-----CTCCTGC---CTCAGC
hsa-mir-521-2_13/7      TTTGACACAGGGTCTCAGAAATATATGGTATATATTTCGTTATATATATACTTATATA---TAATATAATCATATACACACAAAATACTGTTCTAAATACAGAGAATA----CT-CTGAT---ATAACC
hsa-mir-516b-2_13/7     CTTGAGACAGGGTCTCAAAAATATATCATGTATATTTTAT-ATATTCTTA-TCATATA---TAATATAATCATGTACACACAAAATACTCTTCTAAATACAGAGAATA----CT-CTGAT---ATCACC
hsa-mir-519a-1_13/7     --------------------------------------------------------------------------------------TGGATTCCAGAAAACATGCAAACAGGACCAATAAA---TGCGTG
hsa-mir-520c_58902519   ------------------------------------------------------------------------------------------------------TGCAAACAGGGCCAATAAA---TGCATC
hsa-mir-518a-1_13/7     -----------------------------------------------------------------TCCTTTTAGTGGTTTATGTTCTGGATTCCAGAAAACATGCAAACAGGGCCAATACA---TGCATC
hsa-mir-521-1_13/7      ---------------------------------------------------------------------------------------------------GCCCGCCACCAAGCCCGGCTAA---TTTTTT
hsa-mir-519a-2_13/7     -----------------------------------------------------------------------------------------------------------------CCAATAAA---TGCATC
hsa-mir-520b_13/7       ----------------------------------------------------------------------------------------------------------------GCCAATAAA---TGCATC
hsa-mir-519e_13/7       AATGGTTGTATTTTTAGTAGAGACGGAGTTTCACCATGTTGGCCAGGATGGTCGTGGTCTCTTGACCAGCCTCCCAAAGTGTTGGGATTCCAGGCGTGAGCCACCGCGCCTGGCCAATGAA---TGCATC
hsa-mir-518c_13/7       ----------------------------------------------------------------------------------------------------------------------------------
hsa-mir-520g_13/7       -------------GCGAGGCGTGGTGGTGGGTGCCTGTAGTCCCAGCTACCCGGGAGGCTAAGGCTTGAGAATGGCTTGAACCCAGGAGGCGGAGCTTGCAGTGAGCCGAGATCGTGCCAT---TGCACT
hsa-mir-519d_13/7       CAGTCATATATGCAATATGCATGGTGGTGGGTGCCTCTAATCCCAGCTACTCAGGAGGCTGACGCAGAAGAATCGCTTGAACCCAGGAGGCAGAAGTTGCCATGAGCCGAGATCCCACCAC---CGCA-T
hsa-mir-520h_13/7       ------------------------------------------------------------------------------------------------------------------ACACCAT---TGCACT
hsa-mir-516b-1_13/7     ----------------------------------------------------------------------------------------------------GGTTTCACCATGTTGTCCAGAGAATACAAG
hsa-mir-526b_13/7       --------------------------------------------------------------------------------CTGAGGTCAGTTGTTCGAGACCAGTCTGGCCAGCATGGTGAAACCCCGTC
hsa-mir-520d_13/7       -----------------------------------------------------------------------------------AGGTCGGGAGTTCAAGACCAGCCTAACCAACACGTAGAAACCCCATC
hsa-mir-516a-2_13/7     ---------------------------------------------------------------------------------------------TTCAAGACTAACAGGGCTGAAATAGCAAAACCTCATC
hsa-mir-524_13/7        CAGGGTCCCAGTTACTGCAAAGGCTAGGGTGGGAGGGTCCTTTGATTTTAAGGCTATACCATGCACTGATCACACCTTTGAATAGCCACTGCACTCCGGCCTGGGCCACATAGCAAGATCCCATCTCTTT
hsa-mir-520f_13/7       ----------------------------------------------------------------------------------------------------------------------------------
hsa-mir-520e_13/7       ----------------------------------------------------------------------------------------------------------------------------------
hsa-mir-517a_13/7       ----------------------------------------------------------------------------------------------------------------------------------
hsa-mir-517b_13/7       TATTTTTGTGTCCATTTTGACCAGGTCAAGGAAAATTTCAACAAGAAACCCAGAGTGCCGGAGCAAGAAGATCTCAAGCTGTGAGTCTACAAAGGGAAGCCCTTTCTGTTGTCTAAAAGAAAAGAAAGTG
hsa-mir-517c_13/7       GGGACCCCAATGTCCCTTGCAGATGGGATGTGTACTGCTCAGCAAGAGCATGGAGGTGGAGTGCATGGGGTTTGAGTTTTCATTGGGGAAATGAAGCTGAAATCTTGGGTGCATGACCAGGTCGTATATG
```

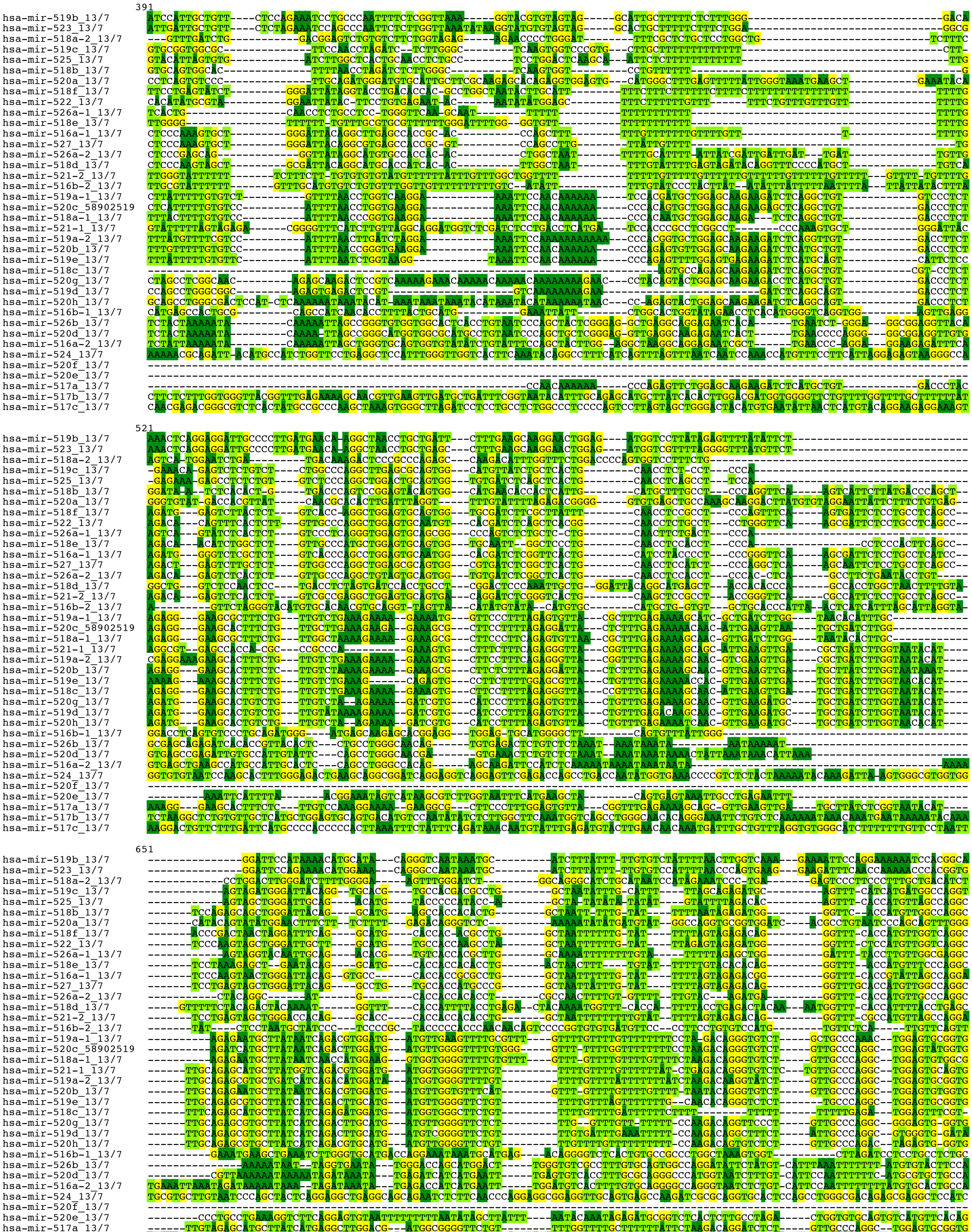
391
hsa-mir-519b_13/7
hsa-mir-523_13/7
hsa-mir-518a-2_13/7
hsa-mir-519c_13/7
hsa-mir-525_13/7
hsa-mir-518b_13/7
hsa-mir-520a_13/7
hsa-mir-518f_13/7
hsa-mir-522_13/7
hsa-mir-526a-1_13/7
hsa-mir-518e_13/7
hsa-mir-516a-1_13/7
hsa-mir-527_13/7
hsa-mir-526a-2_13/7
hsa-mir-518d_13/7
hsa-mir-521-2_13/7
hsa-mir-516b-2_13/7
hsa-mir-519a-1_13/7
hsa-mir-520c_58902519
hsa-mir-518a-1_13/7
hsa-mir-521-1_13/7
hsa-mir-519a-2_13/7
hsa-mir-520b_13/7
hsa-mir-519e_13/7
hsa-mir-518c_13/7
hsa-mir-520g_13/7
hsa-mir-519d_13/7
hsa-mir-520h_13/7
hsa-mir-516b-1_13/7
hsa-mir-526b_13/7
hsa-mir-520d_13/7
hsa-mir-516a-2_13/7
hsa-mir-524_13/7
hsa-mir-520f_13/7
hsa-mir-520e_13/7
hsa-mir-517a_13/7
hsa-mir-517b_13/7
hsa-mir-517c_13/7
521
651

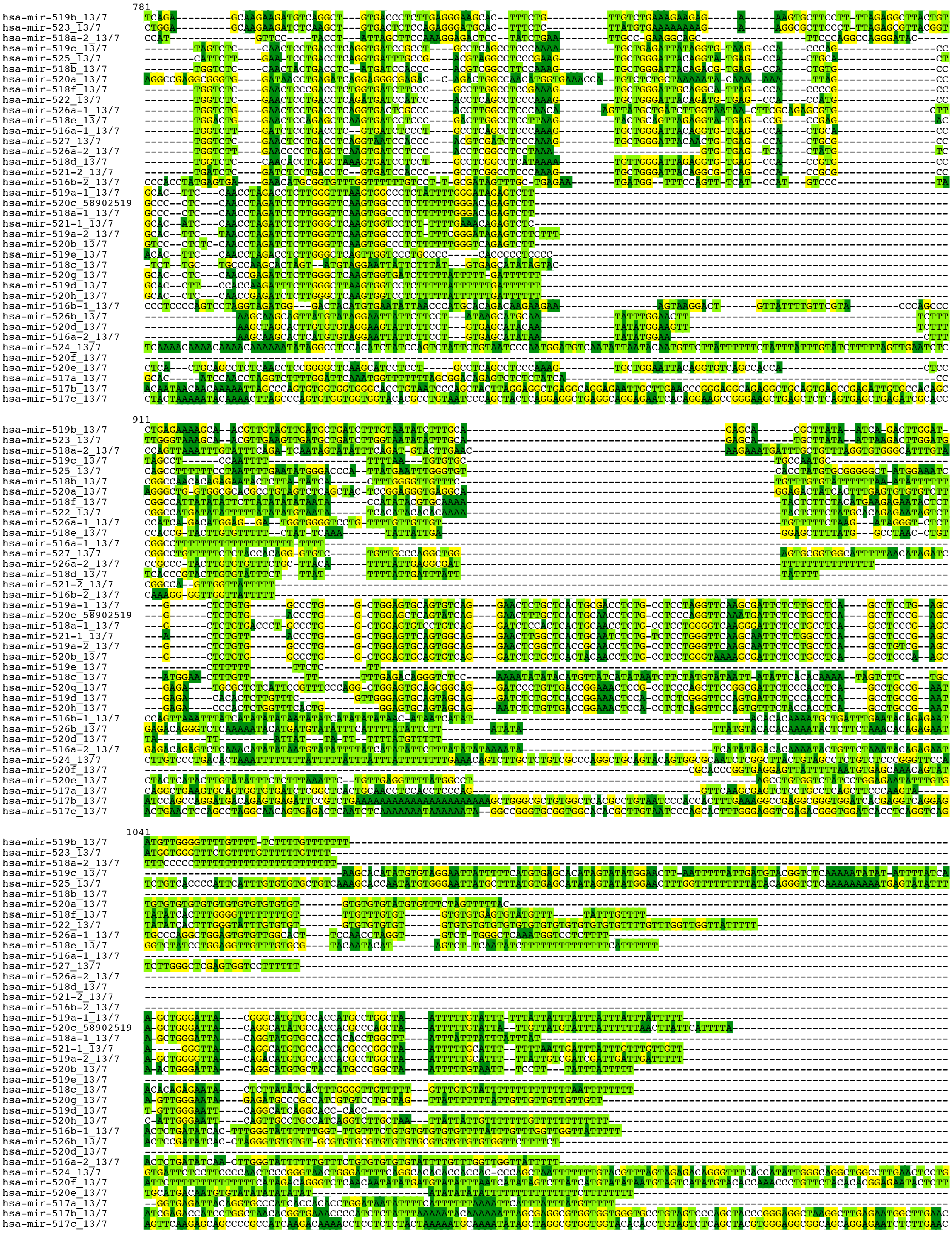
781
911
1041
hsa-mir-519b_13/7
hsa-mir-523_13/7
hsa-mir-518a-2_13/7
hsa-mir-519c_13/7
hsa-mir-525_13/7
hsa-mir-518b_13/7
hsa-mir-520a_13/7
hsa-mir-518f_13/7
hsa-mir-522_13/7
hsa-mir-526a-1_13/7
hsa-mir-518e_13/7
hsa-mir-516a-1_13/7
hsa-mir-527_13/7
hsa-mir-526a-2_13/7
hsa-mir-518d_13/7
hsa-mir-521-2_13/7
hsa-mir-516b-2_13/7
hsa-mir-519a-1_13/7
hsa-mir-520c_58902519
hsa-mir-518a-1_13/7
hsa-mir-521-1_13/7
hsa-mir-519a-2_13/7
hsa-mir-520b_13/7
hsa-mir-519e_13/7
hsa-mir-518c_13/7
hsa-mir-520g_13/7
hsa-mir-519d_13/7
hsa-mir-520h_13/7
hsa-mir-516b-1_13/7
hsa-mir-526b_13/7
hsa-mir-520d_13/7
hsa-mir-516a-2_13/7
hsa-mir-524_13/7
hsa-mir-520f_13/7
hsa-mir-520e_13/7
hsa-mir-517a_13/7
hsa-mir-517b_13/7
hsa-mir-517c_13/7

```
                   1171
hsa-mir-519b_13/7     -------------------------------------------------------------------------------------------------------------------------ATCTAAGAC
hsa-mir-523_13/7      -------------------------------------------------------------------------------------------------------------------------AAATCAGAG
hsa-mir-518a-2_13/7   -------------------------------------------------------------------------------------------------------------------------TTGACAGAG
hsa-mir-519c_13/7     TATTCTTATTATACATGTAACATAGTCATATACACACAAAACACTCTTCTATACACAGAGAATACTCTCGTATCACTTTGGGTAATTTTTTGTTTGTGTGTGTATGTTTTTGTTTTTTTTTTAGATAAGG
hsa-mir-525_13/7      CATCACAATGTTCTGTGTA---TAATTCCATACACACAAAATAGTCTTC-ATACACAGAGAACACTCTTATATCACTTTGGAGTTGTTTTTGTTTGTGTATTT-TGTTTAATTTTTAT---GAGACAGGG
hsa-mir-518b_13/7     -------------------------------------------------------------------------------------------------------------------------GAGACACAG
hsa-mir-520a_13/7     -------------------------------------------------------------------------------------------------------------------------AAGACACAG
hsa-mir-518f_13/7     -------------------------------------------------------------------------------------------------------------------------TGGATAAGG
hsa-mir-522_13/7      -------------------------------------------------------------------------------------------------------------------------GAGACAAGG
hsa-mir-526a-1_13/7   -------------------------------------------------------------------------------------------------------------------------TTCGGACAG
hsa-mir-518e_13/7     -------------------------------------------------------------------------------------------------------------------------GAGAGGGAG
hsa-mir-516a-1_13/7   -------------------------------------------------------------------------------------------------------------------------GAGATGGGA
hsa-mir-527_13/7      -------------------------------------------------------------------------------------------------------------------------TTGGTTCAG
hsa-mir-526a-2_13/7   -------------------------------------------------------------------------------------------------------------------------GAGACGGAG
hsa-mir-518d_13/7     -------------------------------------------------------------------------------------------------------------------------GAGACAGAG
hsa-mir-521-2_13/7    -------------------------------------------------------------------------------------------------------------------------GAGACAAGG
hsa-mir-516b-2_13/7   -------------------------------------------------------------------------------------------------------------------------GAGACAAGG
hsa-mir-519a-1_13/7   -------------------------------------------------------------------------------------------------------------------------GAGACAGAG
hsa-mir-520c_58902519 -------------------------------------------------------------------------------------------------------------------------GAGACAGAG
hsa-mir-518a-1_13/7   -------------------------------------------------------------------------------------------------------------------------GAGACAGAG
hsa-mir-521-1_13/7    -------------------------------------------------------------------------------------------------------------------------GAGACAG--
hsa-mir-519a-2_13/7   -------------------------------------------------------------------------------------------------------------------------GAGACAGAG
hsa-mir-520b_13/7     -------------------------------------------------------------------------------------------------------------------------GAGACAGAG
hsa-mir-519e_13/7     -------------------------------------------------------------------------------------------------------------------------GAGACAG--
hsa-mir-518c_13/7     -------------------------------------------------------------------------------------------------------------------------GAGACACCA
hsa-mir-520g_13/7     -------------------------------------------------------------------------------------------------------------------------GAGATGGAG
hsa-mir-519d_13/7     ----------------------------------------------------------------------------------------------------------------------------ATCGTG
hsa-mir-520h_13/7     -------------------------------------------------------------------------------------------------------------------------GAGGCGGAG
hsa-mir-516b-1_13/7   -------------------------------------------------------------------------------------------------------------------------GAGACAAGG
hsa-mir-526b_13/7     -------------------------------------------------------------------------------------------------------------------------GAGACAATA
hsa-mir-520d_13/7     -------------------------------------------------------------------------------------------------------------------------GCGACTGAG
hsa-mir-516a-2_13/7   -------------------------------------------------------------------------------------------------------------------------GAGACAAGG
hsa-mir-524_13/7      AACTAAGGTGATCCGC-CCACCTCGGCCTCCCAAAGTGCTGGGATTAGGGAT------------------------------------------------------------------------------
hsa-mir-520f_13/7     GTATCACTTTGGGTATTTAT----------------------------------------------------TTACTTGTTTCTTTCTGTGTTTTTCTTGTTTGTTTGTTTGTTTTGTTTTGAGACAAGG
hsa-mir-520e_13/7     -------------------------------------------------------------------------------------------------------------------------GAGATGGAG
hsa-mir-517a_13/7     -------------------------------------------------------------------------------------------------------------------------GAGATGGAG
hsa-mir-517b_13/7     CCAGGAGGCGGAGCTTGCAGTGAGCCGAGATCGTGCCATTGCACTCTAGCCTCGGCAACAGAGCAAGACTCCGTCAAAAAGAAACAAAAACAAAAACAAAAAAAAGA-----------------------
hsa-mir-517c_13/7     CCGTGAGAAGGAGATTGCAGTGAGCTAAAATCACACCATTGCACTGCAGCCTGGGCGACTCCATCTCAAAAAATAAATACATAAATAAATAAATACATAAATACATAAAAAATA----------------

                   1301
hsa-mir-519b_13/7     TGGGTCTCT-GTTGCCCAGGCTGAAGTGCAGTGGCACAATCTTGGCTCACCACAACCTCCGCCTCCCGGGTTCAAGTGATTCTTCTGCCTCAGCCTCCCAAGTAGCTGGGATTATAGGACTGCGCCACCA
hsa-mir-523_13/7      TCTCACTCT-GTCACCCAGGCTGGAGTGCAATGGCGCAATCTCGGCTCACTGCAACTTCCCCCTCCTGGGTTCAAGTGATTCTCCTGCCTCAGCCTCCTGAGTAGTTAGGATTACAGACATGCGCCACCT
hsa-mir-518a-2_13/7   TTTTGCTCTTCTTACCCAGGCTGGAATGCAATGGCACAATCTTGACTCACTGCAACCTCCACCTCCCAGGTTCAAGCGATTCTCCTGCCTCAGCAGCCCGAGTAGCTGGGATTACAGGCATGCGCCACCA
hsa-mir-519c_13/7     TCTGGGTCT-GTCACCCAGGCTAGAGTGCAGTGGTGCCATCTCAGCTTATTGCAACCTCCACCTGCCA-------------------CTTCAGCCTCTTGAGTAGCTGT--CTACAGGCGCACACCACCA
hsa-mir-525_13/7      CCTGGCTAT-GTCGCTGAGGCTGGAGGGCAGTGGTGAGATCTTGGCTCACTGCAACCTCCACCTGCCA-------------------CCTCAGCCTCCTGGATTAGTGT--CTACAGGCAGGCACCACCA
hsa-mir-518b_13/7     TCTGGCGCT-ATTACCTAGGTTGGAGTGCAGTGTTGTGACCTCGGCTCACAGCAACCTCTACCTCCCAG-------------------CTGAGCCTCCTGAGTTAGCAT--CTGCAGGCATGAACCACAA
hsa-mir-520a_13/7     CCTGACTAT-ATCACCGAGGCTGGAGTGCAGTGGTGCAATGTTGGCTCACTGCAACCTCCACCTGCCA-------------------CCGCAGCCTCCTGAATTAGTGT--CTACAGGTATGCACCACCA
hsa-mir-518f_13/7     TCTGGCTCT-GTCACCCAGTCTAGAGTGCAGTTGTGCCATCTCAGCTCACTGCGACTTCTACCTCCCA-------------------CCTAAGCCTGCTGAATTAGTGT--CTACAGGCATGCACCACCA
hsa-mir-522_13/7      TCTGGCTCT-GTCATCCAGGCTGGAGTGTAGTGGTGTGATCTCAGCTCACTGCAACCTCCACCCCCGA-------------------CCTAAGCCTACTGAATTAGTGT--CTACAGGCACGCACTACCA
hsa-mir-526a-1_13/7   TCTTGCTCT-GTGACCCATGCTGGAGTGTAGTGACGTGATCTCAGCTCACTGCAACCTCCACCTCCCAGTTTTAGGCAGTTCTCCTGCCTCACATTCCTGAGTAGCTGGGATTATAGGTGCCTGACACCA
hsa-mir-518e_13/7     TTTCGCTCCTGTCACCTAGGCTGGAGTGCAATGACATGATCTCAGTTCACTGCAACCTCCGCCTCCTGGGTTCAAGTGATTCTCCTGCCTCAGCCTCCTGAGTAGCTGGGATTACAGGCACCCGCCACCA
hsa-mir-516a-1_13/7   TCTCGCTCT-GTCACCCAGGCTGGAGTGCAACGGCATGATCTCAGCTCACGGCAACCTCCCCCTCCCGGGTTCAGGTGATTCTACTGCCTCAGCCTCCCAAGTAGCTGGGATTACAGGTGCCCACGACCA
hsa-mir-527_13/7      TCTTGCTTT-GTGACCCAGGCTGGAGGGCAGTGGCAGAAACTCTCCTCACTGCAACCTCCGCCTCCTAGGTTCAAGCCATTCTCCTGACTCAGCCTCCTGAGCAGCTGGGATTACCGGTGTGAGCCACCA
hsa-mir-526a-2_13/7   TCTTGGTCT-GTTACCTAGGCTGGAGCGCAGTGGCGCGATCTTGGCTCACTGCAAGCTCCGCCTCCCAGGTTCATGCCATTCTCCTGCCTCAGCCTCCTGAGTAGCTGGGACTACAGGCGCCCACCACCA
hsa-mir-518d_13/7     TTTCACTCTTGTTGCCCAAGCTGGAGTGCAATGGCACAATCTCGGCTCAATGCAACCTCCTTCTCTTAGGTTCAAGCGATTCTCCTGCCTCAGCTTCCCGGGTAGCTGGGATTAAAGGCGTCCACCACCA
hsa-mir-521-2_13/7    TCTGGCTCT-ATCACCCAGGCTGGAGTGTAGTGGTGCGATCTTGGCTCACTGCAACCTCCACCTCCCA-------------------CCTAAACCTGCTGAATTAGTGT--CTACAGGCATGCACCACCA
hsa-mir-516b-2_13/7   TCTGACTCT-ATCGCCCAGGCTGGAGTGTAGTGGTGCCATCTCGGCTCACTGCAACCTCCACCTCCCA-------------------CCTAATCCTGCTGAATTAGTGT--CTGCAGGCATGCACAACCA
hsa-mir-519a-1_13/7   TCTCGCTCT-GTCACCCAGGCTGAAGTGCAGTGGTGCGATCTTGGCTCACCGCAACCTCTACCTCCCA-------------------CCTCAGCCTCCTGAACAGCTGT--CTCCAGGCGTGCACCACCA
hsa-mir-520c_58902519 TCTCACTCT-GTCGCCCAGGCTGGAGTGCAGTGGTGCGATCTCGGCTCACTGCAACCTCCAACTCCCAG-------------------CTCAGCCTCCTGAATAGCTGT--CTGCAGGCATACACCACCA
hsa-mir-518a-1_13/7   TCTTGCTGT-GTCATCCATGCTGGAGAGCAGTGGTGCAATCTCGGCTCTCTGCAACCTCCACCTCCCA-------------------CCTCAGTCTCCTGAATTAGTAT--CTACAGGCATGTACCACCA
hsa-mir-521-1_13/7    TCTCACTCT-GTTGCCCAGGCTGTAGTGCAGTGGTGTGATCTCAGCTCACCGCAACCTCCACCTCCCA-------------------TGTCAGCCTCCTGAATACCTGT--CTCCAGGCATGCACCCCCA
hsa-mir-519a-2_13/7   TCTGGCTCT-GTCACCCAGGCTGTCGGGCAGTGGTGCGATCTTGGCTCACTGCAACCTCCACCTCCCA-------------------CCTCAGCCTCCTGAATACCTGT--CTACAGGCATGCACAACCA
hsa-mir-520b_13/7     TCTTACTCTT-TCGCCCAGGCTGGAGTGCAGTGGTGCAATCTCGGCTCACTGCAACCTCCAACTCCCA-------------------CCTCAGCCTCCTGAATAGCTGT--CTACAGGCATGCACCACCA
hsa-mir-519e_13/7     TCTCTCTCT-GTGGCCCAGGCTGGAGTGCAGTGGTATGTTCTCTGCTCACTGCAACCTCTGCCTCCCG-------------------------------AGTAGCTGGTATTACAGGCACATGCCACCA
hsa-mir-518c_13/7     TCTGGCTCT-ATTGCCCAGGCTGGAGTGCAGTGTTGTGACCTCAACTCACTGCAGCCTCTACCTCCCA-------------------CCTCAGCCTCCTGAATTAGTGT--CTACAGGCATGAACCACTA
hsa-mir-520g_13/7     TTTCACTCTTGATGTCCAGGCTGGAGTGCAGTGTTGCAATTTAGGCTCACTGCAACCTCCACCTCCTGGGTTCAAGCGATTCTCCTGCCGCAGCCTCCTGGGTAGCTGGGATTACAGGTGCCTACTGCCC
hsa-mir-519d_13/7     TCCCACTCTTGCTGCCCATCCTGGAGAGCACTGGCATGATCTAGGCTCACTGCAACCTCCGCCTCCTGGGTTCGAGTGATGCTTCTGCCTCAGCCTCCCAGGTAGTTGGGATTACAGGCACCTACTGCCC
hsa-mir-520h_13/7     TTTCGCTCTTGTTGCCCAGGCTGGAGGGCAATAGCGCGATCTCGGCTCACCGCAACCTCGGCCTCCCGGGTTCAAGTGATTCTCCTGCCTCAGCCTCCCAGATAGCTGGAATTACAGGTGCCTACCACCC
hsa-mir-516b-1_13/7   TCTGGCTCT-ATCACCCAGGCTGGAGTGTAGTGGTGCGTTCTCAGCTCACTGCAACCTCCGCCTTCCA-------------------CCTAAGCCTGCTGCATTAGTGT--CTACAGGCATGCACCACCA
hsa-mir-526b_13/7     TCTGCCTCT-GTCACCCAAGATGGAGTGTGGTGGTGCAATCTCGGCTTACTGCAACCTCCACCTCCCA-------------------CCTAAGCCTGCTGAATTAGTCT--CTACAGGCATGCACCACCA
hsa-mir-520d_13/7     TTTTGCTCTTGTCGCATAGGCTGAAGTGCAATGGTGTGATCTGGGCTCCCTGCGATGTCCACCTCTTGGGTTAAAGCGACTCTCCTACCTCAGCCTCCTGAGTAGCTGGGATTACAGGCATACACTTCCA
hsa-mir-516a-2_13/7   TCTAGCTCT-ATCACACAGGCTGGAGTGTAGTGGTGCGTTCTCGGCTCACTGCAACCTCCGCCTCCCA-------------------CCTAAGCCTGCTGCATTAGTGT--CTACAGGCATGCACCACCA
hsa-mir-524_13/7      ----------------------------------------------------------------------------------------------------------------------------------
hsa-mir-520f_13/7     TCTAGCTTT-ATCACCCAGGCTGGAGTGCAGTGGTGCCATGTCGACTTACTGCAACCTCCACCTGCCA-------------------CCTCAGCCTCCTGAGTACTTGT--CTACAGGTGCGCACCACCA
hsa-mir-520e_13/7     TTTCACTCTTGTTGCCCTAGCTGGAGTACAGTGGCATGATCTCAGCTCACTGCAACCTCCGCCTCCCGGGTTCAAGCGATTTTCCTGCCTCAGCCTCCCGAGTAGCCGGGATTAC------CCTCCACCA
hsa-mir-517a_13/7     TCTCACT---GTTACCCAGGCTGGAGGGCCGAGGCGTGATCTCTGCTCAGTGCAACCTCTGCCTCCCGGATTCAAGCAATTCTTCTACCTCAGCCTCCTGAGTAGCTAGAATTACGGGAGCCCACCACCA
hsa-mir-517b_13/7     ----------------------------------------------------------------------------------------------------------------------------------
hsa-mir-517c_13/7     ----------------------------------------------------------------------------------------------------------------------------------

                   1431
hsa-mir-519b_13/7     TGCCTGGCTAA-TTTTGTATTTTTAGTAGAGACAGGGTTTCTTTAGG-------AGCT------------------------------------------------------------------------
hsa-mir-523_13/7      CACCCGGCTCAGTTTT-TGTATTTGGTGGAGATGGGGTTTCACCATGTTAGTCAGGCTGGTCTTGAACTCCTGATCTCAGGTGATCCAC-----------------------------------------
hsa-mir-518a-2_13/7   CACCCGGCTCAGTTTT-TGCATTTAGTGGAGATGGGGGTTCACCATGTTAGCCAGGCTGGTCTCAAACTCCTGACCTCAAGTGATTTCC-----------------------------------------
hsa-mir-519c_13/7     CGCCCGGCTAACTTTTCTGTTTTCTCTACACATGAGGTATCACCATGTTGCCCAGGCTGGTCTCAAACTCTTGAACTCAAGTGATACTC-----------------------------------------
hsa-mir-525_13/7      CCCCTGGGTAAGTTCTGTATTTTTGGTACTGATGAGGTTTCAGCACGTTCCACAGGCTGGACTCTAACTCCTGCGATCAAGTGATCCTC-----------------------------------------
hsa-mir-518b_13/7     CACCTGG-TAACCTTTGTATTTTTGG-ACACATGAGGTTTCACCATGTTGCCCAGGCTAGTCTTGAACTCCTGAGCTCAAGTAATCCTC-----------------------------------------
hsa-mir-520a_13/7     CACCTGGCTCACTTTTGTATTTTTTGTACAGATGAAGCTTCACCATGTTGCCCAGGCTTGTCTGGAACTCTTGAGCTCAAGTGATCCTC-----------------------------------------
hsa-mir-518f_13/7     CACCTGGCTCCGTTTTGTATTTTTTATACACATGGGCTTACACCATGTTGCCGAGACTGGTCTCGAACACCTGAGCTCAGGTGATACTT-----------------------------------------
hsa-mir-522_13/7      CACCTGGCTCACTTTTGTATTTTTTGTACACATGGGGTTTCACAATGTTGCCGGGGCTGGTCTCAAACTCCTGTGCTCAGGTGATCCTC-----------------------------------------
hsa-mir-526a-1_13/7   TGCCTGGCTAATATTTGCATGTTATTTATTCAT-GTATTTATTTGTTTTGAGATGG--AGTCTCACTGTCCTGGCCAGAGGGCAGTGGCAGGACCTCGGCTCACTCAAACCTTTGCCTCTCAGGTTCAAG
hsa-mir-518e_13/7     TGCCTAGCTAAGTTTG-TATTTTCAGTAGAGACG-GGCTTCTCCATGTTGGTCAGGCTGGGCTCAGACTCCTGACCTCAGGTGATCCGC-----------------------------------------
hsa-mir-516a-1_13/7   CGCCTGGCTAATTTTTGTATTTTTAGTAGAGATGGGGTTTCATCAAGGTGGCCAGGCTGGTCTCAAACTCCTGACCTCA--TGATCTAC-----------------------------------------
hsa-mir-527_13/7      CACCCAGCTAATTTTTATTTTTATTGTTGAGACGGAGTCTTGCTGTGTCTCCCAGGCTGGAGTGCAGT------------------------GGCGCGATCGTGGCTTACTGCAACCTCCTCCTCC----
hsa-mir-526a-2_13/7   CGCCCGGCTAATTTTTGTATTTTTAGTAGAGATGGAGTTTCACCTTGTTAGCCAGGATGGTCTAGATCTCCTGACCTT--GTGATCCTC-----------------------------------------
hsa-mir-518d_13/7     CACCCAGCTAATTTTTGTGTTTTTAGTAGAGACAGGGTTTCAGCATGTTGGCCAGACTGGTCTCAAACTCCTAACCTCAGGTAATCCAC-----------------------------------------
hsa-mir-521-2_13/7    CACCTGGCTCACTTTTGTGTTTTTCATACACATGGGTTTACACCATGTTGCTGGGGCTGGTCTCGAACTCCTGAGCTCAGGTGATCCTC-----------------------------------------
hsa-mir-516b-2_13/7   CATCTGGCTCACTTTTGTATTTTTTATACACATGGGTTTACACCATGTTGCAGGCGCTGGTCTCTCCCTCCCAAGCTCAGGTGATCCTC-----------------------------------------
hsa-mir-519a-1_13/7   CACCTGGCTAACTTCTGTATTTGTTTTACAGTCATGGTTTCACCATGTTGCCCGGGCTCATCTTGAACTCCTGAGCTCAAGTGATCCTC-----------------------------------------
hsa-mir-520c_58902519 CACCTGGCTAACTTTTGGTTTTTTGTACAGATGTGGTTTCCCCATGTTGCCCAGGTTGGTCTCGAACTCCTTAGCTCAGGTGATCTTC-----------------------------------------
hsa-mir-518a-1_13/7   TACCTGGCTAACTTTTGTATTTTT---ACAGATGAGGTTTTACCATGTTTCCCGGGCTGGTGTCTAACTCCTGAGCTCAAGTGATCCTC-----------------------------------------
hsa-mir-521-1_13/7    CACCTGGCTAACTTTTGCATTTTTTGTACAGATGAGGTTTCACCATGTTGCCCAGGCTGGTCTTGAACCCCTGAGCTCAAGTGGTCCTC-----------------------------------------
hsa-mir-519a-2_13/7   CACCTTGCTAACTTTTGTATTTTTTGTACAGATGAGGTTTCACCATGTTGCCCAGGCTGGTCTCGGACCCCTGAGCTCAAGTACTCCTC-----------------------------------------
hsa-mir-520b_13/7     CACCTGGCTAACTTTTGTATTTTTTGTACAGACATGGTTTCTCCATGTTGCCTGGGCTGGTCTTGAACTCCTGAACTCAAGTGATCCTC-----------------------------------------
hsa-mir-519e_13/7     CGCCTGGCTAATTTTTGTATTTTTAGCAGAGACGGAGTTTCATCATGATGGCCAGATTAGTCTTGAACTCCTGACCTTAGGTGATCCAC-----------------------------------------
hsa-mir-518c_13/7     CACCTGGCTATCTTTTGTATTTTT---ACAGATGAGGTTTTACTATGTTGGGCAGGCTAGTTTTGAACTC--GAGCTCATGTAATTTTC-----------------------------------------
hsa-mir-520g_13/7     CACCCAGCTAATTTTTGTGTTTTGAATAGAGACTGGGTTTCACCATGTTGGGCAGGCTGGTCTCAAACTCCCGACCTCAGGTGATCCAC-----------------------------------------
hsa-mir-519d_13/7     CACCCAGCTAATTTTTATGTTTTTAGTAGAGAC-GGGTTTCACCATGTTGGCCAGGCTAGTCTTGAACTCCCGCTTTCAGGTG-----------------------------------------------
hsa-mir-520h_13/7     CACCCAGCTAATTTTTGTGTTTTCAGTAGAGATGGGGTTTCACCATGTTGGGCAGGCTGGTCTCAAACTCCTGACCTCAGGTGATCCAC-----------------------------------------
hsa-mir-516b-1_13/7   CACCCGGCTCACTTTTGTATTTTTTGTACACATGGGGTTACACCATGTTGCCGGGGCTGGTCTCGAACTCCTGACCTCAGGTGATTCTC-----------------------------------------
hsa-mir-526b_13/7     CACCTGGCTAACTTCTGTAATTTTTGTACACATGGGGTCTCGCCATGTTGCCGGGGCTGGTCTCGAGCTCCTGAGG-CAGGTGATCCTC-----------------------------------------
hsa-mir-520d_13/7     GGGCTAGCTAATTTGTGTATTTTA---GTAGACGGGATTTCTCCATGTTGGTCAGGCTGGTCTCAAACTCCCGACTTCAGGTGATCTGC-CCACCTTGGCTTTCCAAAGTACTGGGATTGCAGACAGCCA
hsa-mir-516a-2_13/7   CACCCGGCTCACTTTTGTATTTTTTGTACACATGGGGTTACACCATGTTGCCGGGGCTGGTCTCGAACTCCTGAGCTCAGGTGATTCTC-----------------------------------------
hsa-mir-524_13/7      ----------------------------------------------------------------------------------------------------------------------------------
hsa-mir-520f_13/7     CACCCAGCTAACTTTTCTATTTTTT-----------------------------------------------------------------GTGCATAAGGTTTCACCATATTACCCATGCTGGTTGAGCT
hsa-mir-520e_13/7     CACCCGGCTAATTTTTGTATTTTTAGTAGAGGTGGGGTTTCGCCATATTGACCAAGGTGGTCTTGAACTCCTGACCTCAGGAGTCCCGC-----------------------------------------
hsa-mir-517a_13/7     CACCTGGCTACTTTTTGTTTTTTGTGTAGAGATGGGATTTCACCATGTTTGCCAGGCTGGTCTCAAACCCCTGACCTCAAGTTAGCCAC-----------------------------------------
hsa-mir-517b_13/7     ----------------------------------------------------------------------------------------------------------------------------------
hsa-mir-517c_13/7     ----------------------------------------------------------------------------------------------------------------------------------
```

```
                       1561
hsa-mir-519b_13/7      ----------------------------------------------------------------------------------------------------------------------------------
hsa-mir-523_13/7       ----------------------------------------------------------------------------------------------------------------------------------
hsa-mir-518a-2_13/7    ----------------------------------------------------------------------------------------------------------------------------------
hsa-mir-519c_13/7      ----------------------------------------------------------------------------------------------------------------------------------
hsa-mir-525_13/7       ----------------------------------------------------------------------------------------------------------------------------------
hsa-mir-518b_13/7      ----------------------------------------------------------------------------------------------------------------------------------
hsa-mir-520a_13/7      ----------------------------------------------------------------------------------------------------------------------------------
hsa-mir-518f_13/7      ----------------------------------------------------------------------------------------------------------------------------------
hsa-mir-522_13/7       ----------------------------------------------------------------------------------------------------------------------------------
hsa-mir-526a-1_13/7    CAAGTCTTCTGCCTCAACCTCCTGAGTAGCTGGAATCACAGGCACCCGTCACCACGCCCGGTTACTTTTGTATTTTTTTTTTCACCATGTTGGCCAGGCTGGTCTGAAACCCCTGACTTCATGTGAGCCG
hsa-mir-518e_13/7      ----------------------------------------------------------------------------------------------------------------------------------
hsa-mir-516a-1_13/7    ----------------------------------------------------------------------------------------------------------------------------------
hsa-mir-527_13/7       --------------------------------------------------------------------------------------------------------------------TTGGTTCAACCAAT
hsa-mir-526a-2_13/7    ----------------------------------------------------------------------------------------------------------------------------------
hsa-mir-518d_13/7      ----------------------------------------------------------------------------------------------------------------------------------
hsa-mir-521-2_13/7     ----------------------------------------------------------------------------------------------------------------------------------
hsa-mir-516b-2_13/7    ----------------------------------------------------------------------------------------------------------------------------------
hsa-mir-519a-1_13/7    ----------------------------------------------------------------------------------------------------------------------------------
hsa-mir-520c_58902519  ----------------------------------------------------------------------------------------------------------------------------------
hsa-mir-518a-1_13/7    ----------------------------------------------------------------------------------------------------------------------------------
hsa-mir-521-1_13/7     ----------------------------------------------------------------------------------------------------------------------------------
hsa-mir-519a-2_13/7    ----------------------------------------------------------------------------------------------------------------------------------
hsa-mir-520b_13/7      ----------------------------------------------------------------------------------------------------------------------------------
hsa-mir-519e_13/7      ----------------------------------------------------------------------------------------------------------------------------------
hsa-mir-518c_13/7      ----------------------------------------------------------------------------------------------------------------------------------
hsa-mir-520g_13/7      ----------------------------------------------------------------------------------------------------------------------------------
hsa-mir-519d_13/7      ----------------------------------------------------------------------------------------------------------------------------------
hsa-mir-520h_13/7      ----------------------------------------------------------------------------------------------------------------------------------
hsa-mir-516b-1_13/7    ----------------------------------------------------------------------------------------------------------------------------------
hsa-mir-526b_13/7      ----------------------------------------------------------------------------------------------------------------------------------
hsa-mir-520d_13/7      CCGCGGCTGGCCTTCATATATTCTTATATATATAATATCATGTACACACAAAAGACTCTTTTACACCCTG-AGAATACTCTTATATCACTTTGGGCATTTTTTTGTTGGTGTGTGCATTTTT--------
hsa-mir-516a-2_13/7    ----------------------------------------------------------------------------------------------------------------------------------
hsa-mir-524_13/7       ----------------------------------------------------------------------------------------------------------------------------------
hsa-mir-520f_13/7      CGAACTCCTGAGCTCAAGTGATCCTCCTGCCTTGCCCTCTTTAAGTGCTGGGTTTATTGGTGTGAGCCACCACGCCCCACCCATACTTGTGTATTTCTGTATTTATTGAGGAGCTTATACAGCATACCCT
hsa-mir-520e_13/7      ----------------------------------------------------------------------------------------------------------------------------------
hsa-mir-517a_13/7      ----------------------------------------------------------------------------------------------------------------------------------
hsa-mir-517b_13/7      ----------------------------------------------------------------------------------------------------------------------------------
hsa-mir-517c_13/7      ----------------------------------------------------------------------------------------------------------------------------------

                       1691
hsa-mir-519b_13/7      --------------------------------------------------------------------------------------------------TTTAAGGCCTCCCTTGTAGTCTATCCTGGAGG
hsa-mir-523_13/7       ----CTGCCTCAGCCTCCCAAAGCATTGGGATTACAGGCGTGAGCCACCACGCCA-----------------------------------------------------------GGCCTGTTC-------
hsa-mir-518a-2_13/7    ----CTGCATCGGCCTCCCAAAGTGCTGGGATTATAGGCATGAGCCTCCACGCCTAGCC-------------------TCAAGTGGTCCTCTTGAGT-----CAACCTAGACTACAGTCTATGCTAGAGA
hsa-mir-519c_13/7      ----CTGCCTCAGCCTCCTAAAGGGCTGGGTTTAGAGGTGTGAGCCACCACACCTCACCTGTACCTGTGTGTTTCTGCTCA--TTTTATTGAGGAGCTTTTACAGCCTGGCCTGTGGTCTCTCCTGGAGG
hsa-mir-525_13/7       -----------------------------------------------------------------------CCGCTGTTCAAATTTTACTGAGGAGCTTTTAAGGCCTCGCCTGTGGTCTGTCCTGTA--
hsa-mir-518b_13/7      ----CTGCCTCCGACTCCTAAAGTGCTGGGATTACAGGTGTGAGCCACTGCACCCCACCTGTACTTGTGTATTTCTGTTCAAATTTTATTGAGGAGCTTTTACGGCCTAGACAGTAGTCTATCCTGGAGA
hsa-mir-520a_13/7      ----TCACTTCGGCGTCCTGAAGTGCTGGGGTTAGAGATGTGAGCCACCGCACCCAACAGGTACTTGTGTATTT---------TTTTATTGAGGAGCTTTTACAGCGTAGCCTGTGGTCTCTCCTGGAGG
hsa-mir-518f_13/7      ----CAGTTTCAGCCTCCTAATGTGCTGGGATTAGAGGTGTGAGCCACCGTGCCCCACTTGTAGTTGTGTGTTTCTGTTCAAATTTTATTGAGGAGTTTTTAGGGCCTAGCCTGTGATCTACCCTGGAGG
hsa-mir-522_13/7       ----CCATCACGGCCTCCTAAAGTGCTGGAATTAGAGGTGTGAGCCGCCACGCCCCACCTGTACTTGTGTATCTCTGTTCAAATGTTACTGAGGAGCTTTTACAGCGTAACCTGTGGTCTATCCTGGAAA
hsa-mir-526a-1_13/7    C---CCGCCTCAGCCACCCAAAGTGGTGGGATTACAGGCATAAGCCACTGCGCCCGGCC-----------------------------------------------------------------------
hsa-mir-518e_13/7      ----CCGCCTCGGCCTCCCAAAGTGCCGGGATTACAGGCGTGAGCCACCGCGCCCAGTCATCATATATT-----------------------------------------CTTATCATATATATAGTAGG
hsa-mir-516a-1_13/7    ----TGGCCTCGGCCTACCGAAGTGCTGGGATTACAGGCATGAGCCACCGCGCCCAG------CTTCTGTATTTCTTTTCAAATTTTATTGAGGATTTTTTATGTCCTAGCCTGTGGTCTATCCTGGAGG
hsa-mir-527_13/7       TCTCCTGCCTCAGCCTCCTGAGAAGCTAGGATTACAGGCATGCACCACCATGCCTG--------------------------------------------------------------------------
hsa-mir-526a-2_13/7    ----CCGCCTCGGCCTCCCAAAGTGCTGGGATTACAGGCATGAGCGACCG------------------------------------TATTGAGGCGCTTTTACAACATAGCCTGTGGTCTATCCTGGGGA
hsa-mir-518d_13/7      ----CTGCCTCAGCCTTCCAAACTGCTGGGATTACAGGCATGAGCCACTGCGCCTAGCCTCAAGTGGTCCTCTTAAGT------------------------CAGCCTAAACTGTGGTCTATCCTGGAGG
hsa-mir-521-2_13/7     ----CCGTCTTGGCCCCCTAATGTGCTAGGATTAGAGGTGTGAGCCAACCCACCCCACCCATACTTACGTATTTCTGTTCAGATTTTATT---GAGTTTTTATGACCAAGCCTATGGTCTATCCTGGAGA
hsa-mir-516b-2_13/7    ----T-GTCTCAGCCTCCTAAAGTGCTGGGATTAGAGGTGTGAGCCACCACACCCCACTGGTATTTGTGTGTTCCTGTTCAAATTTTATTGAGGAGTTTTTATAACCAAGCCTGTGGTCCATCCTGGAGG
hsa-mir-519a-1_13/7    ----CCGCCTCAGCCTCCTAAAGTGCTG------------TGAGCCACCGTGTCCCAACAGTACTTGTGTGTTTCCGTTCAAATTGTATTGAGGAGCTTTTACAGCATAGCCTGTGGTCTCTCCGGGAGA
hsa-mir-520c_58902519  ----C-ACCTTGGCCTCCTAAAGTGCTAGGATTAGTGGTGTGAGCTGTTGTGCCCCACCTATACTCGTCTGTTTCTGCTCAGATTTTATTGAGGAGCGTTTACAGGGTAGCCTGTTGTCTACCCTGCAGG
hsa-mir-518a-1_13/7    ----CTGCCTTGGCCTACGTAAGTGCTGCGGTTAGAGGTGTGAGCCACCGCGCCCCACCTGCCCTTGTGTATCTCTGTTCAAATGTTACTGAGAAGCATTTACGGGATAAT----------GCCCGGAGG
hsa-mir-521-1_13/7     ----CCACCTCGGCCTCCTAAACTGCTGTGATTAATGGTGTGAGCCACCGTGCCACACCCGTACTTGTTTGTTTCTGCTCAAATTGTATTGAGGAGCTTTTACATCCTAGTCTGTGGTCTATCCTGGAGG
hsa-mir-519a-2_13/7    ----AAACCTGGGCCTCCTAAACTGCTGTGATTAATGGTGTGAGCCACCGTGCCCTACTCTTACTTGTGTGTTTCTGCTCAAATTTTATTAAGGAGCTTTTATGGCCTCGCCTGTGGTCTGTCCTGGAGG
hsa-mir-520b_13/7      ----CCACCTCGGCCTCCTAAAGTGCTGTGATTAGAGGTATGAGCCACCGTGCCCCACCTGTACTTGTGTGTTTCTGCTCAAATTTTATTGGGGAGCTTTTATGGTATAGCCTGTGGTCT-CTCTGGAGA
hsa-mir-519e_13/7      ----CTGCCTCGGCCTCGCAAAGTGCTGGGATTACAGGCATGAGCCACTGTGCCTGGCCC----------------------------------------------------------------------
hsa-mir-518c_13/7      ----CCGCCTTCGACTCCTAAAGTGCTGGGATTAGAGGTGTAAGCCACTGCACTCCACCCGTACTTCTGTA--------CAAATGTTATTGAGGAGCTTTTACGGCCTAGGCAGTGCTCTATCCTGGAGA
hsa-mir-520g_13/7      ----CAGCCTTGGCCTCCTAAAGTGCTGGGATTACAGGCGTGAGCCACCGC-----------CCCTAATC--------TCACGTGGTCCTCTAGAGT-----CAGCCTAGACTCTGGTCTATCCTGGAGG
hsa-mir-519d_13/7      ----------------------------------------------------------------------------------------------ATC-----CA--CTAGCCTGTGGTCTATCCTGGAGG
hsa-mir-520h_13/7      ----CAGCCTTGGCCTCCCAAAGTACTGGGATTACAGCTGTGAGCCATCAC-----------CCCTAATC--------TCAAATGGTCCTGTAGAGT-----CAGCCTAGACTGTGGTCTATCTTGGAGG
hsa-mir-516b-1_13/7    ----CCGTCTTGGCCTCCTAAAGTGCTAGAATTAGAGGTGTGAGCCACCGTGCCCCACCTGTACTTGTGTATTTATGTTAAAATTGTGTTAAGGAGTTTTTATGACCAAGCCTGTGATCTATTCTGGAGA
hsa-mir-526b_13/7      ----CTGCCTGGGCCTCCTAAAGTTCGAGGATTAGAGGTCTGAGCCACTGGGCCCCACTTGTACTTGTGTATTTCTGCTCAAATTTTATTGAGTAGTTTTTACATCCTAGCCTGTGATCTACTCTGGAGA
hsa-mir-520d_13/7      ----------------------------------------------------------------------------------------------------------------------------------
hsa-mir-516a-2_13/7    ----CTGTCTTGGCCTCCTAAAGTGCTGGGATTAGAGGTGTGAGCCACCATGCCCCATCGGTACTTGTGATTTAGGTT-CAAATT---TTGATGAGTTTTTATGACCAAGCCTGTGGTCTATCCTAGAGA
hsa-mir-524_13/7       ----------------------------------------------------------------------------------------------------------------------------------
hsa-mir-520f_13/7      G---------------------------------------------------------------------------------------------------------------------------------
hsa-mir-520e_13/7      ---------CTCGGCCTCTCAAAGTGCTGAGATTACAGGCGTGAGCTACCCTGCCCAGGCTGACCATGTATATTCCTA----------------------------------------------------
hsa-mir-517a_13/7      ----CGGCCTCTGCCTCCCAAAGTGCTGGGATTACAGGCATGAGCCACCACGCCTGGCC-----------------------------------------------------------------------
hsa-mir-517b_13/7      ----------------------------------------------------------------------------------------------------------------------------------
hsa-mir-517c_13/7      ----------------------------------------------------------------------------------------------------------------------------------

                       1821
hsa-mir-519b_13/7      ATGTTTGTGTGTGCAACAATGTATATTCTTC---------------------------------------------------------------------------------------------------
hsa-mir-523_13/7       ----------------------------------------------------------------------------------------------------------------------------------
hsa-mir-518a-2_13/7    ATGTTTGTGTGTGTGACAATGTATGTTCTTG---------------------------------------------------------------------------------------------------
hsa-mir-519c_13/7      GTGTTTGTGTGTGTGACAATGCATATTCTTC---------------------------------------------------------------------------------------------------
hsa-mir-525_13/7       ----------------------------------------------------------------------------------------------------------------------------------
hsa-mir-518b_13/7      ATGTTTGTGTGTATGACAATGTATATTCTTTTTTTTTATTTTTATTTTTTGGAGACGGAGTCTCACTCTGTCACCCAGGCTGGAGTGCAATGGCATGATCTCGGTTCACTGAAACCTCCGCCTCCTGGGT
hsa-mir-520a_13/7      ATGTTTGTGTGTGCGACAATGTACATTCTTC---------------------------------------------------------------------------------------------------
hsa-mir-518f_13/7      ATGTTTGTGTGTACAACAATGTATATTCTTT---------------------------------------------------------------------------------------------------
hsa-mir-522_13/7       ATGTTTGTGTGTGAGACAATGTACATTCTTC---------------------------------------------------------------------------------------------------
hsa-mir-526a-1_13/7    --------------AACAATGTATATTTTTC---------------------------------------------------------------------------------------------------
hsa-mir-518e_13/7      ATGTTTGTGTATATAACAATGTACATTTTCA---------------------------------------------------------------------------------------------------
hsa-mir-516a-1_13/7    ATGTTTGTGTGTGAGACAATGTATATTCATC---------------------------------------------------------------------------------------------------
hsa-mir-527_13/7       ----------------------------------------------------------------------------------------------------------------------------------
hsa-mir-526a-2_13/7    ATGTTTGTGTGTGTGACAATGTATATTCTTA---------------------------------------------------------------------------------------------------
hsa-mir-518d_13/7      ATGCTTGTGTGTGTGACAATTTATATTCTTC---------------------------------------------------------------------------------------------------
hsa-mir-521-2_13/7     ATATTTGTGTATGTGACAATGTATATTCTTC---------------------------------------------------------------------------------------------------
hsa-mir-516b-2_13/7    ATGTTTGTGTGCATGACAATGTATATTCTTC---------------------------------------------------------------------------------------------------
hsa-mir-519a-1_13/7    ATGTTTTTGTTTACGACAATGTATATTCTTC---------------------------------------------------------------------------------------------------
hsa-mir-520c_58902519  ATGTTTGTGCCTACAACAGTGTATATTCTTC---------------------------------------------------------------------------------------------------
hsa-mir-518a-1_13/7    ATGTTTGTGTGTGTGACAGTGTACATTCTTC---------------------------------------------------------------------------------------------------
hsa-mir-521-1_13/7     ATGTTTGTGTATATAACAATGTACATTTTCA---------------------------------------------------------------------------------------------------
hsa-mir-519a-2_13/7    ATGTTTGTGTGTGTGACAATGTATATTCTTC---------------------------------------------------------------------------------------------------
hsa-mir-520b_13/7      ATGTTTGTGTCTATGACAATGTATATTCTTC---------------------------------------------------------------------------------------------------
hsa-mir-519e_13/7      ---------------ACAACGTATATTCTTAAAT------------------------------------------------------------------------------------------------
hsa-mir-518c_13/7      ATGTTTGTGTGTAAGACAATGCATATTCTTTTTTTCTTTCCTGTCACCCAGGCTGGAGTGCATTGGCACGATCTCGTCTCACTGCAACCTCCGCCT-------------------CCCGGGTTCAAGCAA
hsa-mir-520g_13/7      ATGTTTGTGTGTGAGACAATGTATATTCTTC---------------------------------------------------------------------------------------------------
hsa-mir-519d_13/7      ATGTTTGTGTGTGTGACAATGTATATTCTTC---------------------------------------------------------------------------------------------------
hsa-mir-520h_13/7      ATGTTTGTGTGTGAGACAATGTATATTCTTC---------------------------------------------------------------------------------------------------
hsa-mir-516b-1_13/7    ATATTTGTGCGTATGACAATGTATATTCTTC---------------------------------------------------------------------------------------------------
hsa-mir-526b_13/7      GTGTTTGTGCATATGACAGTGTATATTCTTC---------------------------------------------------------------------------------------------------
hsa-mir-520d_13/7      --------------------------------------TGTTTGTTCGTTCGTTTGTTTTTGAGACAAGATCTGGCCCTGTCACCTATGCTGGAGTGCAGTGGTGTGATCTCGGCTCCCGGCAACCTCAA
hsa-mir-516a-2_13/7    ATGTTTGTGTGTTTGACAATTCA-----------------------------------------------------------------------------------------------------------
hsa-mir-524_13/7       ----------------------------------------------------------------------------------------------------------------------------------
hsa-mir-520f_13/7      ----------------------------------TGGTCTATCCTGCAGGATGTTTGTGTGTGAGACAGTGTATATTCTTGAACATAGTAGATTCCATTTTGGATGCTCCCATCGGGACTGTGTGTCCCT
hsa-mir-520e_13/7      ---------------------------------------ATTTTTGTACTTTTTTTTTTTTGAGACGGAGATTCACTCTTGTGGCCCAAGCTGGAGTGCAATGGCATGATCTCGGCTCACTGCAACCTCT
hsa-mir-517a_13/7      ------------------------------------------TAATTTTTATATTTTCAGCAGAGGCGGGGTTTCACCATGTTGGCCAGGCTGGTTGCGAACTCCTGACCTCAGGTGATCTGCCCACCTC
hsa-mir-517b_13/7      ----------------------------------------------------------------------------------------------------------------------------------
hsa-mir-517c_13/7      ----------------------------------------------------------------------------------------------------------------------------------
```

```
                     1951
hsa-mir-519b_13/7    ----------------------------------------------------------------------------------------------------------------------------------
hsa-mir-523_13/7     ----------------------------------------------------------------------------------------------------------------------------------
hsa-mir-518a-2_13/7  ----------------------------------------------------------------------------------------------------------------------------------
hsa-mir-519c_13/7    ----------------------------------------------------------------------------------------------------------------------------------
hsa-mir-525_13/7     ----------------------------------------------------------------------------------------------------------------------------------
hsa-mir-518b_13/7    TCCAGCGATCCTCCTGTCTCAGCCTGCTGAGTAGCTAGGATTACAGGTGGGCGCCTG-GCTAA-------------------------------------------------------------------
hsa-mir-520a_13/7    ----------------------------------------------------------------------------------------------------------------------------------
hsa-mir-518f_13/7    ----------------------------------------------------------------------------------------------------------------------------------
hsa-mir-522_13/7     ----------------------------------------------------------------------------------------------------------------------------------
hsa-mir-526a-1_13/7  ----------------------------------------------------------------------------------------------------------------------------------
hsa-mir-518e_13/7    ----------------------------------------------------------------------------------------------------------------------------------
hsa-mir-516a-1_13/7  ----------------------------------------------------------------------------------------------------------------------------------
hsa-mir-527_13/7     ----------------------------------------------------------------------------------------------------------------------------------
hsa-mir-526a-2_13/7  ----------------------------------------------------------------------------------------------------------------------------------
hsa-mir-518d_13/7    ----------------------------------------------------------------------------------------------------------------------------------
hsa-mir-521-2_13/7   ----------------------------------------------------------------------------------------------------------------------------------
hsa-mir-516b-2_13/7  ----------------------------------------------------------------------------------------------------------------------------------
hsa-mir-519a-1_13/7  ----------------------------------------------------------------------------------------------------------------------------------
hsa-mir-520c_58902519 ----------------------------------------------------------------------------------------------------------------------------------
hsa-mir-518a-1_13/7  ----------------------------------------------------------------------------------------------------------------------------------
hsa-mir-521-1_13/7   ----------------------------------------------------------------------------------------------------------------------------------
hsa-mir-519a-2_13/7  ----------------------------------------------------------------------------------------------------------------------------------
hsa-mir-520b_13/7    ----------------------------------------------------------------------------------------------------------------------------------
hsa-mir-519e_13/7    ----------------------------------------------------------------------------------------------------------------------------------
hsa-mir-518c_13/7    TTCTCCTGCCTCAGCCTCCTGAGTAGCTGGGATTACAGGTGGGCACCACCACGCCTG-GCAAA-------------------------------------------------------------------
hsa-mir-520g_13/7    ----------------------------------------------------------------------------------------------------------------------------------
hsa-mir-519d_13/7    ----------------------------------------------------------------------------------------------------------------------------------
hsa-mir-520h_13/7    ----------------------------------------------------------------------------------------------------------------------------------
hsa-mir-516b-1_13/7  ----------------------------------------------------------------------------------------------------------------------------------
hsa-mir-526b_13/7    ----------------------------------------------------------------------------------------------------------------------------------
hsa-mir-520d_13/7    CCTCCCACCTCAGCCTCCTGAATTAGTGTCTACAGGCATGCACCTCTATACCTGGCTAACTTTTGTATTTTTACAGATGAGGTTTTACCATGTTTCCCAGGCTGGTGTCTAACTCCTGAG----------
hsa-mir-516a-2_13/7  ----------------------------------------------------------------------------------------------------------------------------------
hsa-mir-524_13/7     ----------------------------------------------------------------------------------------------------------------------------------
hsa-mir-520f_13/7    GTGCTGGAACTCGAGTGAACACTTGGCTCAAAATCCATTGCTGTTCTCTAGAAATCCAGCTCAATTCTCATGGTTAAATATAAGGTATATGTGGTAGGCATTGCTTTTTCTCTTTGGGGACAGAACTCAG
hsa-mir-520e_13/7    GCCTCCTGGGTTCAAGCAATTCTCCTGCCTCGGCCTCCCGAGTAGCTGGGATTACAGGTGTGCACCACCACGCCTGACTGACAATATATATATTCTTA--------------------------------
hsa-mir-517a_13/7    AGCCTCCCAAAGTGCTAAGATTACAGGCGTGAGCCACCACACATGGCCATTAACACCTTTTAGTCCATGGAAATTATTCTGGCACTGATTTATAAAGCCTCATGTGGGGTCAGGTAGAGTCAAAGGGGGA
hsa-mir-517b_13/7    ----------------------------------------------------------------------------------------------------------------------------------
hsa-mir-517c_13/7    ----------------------------------------------------------------------------------------------------------------------------------

                     2081
hsa-mir-519b_13/7    ----------------------------------------------------------------------------------------------------------------------------------
hsa-mir-523_13/7     ----------------------------------------------------------------------------------------------------------------------------------
hsa-mir-518a-2_13/7  ----------------------------------------------------------------------------------------------------------------------------------
hsa-mir-519c_13/7    ----------------------------------------------------------------------------------------------------------------------------------
hsa-mir-525_13/7     ----------------------------------------------------------------------------------------------------------------------------------
hsa-mir-518b_13/7    ----------------------------------------------------------------------------------------------------------------------------------
hsa-mir-520a_13/7    ----------------------------------------------------------------------------------------------------------------------------------
hsa-mir-518f_13/7    ----------------------------------------------------------------------------------------------------------------------------------
hsa-mir-522_13/7     ----------------------------------------------------------------------------------------------------------------------------------
hsa-mir-526a-1_13/7  ----------------------------------------------------------------------------------------------------------------------------------
hsa-mir-518e_13/7    ----------------------------------------------------------------------------------------------------------------------------------
hsa-mir-516a-1_13/7  ----------------------------------------------------------------------------------------------------------------------------------
hsa-mir-527_13/7     ----------------------------------------------------------------------------------------------------------------------------------
hsa-mir-526a-2_13/7  ----------------------------------------------------------------------------------------------------------------------------------
hsa-mir-518d_13/7    ----------------------------------------------------------------------------------------------------------------------------------
hsa-mir-521-2_13/7   ----------------------------------------------------------------------------------------------------------------------------------
hsa-mir-516b-2_13/7  ----------------------------------------------------------------------------------------------------------------------------------
hsa-mir-519a-1_13/7  ----------------------------------------------------------------------------------------------------------------------------------
hsa-mir-520c_58902519 ----------------------------------------------------------------------------------------------------------------------------------
hsa-mir-518a-1_13/7  ----------------------------------------------------------------------------------------------------------------------------------
hsa-mir-521-1_13/7   ----------------------------------------------------------------------------------------------------------------------------------
hsa-mir-519a-2_13/7  ----------------------------------------------------------------------------------------------------------------------------------
hsa-mir-520b_13/7    ----------------------------------------------------------------------------------------------------------------------------------
hsa-mir-519e_13/7    ----------------------------------------------------------------------------------------------------------------------------------
hsa-mir-518c_13/7    ----------------------------------------------------------------------------------------------------------------------------------
hsa-mir-520g_13/7    ----------------------------------------------------------------------------------------------------------------------------------
hsa-mir-519d_13/7    ----------------------------------------------------------------------------------------------------------------------------------
hsa-mir-520h_13/7    ----------------------------------------------------------------------------------------------------------------------------------
hsa-mir-516b-1_13/7  ----------------------------------------------------------------------------------------------------------------------------------
hsa-mir-526b_13/7    ----------------------------------------------------------------------------------------------------------------------------------
hsa-mir-520d_13/7    ----------------------------------------------------------------------------------------------------------------------------------
hsa-mir-516a-2_13/7  ----------------------------------------------------------------------------------------------------------------------------------
hsa-mir-524_13/7     ----------------------------------------------------------------------------------------------------------------------------------
hsa-mir-520f_13/7    GAGGATTGCCCCTTGATGAACAAGGCTAACCTGCTGATTCTTTGAAGCAAAGGACTGGAGATGGTCCTTTTAGGGGTTTATGTTCTGGATTCCAGAAAACATGCAAACAGGGCCAATAAATGCATCTTTT
hsa-mir-520e_13/7    ----------------------------------------------------------------------------------------------------------------------------------
hsa-mir-517a_13/7    CCTCAGTGTCCCTTGCAGATGGGATGTGCTCAGCAAGAGCACGGAGGTGGAGTGCATGGGGTTTGAGTTTTCACTGGGGAAATGAAACCAACATCTTGGGTGCATGACCAGGTCATATATGCAGTCATAT
hsa-mir-517b_13/7    ----------------------------------------------------------------------------------------------------------------------------------
hsa-mir-517c_13/7    ----------------------------------------------------------------------------------------------------------------------------------

                     2211
hsa-mir-519b_13/7    ----------------------------------------------------------------------------------------------------------------------------------
hsa-mir-523_13/7     ----------------------------------------------------------------------------------------------------------------------------------
hsa-mir-518a-2_13/7  ----------------------------------------------------------------------------------------------------------------------------------
hsa-mir-519c_13/7    ----------------------------------------------------------------------------------------------------------------------------------
hsa-mir-525_13/7     ----------------------------------------------------------------------------------------------------------------------------------
hsa-mir-518b_13/7    ----------------------------------------------------------------------------------------------------------------------------------
hsa-mir-520a_13/7    ----------------------------------------------------------------------------------------------------------------------------------
hsa-mir-518f_13/7    ----------------------------------------------------------------------------------------------------------------------------------
hsa-mir-522_13/7     ----------------------------------------------------------------------------------------------------------------------------------
hsa-mir-526a-1_13/7  ----------------------------------------------------------------------------------------------------------------------------------
hsa-mir-518e_13/7    ----------------------------------------------------------------------------------------------------------------------------------
hsa-mir-516a-1_13/7  ----------------------------------------------------------------------------------------------------------------------------------
hsa-mir-527_13/7     ----------------------------------------------------------------------------------------------------------------------------------
hsa-mir-526a-2_13/7  ----------------------------------------------------------------------------------------------------------------------------------
hsa-mir-518d_13/7    ----------------------------------------------------------------------------------------------------------------------------------
hsa-mir-521-2_13/7   ----------------------------------------------------------------------------------------------------------------------------------
hsa-mir-516b-2_13/7  ----------------------------------------------------------------------------------------------------------------------------------
hsa-mir-519a-1_13/7  ----------------------------------------------------------------------------------------------------------------------------------
hsa-mir-520c_58902519 ----------------------------------------------------------------------------------------------------------------------------------
hsa-mir-518a-1_13/7  ----------------------------------------------------------------------------------------------------------------------------------
hsa-mir-521-1_13/7   ----------------------------------------------------------------------------------------------------------------------------------
hsa-mir-519a-2_13/7  ----------------------------------------------------------------------------------------------------------------------------------
hsa-mir-520b_13/7    ----------------------------------------------------------------------------------------------------------------------------------
hsa-mir-519e_13/7    ----------------------------------------------------------------------------------------------------------------------------------
hsa-mir-518c_13/7    ----------------------------------------------------------------------------------------------------------------------------------
hsa-mir-520g_13/7    ----------------------------------------------------------------------------------------------------------------------------------
hsa-mir-519d_13/7    ----------------------------------------------------------------------------------------------------------------------------------
hsa-mir-520h_13/7    ----------------------------------------------------------------------------------------------------------------------------------
hsa-mir-516b-1_13/7  ----------------------------------------------------------------------------------------------------------------------------------
hsa-mir-526b_13/7    ----------------------------------------------------------------------------------------------------------------------------------
hsa-mir-520d_13/7    ----------------------------------------------------------------------------------------------------------------------------------
hsa-mir-516a-2_13/7  ----------------------------------------------------------------------------------------------------------------------------------
hsa-mir-524_13/7     ----------------------------------------------------------------------------------------------------------------------------------
hsa-mir-520f_13/7    TGTTTTGTTTTGTTTTGTTTTTGAGATGGAGTCTCGTTCTGTCACCCAGGCTGGAGTGCAGTGGCACAATCTTGGCTCACTGCAAGCTCAGCCTCCTGGGTTCACGCCATTCTCCTGCCTCAGCTTTCCA
hsa-mir-520e_13/7    ----------------------------------------------------------------------------------------------------------------------------------
hsa-mir-517a_13/7    ATGCAATATGCATGGTGGTGGGTGCCTCTAATCCCAGCTACTCAGGAGGCTGACGCAGAAGAATCGCTTGAACCCAGGAGGCAGAAGTTGCCATGAGCCGAGATCCCACCACCGCATCCAGCCTGGGCGG
hsa-mir-517b_13/7    ----------------------------------------------------------------------------------------------------------------------------------
hsa-mir-517c_13/7    ----------------------------------------------------------------------------------------------------------------------------------
```

```
                        2341
hsa-mir-519b_13/7       ----------------------------------------------------------------------------------------------------------------------------------
hsa-mir-523_13/7        ----------------------------------------------------------------------------------------------------------------------------------
hsa-mir-518a-2_13/7     ----------------------------------------------------------------------------------------------------------------------------------
hsa-mir-519c_13/7       ----------------------------------------------------------------------------------------------------------------------------------
hsa-mir-525_13/7        ----------------------------------------------------------------------------------------------------------------------------------
hsa-mir-518b_13/7       ----------------------------------------------------------------------------------------------------------------------------------
hsa-mir-520a_13/7       ----------------------------------------------------------------------------------------------------------------------------------
hsa-mir-518f_13/7       ----------------------------------------------------------------------------------------------------------------------------------
hsa-mir-522_13/7        ----------------------------------------------------------------------------------------------------------------------------------
hsa-mir-526a-1_13/7     ----------------------------------------------------------------------------------------------------------------------------------
hsa-mir-518e_13/7       ----------------------------------------------------------------------------------------------------------------------------------
hsa-mir-516a-1_13/7     ----------------------------------------------------------------------------------------------------------------------------------
hsa-mir-527_13/7        ----------------------------------------------------------------------------------------------------------------------------------
hsa-mir-526a-2_13/7     ----------------------------------------------------------------------------------------------------------------------------------
hsa-mir-518d_13/7       ----------------------------------------------------------------------------------------------------------------------------------
hsa-mir-521-2_13/7      ----------------------------------------------------------------------------------------------------------------------------------
hsa-mir-516b-2_13/7     ----------------------------------------------------------------------------------------------------------------------------------
hsa-mir-519a-1_13/7     ----------------------------------------------------------------------------------------------------------------------------------
hsa-mir-520c_58902519   ----------------------------------------------------------------------------------------------------------------------------------
hsa-mir-518a-1_13/7     ----------------------------------------------------------------------------------------------------------------------------------
hsa-mir-521-1_13/7      ----------------------------------------------------------------------------------------------------------------------------------
hsa-mir-519a-2_13/7     ----------------------------------------------------------------------------------------------------------------------------------
hsa-mir-520b_13/7       ----------------------------------------------------------------------------------------------------------------------------------
hsa-mir-519e_13/7       ----------------------------------------------------------------------------------------------------------------------------------
hsa-mir-518c_13/7       ----------------------------------------------------------------------------------------------------------------------------------
hsa-mir-520g_13/7       ----------------------------------------------------------------------------------------------------------------------------------
hsa-mir-519d_13/7       ----------------------------------------------------------------------------------------------------------------------------------
hsa-mir-520h_13/7       ----------------------------------------------------------------------------------------------------------------------------------
hsa-mir-516b-1_13/7     ----------------------------------------------------------------------------------------------------------------------------------
hsa-mir-526b_13/7       ----------------------------------------------------------------------------------------------------------------------------------
hsa-mir-520d_13/7       ----------------------------------------------------------------------------------------------------------------------------------
hsa-mir-516a-2_13/7     ----------------------------------------------------------------------------------------------------------------------------------
hsa-mir-524_13/7        ----------------------------------------------------------------------------------------------------------------------------------
hsa-mir-520f_13/7       AGTAGCTGGGGCTACAGGTGCCCACCACCACGCTAGGCTAATTTTTTGTATTTTTAGTAGAGGCGGGGTTTCACCCTGTTAGCCAGGATGGTCTTGATCTCCTGACCTCGTGATCTGCCCGCCTCGGCCT
hsa-mir-520e_13/7       ----------------------------------------------------------------------------------------------------------------------------------
hsa-mir-517a_13/7       CAGAGTGAGACTCCGTGTCAAAAAA---------------------------------------------------------------------------------------------------------
hsa-mir-517b_13/7       ----------------------------------------------------------------------------------------------------------------------------------
hsa-mir-517c_13/7       ----------------------------------------------------------------------------------------------------------------------------------

                        2471
hsa-mir-519b_13/7       ----------------------------------------------AACATGGTAGATTCCATTTTGACTGCTCTAATCAGGACTGTGTGTCCCTGTGCTGGAAATCAAGTGAACACTTGGCTGAAAATC
hsa-mir-523_13/7        ----------------------------------------------AACATCTTAGATTTCATTTTGGATGTTCCTGTGAGGACTGCGTGTCCCTGTGCTGGAACTCAAGTGAACGCTTGGCTCAAAATC
hsa-mir-518a-2_13/7     ----------------------------------------------AACATCATAGATTCCACTTTGGATGCTCCTGTCGGGACTGTGTGTCCCTGTGCTGGAACTCAAGTGAG-AGTTGGCTGAAAATC
hsa-mir-519c_13/7       ----------------------------------------------AACATCGTAGATTCCATTTTGGATGCCCCCGTCGGGACTGTGTGTCACTGTACTGGAACTCGAGTGAACACTTAGCTCAAAATC
hsa-mir-525_13/7        ---------------------------------------------------------------------------------------GTGTCCCTGTCCTGGAACTCAAGTGAATACTTGGTTTAAAATT
hsa-mir-518b_13/7       ---------------------------------------------------TTTAGATTCCATTTTGGATGCTCCCATCGGGACTGTGTGTCCCTATGTTGGAACTCAGGTGAACGCTTGGCTCAAAATC
hsa-mir-520a_13/7       ----------------------------------------------AACATCTTAGATTCCATTTTGGATGCCCCCATGGGTACTACATGTGCCTATACTGGAACTCAAGTGAACACTTGGCTCAAAATC
hsa-mir-518f_13/7       ----------------------------------------------AACATCGTAGATTCCATTTTGGATGCTCCCATCGGGACTGTGTGTCCCTGTGCTGGAACTCTGGTGAACACCTGGCTCAATATC
hsa-mir-522_13/7        ----------------------------------------------AACATCTTAGATTGCAGTTTGGATGCTCCCGTCAGGACTGTGTGTCCCTGTGCTGGGACTCAAGTGAACACTGGGCTCTCCATC
hsa-mir-526a-1_13/7     ----------------------------------------------AACATGGGAGATTCAGTTTTGAATGCTCCCATCGGGACTGTGTGTCCCTGTGCTGGAACTCAAGTGAACGCTTGGCTCAAAATC
hsa-mir-518e_13/7       -----------------------------------------------ACATTGTAGATTCCATTTTGGATGCTCCCATTGGGACTGTGTGTCCCTGTGCTGGAACTCAAGTGAACACTTGGCTCAAAATC
hsa-mir-516a-1_13/7     ----------------------------------------------AACATCTTAGATTCCATTTTGATTGCTTCCGTAGAAACTGTGTGTCCTTGTGCTGGAACTCCGGTGAACACCTGGCTCAATATC
hsa-mir-527_13/7        ----------------------------------------------GCTAATTTAGATTCCATTTTGGATGCTCCCATCAGGACT--GTGTCCCTGTACTGGAACTCAGGTGAACACTTGGTTCAAAATC
hsa-mir-526a-2_13/7     ----------------------------------------------GACATCTTAGATTCCATTTTGGATGCTCCCATCGGTACTATGTGTTCCTATATAGGAACTCAAGTGAACACTTGGCTTAAAATC
hsa-mir-518d_13/7       ----------------------------------------------AACGTCTTAGATTCTATTTTGGAGGCTCCCAAGGGGATTGTGTGTCCCTGTGCTGGAACTCAAGTGAACACTTGGCTGTACATC
hsa-mir-521-2_13/7      ----------------------------------------------AACATCGTAGACTCGATTTTGGGTGCTCCCATCGGGACTGTGTGTACCTGTCCTGGAACTCAAGTGCACCCTTGGCTCATAATC
hsa-mir-516b-2_13/7     ----------------------------------------------AACATCGTAGACTCGATGTTGGATGCTCCCATCGGGACTGTGTGTCCCTGTACTGGAACTCGAGTGAACACTTGGCTCTAAGTT
hsa-mir-519a-1_13/7     ----------------------------------------------AACATCGTAGATTCCATTTTGGATGCCCTCATTGGGACTGTGTGTCCCTGTACTGGAACTCAAGTGAACACTTGGCTCAAAATC
hsa-mir-520c_58902519   ----------------------------------------------AACATCTTAGATTCCATTTCGGATGCCCCCATGAGGACTGTGCGCTCCTGTACTGGAACTCAAGCGACCACTTGGCTCAAAATC
hsa-mir-518a-1_13/7     ----------------------------------------------AACATCTTAGATTCCATTTTGGATGCTCCCCTCGGGACTGTGTGTCTCTGTCCTGGAATTCAAGTGAACACCTGGCTCTCCATC
hsa-mir-521-1_13/7      -----------------------------------------------ACATTTTAGATTCCATTTTGGATGCTCCCATCGGGACTGTGTGTCCCTGTGCTGGAACTCGAGTGAACACTTGGCTCAAAATC
hsa-mir-519a-2_13/7     ----------------------------------------------AACATCTTCGATTCCATTTTGGGTGCCTCCATGGGGACTGTGTGTCCCTGTACTGGAACGCAAGTGAAGACTTGGCTCAGAGTC
hsa-mir-520b_13/7       ----------------------------------------------AGTATGTTATGTTCCATTTTGGATGCTTGCATGGGGACTGTGTGTCCCTGTACTGAAACTCAGGTGAACACTTGGCTCTCCGTC
hsa-mir-519e_13/7       ----------------------------------------------ATCATCTTAGATTCCATTTTGGTTGCTCCCATCGGGACTGTGTGTCCCTGTGCTGGAACTCAAGTGAACACATGGCTCAAAATC
hsa-mir-518c_13/7       ---------------------------------------------------TTTAGATTCCATTTTGGATGCTCCCATCATGACTGTGTGTCCCTGTGCTGGAACTCAAGTGACCATGTGACTCAA--TC
hsa-mir-520g_13/7       ----------------------------------------------AACATC------------------------------------------------------------------------------
hsa-mir-519d_13/7       ----------------------------------------------AACATCTTAGACTCCATTTTGGATGCTCCCATCGGAACTATGTATCCCTGTGCTGGAACTCAAGTGAACACTCAGCTCAAAGTC
hsa-mir-520h_13/7       ----------------------------------------------AACATTGTAGATTCCATTTTGGATGCTTCCATCGGGACTGTGTGCCCCTGTCCTGGAACTCAGCTGAACACTTGGCTCAAAATC
hsa-mir-516b-1_13/7     ----------------------------------------------AACATCGTAGATTCCATTTTGGATGCTCCCACTGGGACTGTGTG--CCTATACTGGAACTCAAGTGAACACTTGGCTCAAAATC
hsa-mir-526b_13/7       ----------------------------------------------AACATCGTAGACTCCATTTTGGATGCTCCCTTCGTGACTGTGGGT-CCTGTACTGGAACTCGAGTGAACACTTGGCTTAAAATC
hsa-mir-520d_13/7       ----------------------------------------------------------------------------------------------------------------------------------
hsa-mir-516a-2_13/7     -----------------------------------------------ACATCGTAGAGTTGATTTTGGGTGCTCCCATTGGGACTGTGTGTCCCTGTACTGGAACTTGAGTGAACACTTGGCTCATAATC
hsa-mir-524_13/7        ----------------------------------------------------------------------------------------------------------------------------------
hsa-mir-520f_13/7       CCCAAAGTGCTGGGATTACAGGCATGAGCCACTGCGCCCGGCCCC-------------------------------------------------------------------------------------
hsa-mir-520e_13/7       ----------------------------------------------AACAGCTCAGATTCCATTTTGGATCCTCCCATCAGGACTGGACTTAGGTGTACTGGAACTTAGGTGAACACTTGGCTCAAAATT
hsa-mir-517a_13/7       ----------------------------------------------------------------------------------------------------------------------------------
hsa-mir-517b_13/7       ----------------------------------------------------------------------------------------------------------------------------------
hsa-mir-517c_13/7       ----------------------------------------------------------------------------------------------------------------------------------

                        2601
hsa-mir-519b_13/7       CACTGCTGTTCTCTAGAAATCCAGCCCAATTCTCTTGGTTAAATATAAGGTATGCATAGTAGGCATTGCTTTTTCTTTCTGGACACAAAACTCAGGAGGATTTCCCCTTGATGAACAAGGCTAACCTGCT
hsa-mir-523_13/7        CATTGCTGTTCTCTAGAAATCCAGCCAAATTCTATTGGTGAAATATAAGGTATGTCTAGTAGTCATTGCTTTTTCTCTTTGGAGACAAAACTCAGGAGGATTGCCCCTTGATGAACAAGGCTAACCTGCT
hsa-mir-518a-2_13/7     CATTGCTGTTCTCTAGAAATCCAGCCCAAGTCTCTTGGTTAGAGATAAGGTATGTCTAGTGGGCATTGCTTTTTCTTTTTGGGGACAAAACTCAGAAGGATTGCCCCTTGATGAACAAGGCTAACCTGCT
hsa-mir-519c_13/7       TATTGCTGTTCTCTAGAA-TCCAGCCCAATTCTCATGGTTAAATATAAGGTATGTATAGTCGGCATTGCTT-----------------------------------------------------------
hsa-mir-525_13/7        GATTGCTGTTCTCTAGAAATCCAGCCCAATTCTCTTGGTTAAATATAAGGTATGTGTAGTAGGCACTGCTTTTTCTTTCTGGAGGCGAAACTCAGGAGGATTGCCCCTTGATGAACAAGGCTAACCTGCT
hsa-mir-518b_13/7       CATTGCTGTTCTCTAGAAATCCTGCCTAATTCTCTTGGTTAAAGATAAGGTATATGTAGCAGGCATTGCTTTTTCTCTTTGGGGACAAAACTCAGGAGGATTGCGCCTTGATGAACAAGGCTAACCTGCT
hsa-mir-520a_13/7       CATTGCTGTTCTCTAGAAATTCAGTTCGATTCTCTTGGTTAAAGATAAGGTATGAGTAGTAGGCATTGCTTTTTCTCTTTGGGGGCAAAACTCAGGAGGATTGCCCCTTGATGAACAAGGCTAACCTGCT
hsa-mir-518f_13/7       CATTTCTCTTCTCTAGAAATCCAGCCCAGTTCTCTTGGTTAAATATAAGGTATGTCTATGAGGCATTGCTTTTGCTCTTTGAGAACAAAACTCAGGAGGATTGCTCTTTGATGAACAAAGCTAACCTGCT
hsa-mir-522_13/7        CATTGCTGTTGTCTAGAAATCCAGCCCAATTCTCTTGGGTAAATATGAGGTATGTGTAGTAGGCATTGCTTTTTCTTTCTGGAGACAAAGCTCAGGAGGATTGCCCCTTGATAAACAAAGCTAACCTGCT
hsa-mir-526a-1_13/7     CATTGCTGTTCTCTAGAAATCCAGCCCAATTCTCTTGGTTAAATGTAAGGTATGTGTAGTAGGCAATGCTTTTTCT----GGAGACAGAACTCAGGAGGATTGTCCCTCGATGATCTAGGCTAACCTGCT
hsa-mir-518e_13/7       CATTGCTGTTCTCTAGAAATCCAGCCCAATTCTCTTGGTTAAATATAAGGTATGTGTAGTAGGCATTGCTCTTTCTCTTTAGAGACAAAGCTCAGGA---TTGCCCCTTGATGAACAAGGCTAACCTGCT
hsa-mir-516a-1_13/7     CATTGCTCTTCTCTAGAAATCCAGCCCAGTTCTCTTGGTTAAATATAAGATATGTGTAGCAGGCATTGCTTTTTCTTTCCAGAGACAAAACTCAGGAGGATTGCCCCTTGATGAACAAAGCTAACCTGCT
hsa-mir-527_13/7        CGTCGCTTTTCTCTAGACATCCAGCTCAGTTCTC---GGTAAAGATAAGGTATGTGTAGTAGGCATTGCTTTTTCCCTTTAGAGACAAAACTCAGGAGGATTGCCCCTTGGTGAACAAGGCTAACTTGCA
hsa-mir-526a-2_13/7     CATTGCTCTTGTCTAGAAATTTAGCCCAGTTATCTTGGTTAAAGATAAGGTGTGTCTGGTAGGCATTGATTTTTCTTTCCGGAGAAAAAACTTAGGAGGATTTCCTCTTGATGAACAAGGCTAGCCTGCT
hsa-mir-518d_13/7       CATTGCTGTTCTCTAGAAATTCAGCCCAATTCTCTTGGTTAAAGATAAGGTATGTGTAGTAGGCATTGCTTTTTCTTTCCAGAGACAAAACTCAGGAGGATTGCCCCTTGATGAGCAAGGCTAACCTGCT
hsa-mir-521-2_13/7      CATTGCTCTTCTCCAGAAATCTTACC-AATTCTCCTGCTTAAATATAAGCTACGTGTAGTAGGCATTGTTTTTCTTAGCAGATACGAAACTCAGGAAGATTGTCCTATGATAAACAAGGCTAACCTGCT
hsa-mir-516b-2_13/7     CATTGCTGTTTTCTAGAAATCCAGCCCAATTCTCTTGGTTAAATATAAGGTGCACGTAGTAGGCATTGCTTTTTCTTTCTGGAGACAAAACTCAGGAGGATTGCCCCTTGATGAACAAAGCTAACCTGCT
hsa-mir-519a-1_13/7     CATTGCTGTTCTCTAGAAATCCAGCCTAATTCTCTTGGTTAAAGGTAAGGTATGAGTAGTAAGCATTGCTTTTTCTCTTTGGGAACAAAACTCAGGAGGATTGCCCCTTGATGAACAAAGCTAACCTGTT
hsa-mir-520c_58902519   CATTGCTATTCTCTAGAAATCCAGCCCAATTCTCTTGGTTAAAGATAAGGTATGTGTAGTAGGCATTGCCTTTTCTCTTTGGGAACAAAACTCAGGAGGATTGCCCGTTGATGAACAAGGCTAACCTGCT
hsa-mir-518a-1_13/7     CATTGCTGTTCTCTAGAAATCCAGCCCAATTCTCTTGGTTAAATATAAGGTAGGTGTAGTAGGCATTGCTTTTTCTCTTTGGGGACAAAACTCAGGAGGATTGCCCCTTGATGAACAAGGCTAACCTGCT
hsa-mir-521-1_13/7      CATTGCTGTTCTCTAGAAATCCTGCCCTATTCTCTTGGTGAAATATAAGGTATGTCTAGTCGGCATTGATTTTTCTTTCTGGAGACAAAACTCAGGAGGGTTGCCCCTGCATGAACAAGGCTAACCTGCT
hsa-mir-519a-2_13/7     CATTGCTGTTCTCTAGAAATCCAGCCTAATCCTCTTGTGCAAATATAATATATATCTAGTAGGCATTGCTTTTTCTTTCTGGAGACAAAACACAGGAGGATTGCCCCTTGATGAACAGGACTAACCTGCT
hsa-mir-520b_13/7       CATTGCTGTTCTCTAGAAATCCAGCCCAGTTCCCTTGGTTAAAGATAAGGTATGTCTAGCAGGTATTGCTCTTTCTCT-TGCAGACAAAACTCAGGAGGATTGCCCCTTGATAAACAAGGCTAACCTGCT
hsa-mir-519e_13/7       CATTGCTGTTCTCTAGAAATCCAGCCCAATTCTCTTGGTTAAATATAAGGTATGTGTGGTAGGCTTTGCTTTTTCTCTTTGGAGACAATACTCAGGAGGGTTGCCCCTTCGTGAACAAGGCTAACCTGCT
hsa-mir-518c_13/7       CATTGCTGTTCTCTAGAAATCCACCTCAATTCTCTTGGTTAAAGGTAAGGTATGTGTAGTGGGCATTGCTTTTTCTCCTTGGAGACAAAACTTAGGAGGATT-CCCCTTGATGAACAAGGCTAATCTGCT
hsa-mir-520g_13/7       ----------------------AGCCCAATTCTCTTAGCTAAATGTAAGGTATGAGTAGTAGGTATTCCTTTTTCTCTTTGGGGACAAAACTCAGGACGATCGCCCCTTGATGAACAAGGCTAACCTGCT
hsa-mir-519d_13/7       CATTGCTGTTCTCTAGAAATCCAGCCCAGTTCTCTTGGTTAAATATAAGGTATGTGTAGTAGGCATTGCTTTTTCTCTTTAGAGGCAAAACTCAGGAGGGTTGCCCCTTGATGAACAAGGCTAACCTGCA
hsa-mir-520h_13/7       CATTGCTGTTCTCTGGAAATTCTGCCCTAATCTCTTGGTTAAATATAAGGTATGTGTAGTAGGCTTTGCTTTTTCTCTTTGGGGACAAAACTCAGGAGGGTTGCCCCTTGGTGAACAAGGCTAACCTGCT
hsa-mir-516b-1_13/7     CATTGCTGTTCTCTAGAAATCCAGCCCAATTCTCTTGGTTAAATATAAGGTATGTGTAGTAGGCATTGCTTTTTCTTTCTAGAGACAAAACTCAGGAGGATTGCCCCTTGATGAACAAGG-TAACCTGCT
hsa-mir-526b_13/7       CATTGCTGTTCTCCAGAAATCCTGCCCAATTTTCTCGGTTAAA-----GGTACGTGTAGTAGGCATTGCTTTTTCTCTTTGGGGACAAAACTCAGGAGGATTGCCCCTTGATGAACAAGGCTAACCTGCT
hsa-mir-520d_13/7       -----------------------------------------AATATAAGGTATATGTAGTAGGCGTTGCTTTTTCTCTTTGAAGACAAAACCCAGGACAGTTGTCCCTCGATGAACAAGGCTAACCTGCT
hsa-mir-516a-2_13/7     CATTGCTCTTCTCTAGAAATCCAGCCCAATTCTCTTGGTTAAATATAAGGTATGTGTAGCAGGCATTGCTTTTTCCTTCCAGAGACAAAACTCAGGAGGATTGCCCCTTGATGAACAAGGCTAACCTGCT
hsa-mir-524_13/7        --------------------------------------------------------------------------------------------------------------------AAGGCTAACCTGCT
hsa-mir-520f_13/7       ----------------------------------------------------------------------------------------------------------------------------------
hsa-mir-520e_13/7       CATTGCTGTTCTCTATAAATCTAGCC-AGTTCTCTTGGTTAAATTTAAGGTATGTATAGTAGTCGCTGCTTTTTCTTTCGGGGGACAAAACTCAGGAGGATTGCTTCTTGATTCATAAGGGCAACCTGTT
hsa-mir-517a_13/7       ----------------------------------------------------------------------------------------------------------------------------------
hsa-mir-517b_13/7       ----------------------------------------------------------------------------------------------------------------------------------
hsa-mir-517c_13/7       ----------------------------------------------------------------------------------------------------------------------------------
```

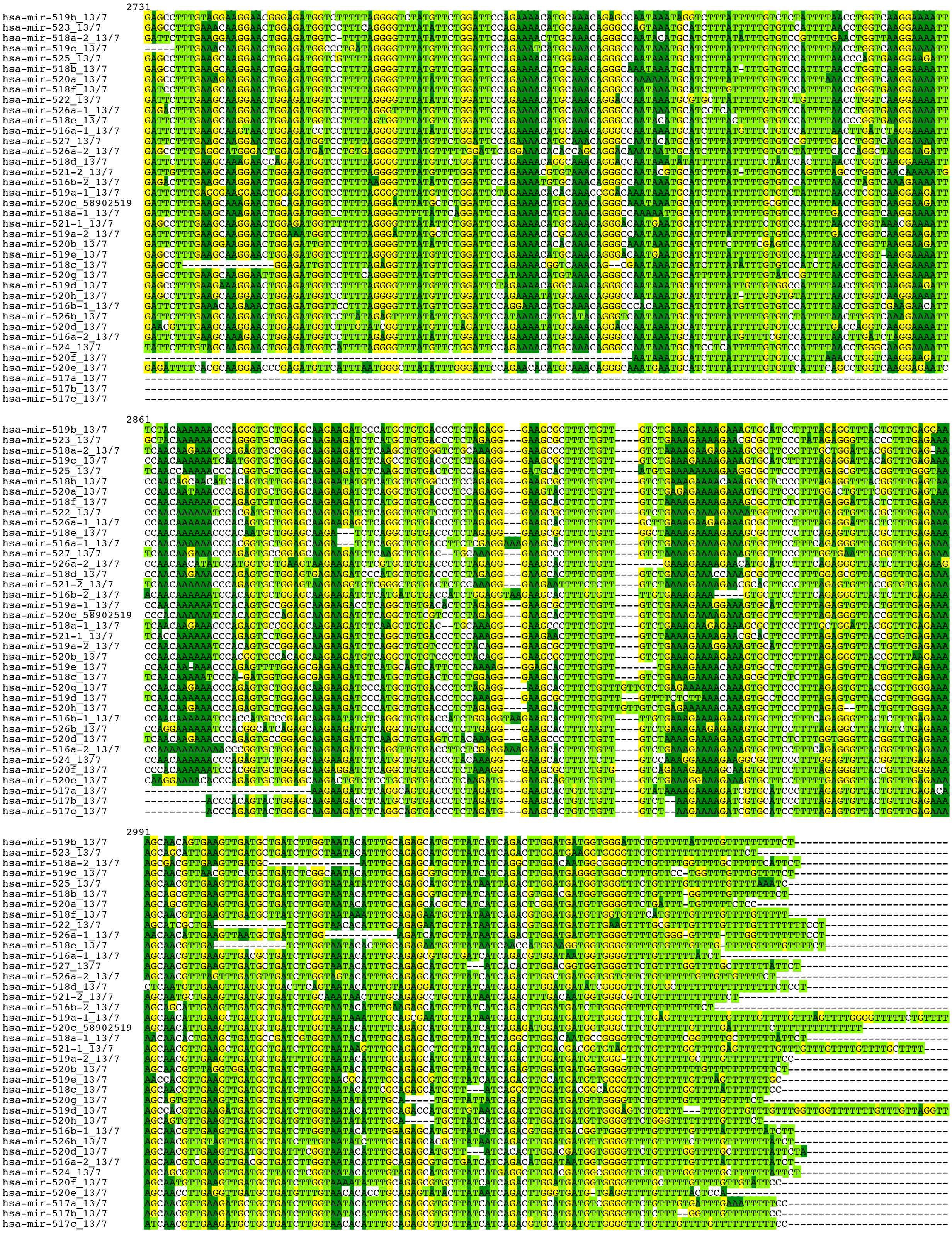
2731
hsa-mir-519b_13/7
hsa-mir-523_13/7
hsa-mir-518a-2_13/7
hsa-mir-519c_13/7
hsa-mir-525_13/7
hsa-mir-518b_13/7
hsa-mir-520a_13/7
hsa-mir-518f_13/7
hsa-mir-522_13/7
hsa-mir-526a-1_13/7
hsa-mir-518e_13/7
hsa-mir-516a-1_13/7
hsa-mir-527_13/7
hsa-mir-526a-2_13/7
hsa-mir-518d_13/7
hsa-mir-521-2_13/7
hsa-mir-516b-2_13/7
hsa-mir-519a-1_13/7
hsa-mir-520c_58902519
hsa-mir-518a-1_13/7
hsa-mir-521-1_13/7
hsa-mir-519a-2_13/7
hsa-mir-520b_13/7
hsa-mir-519e_13/7
hsa-mir-518c_13/7
hsa-mir-520g_13/7
hsa-mir-519d_13/7
hsa-mir-520h_13/7
hsa-mir-516b-1_13/7
hsa-mir-526b_13/7
hsa-mir-520d_13/7
hsa-mir-516a-2_13/7
hsa-mir-524_13/7
hsa-mir-520f_13/7
hsa-mir-520e_13/7
hsa-mir-517a_13/7
hsa-mir-517b_13/7
hsa-mir-517c_13/7
2861
2991

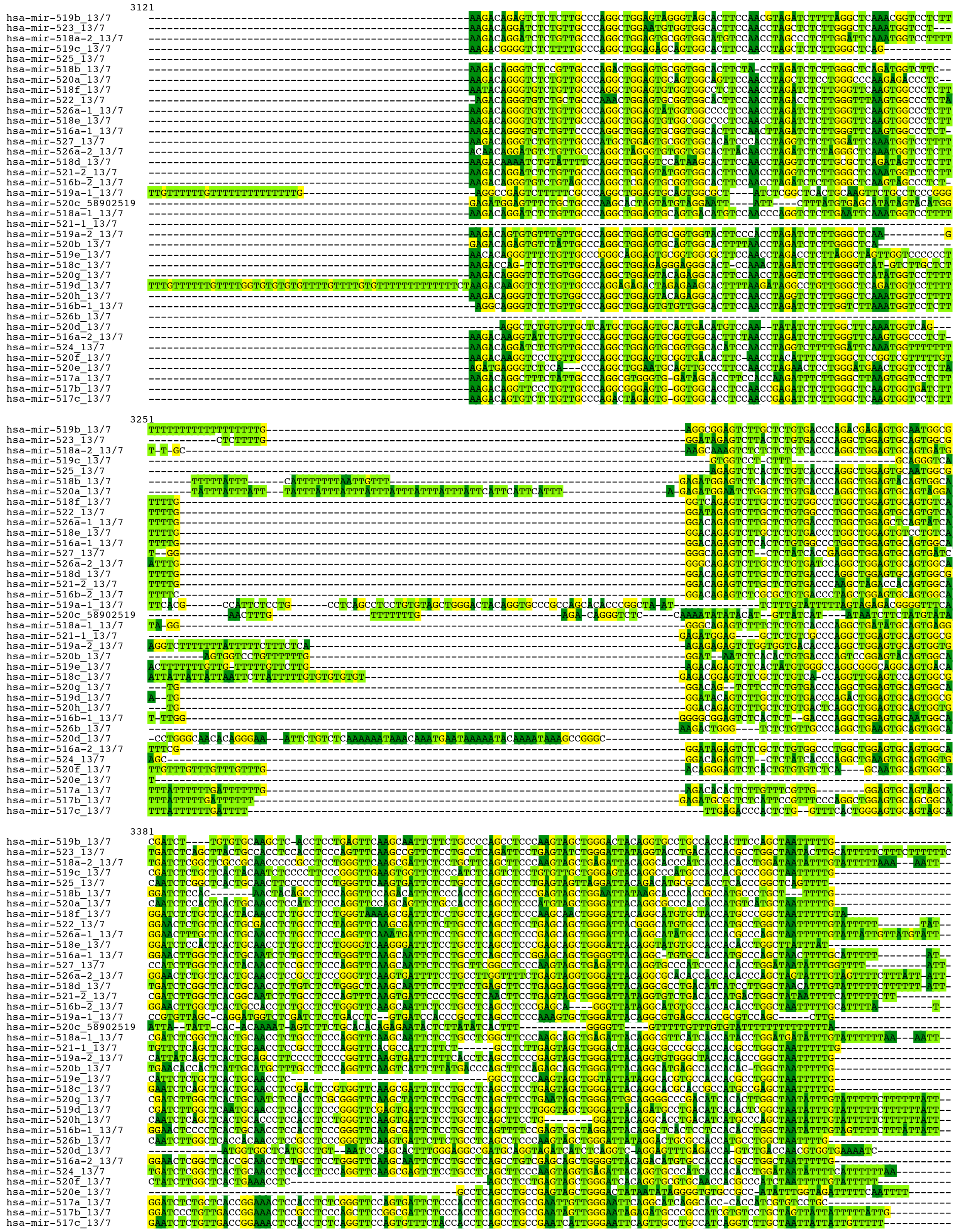
3121
3251
3381
hsa-mir-519b_13/7
hsa-mir-523_13/7
hsa-mir-518a-2_13/7
hsa-mir-519c_13/7
hsa-mir-525_13/7
hsa-mir-518b_13/7
hsa-mir-520a_13/7
hsa-mir-518f_13/7
hsa-mir-522_13/7
hsa-mir-526a-1_13/7
hsa-mir-518e_13/7
hsa-mir-516a-1_13/7
hsa-mir-527_13/7
hsa-mir-526a-2_13/7
hsa-mir-518d_13/7
hsa-mir-521-2_13/7
hsa-mir-516b-2_13/7
hsa-mir-519a-1_13/7
hsa-mir-520c_58902519
hsa-mir-518a-1_13/7
hsa-mir-521-1_13/7
hsa-mir-519a-2_13/7
hsa-mir-520b_13/7
hsa-mir-519e_13/7
hsa-mir-518c_13/7
hsa-mir-520g_13/7
hsa-mir-519d_13/7
hsa-mir-520h_13/7
hsa-mir-516b-1_13/7
hsa-mir-526b_13/7
hsa-mir-520d_13/7
hsa-mir-516a-2_13/7
hsa-mir-524_13/7
hsa-mir-520f_13/7
hsa-mir-520e_13/7
hsa-mir-517a_13/7
hsa-mir-517b_13/7
hsa-mir-517c_13/7

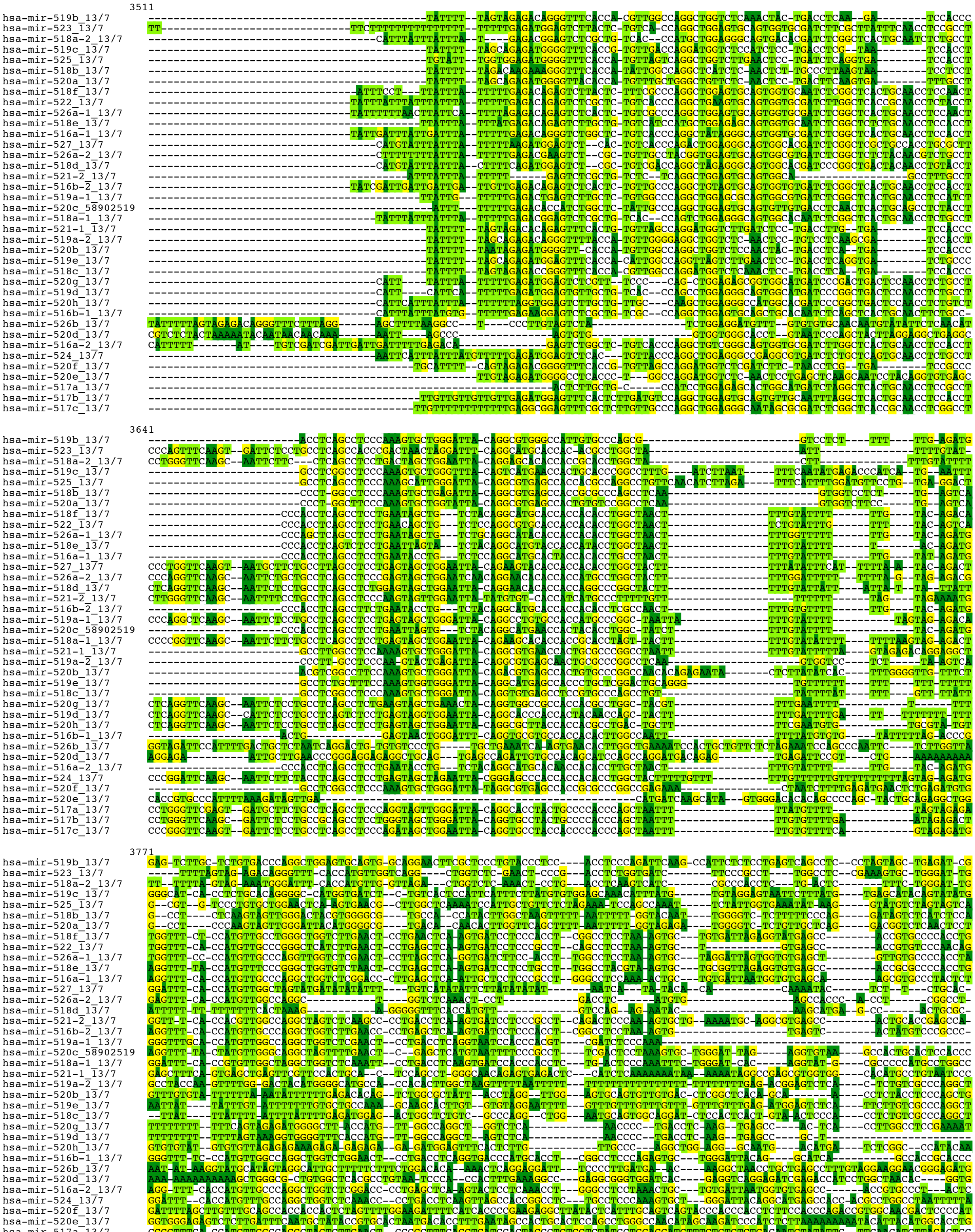
3511
hsa-mir-519b_13/7
hsa-mir-523_13/7
hsa-mir-518a-2_13/7
hsa-mir-519c_13/7
hsa-mir-525_13/7
hsa-mir-518b_13/7
hsa-mir-520a_13/7
hsa-mir-518f_13/7
hsa-mir-522_13/7
hsa-mir-526a-1_13/7
hsa-mir-518e_13/7
hsa-mir-516a-1_13/7
hsa-mir-527_13/7
hsa-mir-526a-2_13/7
hsa-mir-518d_13/7
hsa-mir-521-2_13/7
hsa-mir-516b-2_13/7
hsa-mir-519a-1_13/7
hsa-mir-520c_58902519
hsa-mir-518a-1_13/7
hsa-mir-521-1_13/7
hsa-mir-519a-2_13/7
hsa-mir-520b_13/7
hsa-mir-519e_13/7
hsa-mir-518c_13/7
hsa-mir-520g_13/7
hsa-mir-519d_13/7
hsa-mir-520h_13/7
hsa-mir-516b-1_13/7
hsa-mir-526b_13/7
hsa-mir-520d_13/7
hsa-mir-516a-2_13/7
hsa-mir-524_13/7
hsa-mir-520f_13/7
hsa-mir-520e_13/7
hsa-mir-517a_13/7
hsa-mir-517b_13/7
hsa-mir-517c_13/7
3641
3771

```
                         3901
hsa-mir-519b_13/7        CAG-------ACATGA-------GCCACCATACCCAGCTAAT-----------TTTTGTATT------TTTATTATTTATTTATTTACTCAT-CGATTTTTGAAACAGAGTCTT----------------
hsa-mir-523_13/7         CAG-------GCATTA-------GCCACCCCGCCCGGCCAT-------------TATATATT------CT---TATATATATAATACCATAT-ACGTG--------------------------------
hsa-mir-518a-2_13/7      C-----AG--GTAT-------GCGCCCCCACGCCTGGCCTAAT----------TTTTGAATG------TTTAGCAGAGATGGGGTTTCACC--ATGTTGGCCAGGCTGAACTCAGC--------------
hsa-mir-519c_13/7        GAACTTTC--TTTTTTTTTGAGATAGGG-TCTGGAAAATAAA---------TGATGTACA--------GCCAGGCGCATGAGTATATGAGCAGTTTTTGTGTGTATACGACATGAGCGGTGGCTCATGCC
hsa-mir-525_13/7         TTGCTTT---TTCTCTTTGGAGACAAAACTCAGGAGGATTGCCCC-----TTGATGAACAAG------GCTAACCTGCTGAGCCTTTGA--AACAAGGAACTGGAGATGGTCCTTTCAGGGGTTTATATT
hsa-mir-518b_13/7        CAGCTTAT--GTGGTCCTACACGTGTGAGCCACCTCGTCT-----------TGATGA-----------CCCATTTCAAAGAGAGTTGACATGGCCAGGCATTGTGGGGCACACAGTCC------------
hsa-mir-520a_13/7        GAGCTCAA--GCGATCCTACAGGTGTGAGCCACCTTGTCC-----------TGATGA-----------CCCATTTCAAAGA-------------------------------------------------
hsa-mir-518f_13/7        TACT-TGT--GTGTTTCTGCT------------CAAATTTTA--------TTGGGGAGCTTTT---ATGGTATA--GCCTGTGGTCTCTCTGG-AGAAT-------------------------------
hsa-mir-522_13/7         TACT-TGT--GTGTTTCCGTT------------CAAATTGTA--------TTGAGGAGCTTTT---ACAGCATA--GCCTGTGGTCTCTCCGGGAGAATGTTTTTGT-----------------------
hsa-mir-526a-1_13/7      TACT-CGT--CTGTTTCTGCT------------CAGATTTTA--------TTGAGGAGCGTTT---ACAGGGTA--GCCTGTTGTCTAC-----------------------------------------
hsa-mir-518e_13/7        CCCT-TGT--GTATCTCTGTT------------CAAATGTTA--------CTGAGAAGCATTT---ACGGGATAATGCCCGGAGGATGTTTGTGTGTGTGACAGTGTACATTC-----------------
hsa-mir-516a-1_13/7      T-----GT--GTGTTTCTGCT------------CAAATTTTA--------TGGAGGAGCTTTT---ACATCCCA--GCCTGTGGTCTATCCTGGAGAATGTTTGTG------------------------
hsa-mir-527_13/7         ------AC--G-AAGAGTATT------------CTTATATCGC---------TTTGGGTTTTT------TTGTT---TGTGTG-TTTGTTTTGT-GTTTGTTTCTTTGTTT-------------------
hsa-mir-526a-2_13/7      ------TC--CAAA--GTGC------------TGGAATTACAG--------GCATGA---ACC-----ACGACGTCTGGC----CTAATTTTGTATT---TTTAGAAGAGA-------------------
hsa-mir-518d_13/7        -----AGC--CATCA-ACACC-----------TTTTACTGCATGG----------AAATTATT-----CTGGCA-CTGGT---ATAGAACCTCACATGGGGTCAGGTGGAG-------------------
hsa-mir-521-2_13/7       ---T-CAA--CACCTTCTACTC--CATGG-------AAATGATA------CTGGCGCTGGTTT---ATAGAACCTCACTTGGGGTCGGGCAG-ATTTAAAAGGGACCTCAGGC-----------------
hsa-mir-516b-2_13/7      TACT-TGT--GTGTTTCTGCT---TACATTTTATTGAGGCGA--------TTTTTTTTTTTTT---TTTGAGACGGAGTCTTGGTCTGTTA---CCTAGGCTGGAGCGCAGT------------------
hsa-mir-519a-1_13/7      ----------------------------------------------------------------------------------------------------------------------------------
hsa-mir-520c_58902519    GTACTTCT--GTACAAATGTTAT-TGAGGAGCTTTTACGGCCTAG--GCAGTGCTCTATCCTGGAGAATGTTTGTGTGTAAGACAATG------------------------------------------
hsa-mir-518a-1_13/7      TAATTTTT--GTATT---TTTAGCAGAGACGTGGTTTCAC------------------------------------------------------------------------------------------
hsa-mir-521-1_13/7       AGCCCTTT--GGGAGGCCGAGACAGGTGGATCACCTGAGGTCAGG------AGTTAAAGGCTGGCTGGGCCAACAAGGCAAAACCTCGACTCTACTAAAAATACAAAATTACACCTGTAATCTTAGCTGC
hsa-mir-519a-2_13/7      GGAGTGCA--GTGGCAAGATCTCGGCTCACTGCAAGCTCGG--------CCTCCGGGGTTCACGCCATTCT--CCTGC---CTCAGCCTCCCGAGTAGC-------------------------------
hsa-mir-520b_13/7        AGCCTCCT--GAGTTAGCATCTGCAGGCAT-------------------GAACCACAACACCTGGTAACC---TTTGTATTTTTGGACACATGAGGT---------------------------------
hsa-mir-519e_13/7        GGAGTGCA--GTGGCATGATCTCGGCTCACTGCAACCTACG--------CCTTCCGGGTTCAAGCGATTCT--CCTGC---CTCAGCCTCCTGAGTAGCTGGGATTTCAGGTGCATGCC-----------
hsa-mir-518c_13/7        GGAGTGCA--GTGGCAGGATCTCCACTCACCGTG---------------------GAGTTCCAGTGATTCT--TTCAC---CTTAGCCTCGCAAATAGCTGGTATTAC----------------------
hsa-mir-520g_13/7        GCTGAGAA--TACAGGCA----TGAGCCACCACCGCGCGCCTCCC--------AAAGGCGTGAGCCATGGTGCGTGGCCATCAACACCTCTTACTT----------------------------------
hsa-mir-519d_13/7        ----------------------------------------------------------------------------------------------------------------------------------
hsa-mir-520h_13/7        CCTCAGCC--TCCTGGGTT---CAAGTGATTCTCCTGCCTCAGCC--------TCCTGAGT-AGCTAGGATTACAGGCATGTGCCACCA-----------------------------------------
hsa-mir-516b-1_13/7      GGCCCTGT--CTCTGACTT---TTGCGAACTGAGGAAGTAAC--------CTTTTGAAATTTA----CTCTGAAGTGTGAATGATTTTAG----------------------------------------
hsa-mir-526b_13/7        GTCTTTTTAGGGGTCTATGTTC-----------TGGATTCCAGAAAA---CATGCAAACAGAGCCAATAAATAGGTCTTTATTTTTGTCTCTATTTTAACCTGGTCAAGGAAAAT---------------
hsa-mir-520d_13/7        AAACCCC-----ATCTCTATTT-----------AAAAATACAAAAAA---TTAGCGAGGCGTGGTGGTG-------------------------------------------------------------
hsa-mir-516a-2_13/7      TTACTTGT--GTGTTTCTGCT------------CAAATTTTA--------TTAAGGAGCTTTTATGGCCTC--GCCTGTGGT------------------------------------------------
hsa-mir-524_13/7         TATTTTCAGCAGAGGCGGGGT-------------------------------------------------------------------------------------------------------------
hsa-mir-520f_13/7        CTCTCTAGCTCTGGATCTGCCTCTTGTAGGCCGGTCACGTAGACCAATCTTGTATGGGTGTCCAGTTGAGGATAATGGGT--------------------------------------------------
hsa-mir-520e_13/7        GTTCCAGAGACACCATTTGTGTTGGTCAAACAATGGCCTCCTATAAATTTAGTTTAATGAATCCAAACCATGTTTCCTTCATTAAGAGAGTAAATAAGCCTCCAAGTCTATCCAGTCTTTTTGAGACAGA
hsa-mir-517a_13/7        ATTTTGGATGCTCCCATCGGAACTATGTATCCCTGTGCTGGAACT-----CAAGTGAACACTCAGCTCAAAGTCC-------------------------------------------------------
hsa-mir-517b_13/7        CCTCTAGAGTCAGCCTAGACTCT-----------------------------------------------------------------------------------------------------------
hsa-mir-517c_13/7        CCTGTAGAGTCAGCCTAGACTGTGGTCTATCTTGGAGGATG-----------------------------------------------------------------------------------------

                         4031
hsa-mir-519b_13/7        -----------------------------------------------
hsa-mir-523_13/7         -----------------------------------------------
hsa-mir-518a-2_13/7      -----------------------------------------------
hsa-mir-519c_13/7        TGCAAT-----------------------------------------
hsa-mir-525_13/7         CTGGATTCCAGAAAACATGCAAACAGGGCCAGTAAATGCATCTTTAT
hsa-mir-518b_13/7        -----------------------------------------------
hsa-mir-520a_13/7        -----------------------------------------------
hsa-mir-518f_13/7        -----------------------------------------------
hsa-mir-522_13/7         -----------------------------------------------
hsa-mir-526a-1_13/7      -----------------------------------------------
hsa-mir-518e_13/7        -----------------------------------------------
hsa-mir-516a-1_13/7      -----------------------------------------------
hsa-mir-527_13/7         -----------------------------------------------
hsa-mir-526a-2_13/7      -----------------------------------------------
hsa-mir-518d_13/7        -----------------------------------------------
hsa-mir-521-2_13/7       -----------------------------------------------
hsa-mir-516b-2_13/7      -----------------------------------------------
hsa-mir-519a-1_13/7      -----------------------------------------------
hsa-mir-520c_58902519    -----------------------------------------------
hsa-mir-518a-1_13/7      -----------------------------------------------
hsa-mir-521-1_13/7       TTGGGGGGCTGTGGCAGAAGAACCT----------------------
hsa-mir-519a-2_13/7      -----------------------------------------------
hsa-mir-520b_13/7        -----------------------------------------------
hsa-mir-519e_13/7        -----------------------------------------------
hsa-mir-518c_13/7        -----------------------------------------------
hsa-mir-520g_13/7        -----------------------------------------------
hsa-mir-519d_13/7        -----------------------------------------------
hsa-mir-520h_13/7        -----------------------------------------------
hsa-mir-516b-1_13/7      -----------------------------------------------
hsa-mir-526b_13/7        -----------------------------------------------
hsa-mir-520d_13/7        -----------------------------------------------
hsa-mir-516a-2_13/7      -----------------------------------------------
hsa-mir-524_13/7         -----------------------------------------------
hsa-mir-520f_13/7        -----------------------------------------------
hsa-mir-520e_13/7        GCTTGGCTCTTGTCACTCAGGCTAGAGTGCAATGGTGCAATCTCAGC
hsa-mir-517a_13/7        -----------------------------------------------
hsa-mir-517b_13/7        -----------------------------------------------
hsa-mir-517c_13/7        -----------------------------------------------
```
